# Long-Term Heat Selection of the Coral Endosymbiont *Cladocopium* C1^acro^ (Symbiodiniaceae) Stabilizes Associated Bacterial Communities

**DOI:** 10.3390/ijms23094913

**Published:** 2022-04-28

**Authors:** Patrick Buerger, Ruby T. Vanstone, Justin Maire, Madeleine J. H. van Oppen

**Affiliations:** 1 Applied Biosciences, Macquarie University, North Ryde, NSW 2113, Australia; 2 School of BioSciences, The University of Melbourne, Parkville, VIC 3010, Australia; rtvanstone@hotmail.com (R.T.V.); justin.maire@unimelb.edu.au (J.M.); madeleine.van@unimelb.edu.au (M.J.H.v.O.); 3 Australian Institute of Marine Science, PMB #3, Townsville, QLD 4810, Australia

**Keywords:** Symbiodiniaceae, coral, microbiome, 16S rRNA metabarcoding, experimental evolution, thermal tolerance

## Abstract

Heat-tolerant strains of the coral endosymbiont, *Cladocopium* C1^acro^ (Symbiodiniaceae), have previously been developed via experimental evolution. Here, we examine physiological responses and bacterial community composition (using 16S rRNA gene metabarcoding) in cultures of 10 heat-evolved (SS) and 9 wild-type (WT) strains, which had been exposed for 6 years to 31 °C and 27 °C, respectively. We also examine whether the associated bacterial communities were affected by a three-week reciprocal transplantation to both temperatures. The SS strains had bacterial communities with lower diversities that showed more stability and lower variability when exposed to elevated temperatures compared with the WT strains. Amplicon sequence variants (ASVs) of the bacterial genera *Labrenzia*, *Algiphilus*, *Hyphobacterium* and *Roseitalea* were significantly more associated with the SS strains compared with the WT strains. WT strains showed higher abundance of ASVs assigned to the genera *Fabibacter* and *Tropicimonas*. We hypothesize that these compositional differences in associated bacterial communities between SS and WT strains also contribute to the thermal tolerance of the microalgae. Future research should explore functional potential between bacterial communities using metagenomics to unravel specific genomic adaptations.

## 1. Introduction

Coral mass bleaching events and mortality have increased over the last few decades due to the rise in frequency and severity of summer heat waves [1]. The thermal tolerance of corals largely depends on their functional symbiosis with microalgae in the family Symbiodiniaceae, which meet most of the coral’s energy requirements through photosynthate translocation. When seawater temperatures exceed the thermal threshold of this symbiosis, the microalgae are lost from the host’s tissue—a dysbiotic state referred to as coral bleaching. Often, excess reactive oxygen species (ROS) primarily originating from the microalgae and leaking into the host cells are referred to as one of the main physiological contributors to coral bleaching. During heat stress, ROS can overwhelm the antioxidant capacities of the symbiont and coral, which can lead to cellular malfunctions and coral bleaching ([2], but see [3]). If the increased ROS are not scavenged and the coral–microalgae symbiosis not re-established, most corals starve and eventually die as a result.

Scleractinian corals closely associate with a diversity of microbes including bacteria, archaea, fungi, protists and viruses [4] and form a consortium that is termed the coral holobiont [5]. There is increasing evidence that not only the symbiotic microalgae, but also the associated bacteria have important functions within the coral holobiont [6] contributing to nitrogen fixation [7,8], sulfur-cycling [9], antibiotic production [10,11], amino acid provisioning [6], and pathogen exclusion via occupying available niches [5,12]. Recent studies suggest bacteria may also play a role in the susceptibility of corals to bleaching. For example, corals of the species *Acropora hyacinthus* with higher thermal tolerance showed a more stable association with their bacterial communities compared with more thermally susceptible corals [13]. Furthermore, a meta-analysis of bacterial communities in corals has revealed a correlation between the composition of these communities and coral bleaching, supporting a role for bacteria in coral thermal tolerance [14].

Although the composition of coral-associated bacterial communities is well-studied, knowledge of the Symbiodiniaceae-associated bacterial consortia and their interactions is scarce [15]. Bacteria may perform important functions within the coral holobiont, for instance, it has been proposed that ROS produced during thermal stress can be neutralized by enzymes, such as superoxide dismutase [16], or carotenoids [17]⁠ produced by bacteria. Symbiodiniaceae cultures harbor both extracellular and intracellular bacteria, although their respective roles remain uninvestigated [18]. Lawson et al. [19] performed a baseline bacterial community characterization of a wide taxonomic range of Symbiodiniaceae cultures, named potential functions they could provide to the microalgae and identified core bacterial genera that are associated with every strain. More recently, Camp et al. [20] went further and subjected Symbiodiniaceae cultures to an acute heat stress experiment and found changes in relative bacterial abundance in response to heat stress. However, no common changes across Symbiodiniaceae strains that correlated with the heat stress treatment were found.

Here, we compare the physiological responses and bacterial communities associated with 19 closely related strains of the common Symbiodiniaceae species, *Cladocopium* C1^acro^ (formally known as type C1 [21]). A total of 10 of the 19 cultures were heat-evolved (SS) from 27 °C to 31 °C via experimental evolution in 2011 [22] and subsequently maintained at the elevated temperature for 6 years (~180 generations). As a result, the SS strains have increased their upper thermal tolerance and showed a reduced amount of ROS under elevated temperature compared with two wild-type strains (WT) that were kept at 27 °C [23]. A total of 3 of the ten SS strains were able to confer their increased thermal tolerance on coral larvae after establishing symbiosis [23]. The increased larval heat tolerance was accompanied by lower constitutive expression of algal photosynthesis genes, higher expression of genes associated with carbon fixation, and higher levels of expression of coral host heat stress response genes.

As a first step in exploring whether bacteria associated with the various microalgae strains may have contributed to their thermal tolerance and their ability to confer this trait on their coral host, we applied 16S rRNA gene metabarcoding to characterize the bacterial community composition in the SS and WT cultures of *Cladocopium* C1^acro^ after an acclimation period of six weeks at 27 °C. We then conducted a reciprocal transplant experiment (RTE) where the cultures were exposed to the WT and SS long-term culturing temperatures of ambient (27 °C) and elevated temperatures (31 °C) for three weeks, respectively, and assessed whether any changes occurred in their associated bacterial communities. Additionally, we show a physiological performance comparison between all available 10 SS strains and the 9 WT strains at ambient and elevated temperatures.

## 2. Results

### 2.1. Physiological Measurements in RTE

Parts of the physiological data have been published already in Buerger et al. [23], i.e., the physiological data for the SS strains and two of the WT strains (WT10, WT15). This current manuscript provides additional physiological data for the WT strains (WT11, WT12, WT13, WT14, WT16, WT17, WT18) in order to show a full performance comparison between all available *Cladocopium* C1^acro^ SS and WT strains (SS: 10 strains versus WT: 9 strains).

**Figure 1:**
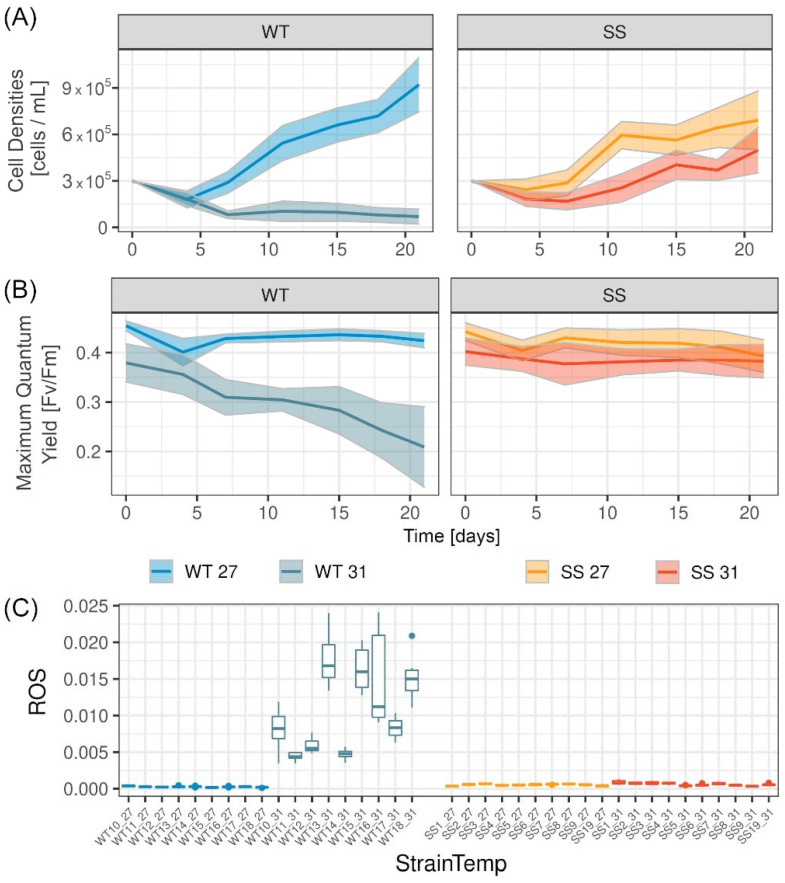
Physiological performance of wild-type and heat-evolved strains. (**A**) Cell densities [cells/mL] (**B**) maximum quantum yield [Fv/Fm] are shown with standard deviations from the mean. (**C**) Extracellularly measured reactive oxygen species (ROS) [arbitrary fluorescence unit] at day 21 is shown for the strain groups wild-type (WT) and the heat-evolved strain (SS) at both temperatures (27 °C and 31 °C).

At ambient temperatures (27 °C), over the three weeks both the heat-evolved (*n* = 10 strains) and the wild-type strains (*n* = 9 strains) increased in cell densities (Figure 1A), maintained their maximum quantum yield values (Figure 1B) and showed minimal signs of ROS in the culture media (Figure 1C). Although strains of both groups (SS and WT) increased in cell densities over time at ambient temperatures, the wild-type strains grew significantly better to 9.16 ± 1.62 × 10^5^ cells/mL on average compared with the heat-evolved strains which grew to 6.79 ± 1.61 × 10^5^ cells/mL (linear mixed effects model (LME) main effect *p*-value details in Table S2A–C). Nevertheless, some of the SS strains grew as quickly as the WT strains (cell densities at day 21, SS1: 9.2 ± 2.4 × 10^5^, SS5: 7.4 ± 1.3 × 10^5^, SS19: 9.3 ± 2.8 × 10^5^; compared with WT14: 7.4 ± 0.8 × 10^5^, WT15: 9.0 ± 2.2 × 10^5^, WT16: 8.0 ± 0.6 × 10^5^).

At elevated temperatures (31 °C), the WT strains showed significantly lower cell densities and maximum quantum yield values compared with the SS strains (Figure 1). On average across the strains at day 21, cell densities were 7 times higher for the heat-evolved strains (4.99 ± 1.47 × 10^5^ cells/mL) compared with the wild-type strains (0.70 ± 0.48 × 10^5^ cells/mL), maximum quantum yield values were 3.5 times higher in the heat-evolved strains (0.344 ± 0.038 Fv/Fm) compared with the wild-type (0.098 ± 0.120 Fv/Fm) and the amount of ROS in the culture medium was 17 times lower in the heat-evolved strains (0.0006 ± 0.0002 fluorescence dimensionless unit) compared with the wild-type strains (0.0105 ± 0.0058 fluorescence dimensionless unit).

### 2.2. Statistics of 16S rRNA Gene Metabarcoding Sequence Data

After demultiplexing, a total of 3,463,895 sequences remained across the successfully extracted 374 samples, with an average of 9413 sequences per sample (minimum of 3031 sequences and a maximum of 18,692 sequences in a sample). Although measures were taken to reduce contamination throughout the metabarcoding workflow, some contamination is common in metabarcoding studies and also occurred in this study. Seventy-six amplicon sequence variants (ASVs) were found in the negative controls. Thirty of these were removed from the dataset prior to further analysis due to their higher abundance (>40%) in the negative control samples compared with the algal samples. A total of 488 ASVs and 178 genera were present in the final dataset across all samples, whereas strains had on average 135 ASVs: SS@27 °C 121 ± 11 ASVs; SS@31 °C 128 ± 13 ASVs; WT@27 °C 143 ± 12 ASVs; WT@31 °C 149 ± 14 ASVs.

### 2.3. Differences in Alpha Diversity during RTE

**Figure 2:**
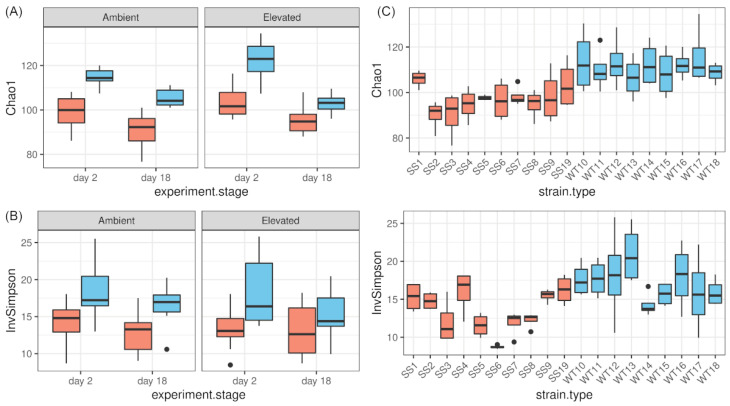
Alpha diversity based on the Chao1 and Inverse Simpson diversity indices for *Cladocopium* C1^acro^ associated bacterial communities of SS strains (orange) and WT strains (blue) in the RTE. Alpha diversity as (**A**) Chao1 and (**B**) Inverse Simpson index for bacterial communities of the microalgae groups at ambient, elevated temperature, and day 2 and day 18 of the 3-week RTE. (**C**) Chao1 and Inverse Simpson index are shown for the individual strains averaged across experimental stage and treatment.

Alpha diversity decreased for both groups (WT, SS) and treatments significantly throughout the course of the RTE (LME, significant main effect *experiment*.*stage* for Chao1 and Inverse Simpson index *p*-value < 0.001 and 0.017, respectively, Table S2D). However, the WT strains had a significantly higher alpha diversity with more observed ASVs (Chao1, Figure 1A) and more richness and diversity (Inverse Simpson, Figure 1B) compared with the SS bacterial communities (LME, main effect *group* for Chao1 and Inverse Simpson *p*-value < 0.001 and 0.002, respectively). The Chao1 and Inverse Simpson indices are shown for the individual strain in graphs that include both temperatures (Figure 2C).

Additionally, alpha diversity measured as Chao1 was dependent on the microalgal groups, treatment and experimental stage due to a significant interaction effect (LME, interaction: “group:temperature:experiment.stage” *p*-value < 0.043, Table S2D). Testing this interaction further by adjusting the model contrasts showed that the WT alpha diversity (Chao1) at elevated temperatures decreased significantly more between the start and the end of the RTE compared with the WT at ambient temperatures (adjusted model contrasts, *p*-value = 0.008) and compared with the SS strains at elevated (*p*-value = 0.004) and ambient temperatures (*p*-value = 0.006). This indicates that the number of observed ASVs decreased during the experiment the most in the WT strains at elevated temperatures. There was no significant interaction between the main effects for Inverse Simpson index.

### 2.4. Bacterial Community Differences among Cladocopium C1^acro^ Strains

**Figure 3:**
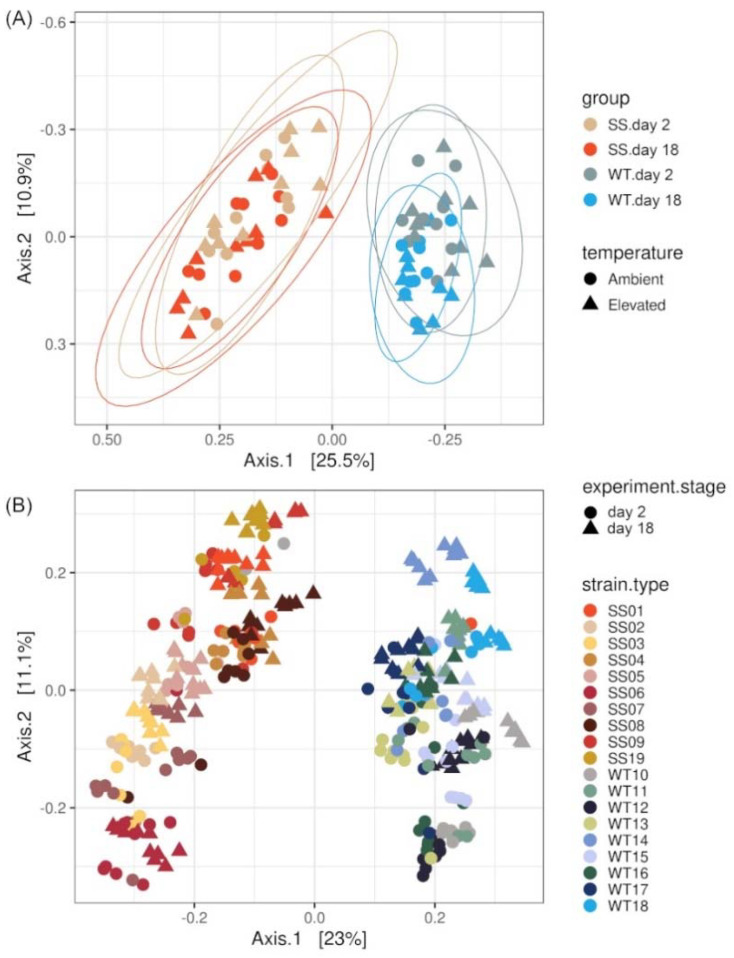
PCoA visualization using the Bray–Curtis dissimilarity separating samples by (**A**) their strain group (SS or WT) based on a reduced dataset with average values across strains. Data are shown for day 2 and day 18. The SS and the WT strains had different overall bacterial community composition. (**B**) The individual *Cladocopium* C1^acro^ strains are shown based on the full dataset and each SS and WT strain had a distinctive bacterial community fingerprint.

The WT and SS groups had similar dispersion to one another (betadisper, *p*-value = 0.552, Table S3), but significantly different bacterial community composition (beta diversity) based on Bray–Curtis distances (PERMANOVA, *p*-value < 0.001, Figure 3A, Table S2F). Additional beta diversity metrics also indicated differences between WT and SS strains, such as weighted UniFrac distances (PERMANOVA, *p*-value < 0.001, Figure S3, Table S2G) and Jaccard distances (PERMANOVA, *p*-value < 0.001, Figure S3, Table S2H).

A significant interaction term was detected for beta diversities (Bray–Curtis) “group:experiment.stage” (PERMANOVA, *p*-value = 0.034, Table S2F) with a significant difference in dispersal (betadisper, overall dispersal “experiment.stage”: *p*-value = 0.037, Table S3). The PCoA plot indicates that only the WT strains showed reduced dispersal at day 18 compared with day 2 of the RTE. In contrast, the SS microalgae showed a similar variability in community composition among their strains on day 2 and day 18 of the RTE.

Another significant interaction term was “group:temperature” (PERMANOVA, *p*-value = 0.032) with similar dispersal among samples (betadisper, overall dispersal “temperature”: *p*-value = 0.4, Table S3), indicating that the community composition between the WT and SS strains remained different (location-wise on the PcoA) throughout the RTE. No significantly different three-way interaction effect was observed (Bray–Curtis or weighted UniFrac).

Apart from the overall group differences, individual strain responses were visible in the data. For example, although the overall group dispersal of SS and WT had a similar dispersal (*p*-value see above), there were strain specific dispersal effects (betadisper, dispersal for “strain”, *p*-value = 0.003, Table S3) with the SS strains showing a lower variability in their community composition compared with the WT strains (Figure S4, distance to centroids for each strain). Furthermore, each WT and SS strain had its own variation of bacterial composition compared with the other strains of their group (distinct location on the beta diversity plot, as shown in the PCoA Figure 3B).

### 2.5. Description of Bacterial Taxonomic Affiliation Associated with WT and SS

**Figure 4:**
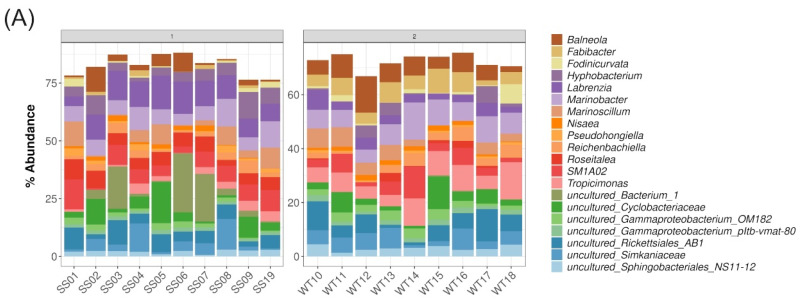

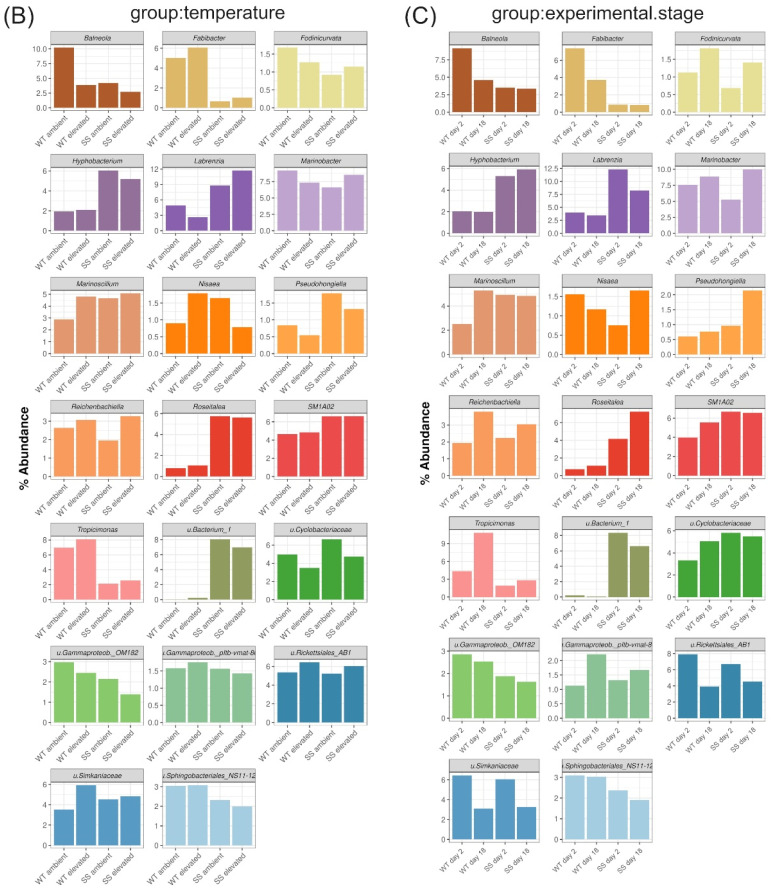
Barplot displaying the relative abundance [%] of the top 20 most abundant bacterial genera, which accounted for ~70–80% of the relative abundance among the strains. (**A**) Stacked barplot showing bacterial genera associated with the different strains across all time points and treatments. The remaining % to fill the bars to 100% belong to taxa not in the top 20 genera. Combined barplots show the interaction terms (**B**) “group:temperature”with combined time points and (**C**) “group:experiment.stage” with combined temperatures. If a genus assignment included only unassigned ASVs, the genus name was replaced with the next higher taxon level, e.g., for an uncultured/unassigned ASV of the Family_Simkaniaceae, written short “u.Simkaniaceae”. Number of ASVs per genera in Table S4.

The most dominant bacterial classes associated with all cultures (ones that accounted for more than 5% of the observed ASVs) were Alphaproteobacteria comprising on average 33% of the ASVs across all samples and groups (WT: 30.4%, SS: 35.5%), Bacteroidia (overall: 21%, WT: 23.7%, SS: 18.6%), Gammaproteobacteria (20%, WT: 21.8%, SS: 19.1%), Phycisphaerae (6%, WT: 4.7%, SS: 6.6%), Chlamydiae (5%, WT: 5.4%, SS: 5.3%) and Rhodothermia (5%, WT: 7.1%, SS: 3.6%) (Figure 4A). Exploration of the relative abundance of bacteria at the genus level revealed thermal-tolerance group specific patterns. Since no three-way interaction effect has been observed for beta-diversities, we present the taxonomic associations according to the two significant interactions of the main effects (“group:temperature” and “group:experiment.stage”).

For the WT strains, *Balneola* (26 ASVs, Table S4) had higher relative abundances at ambient temperatures (Figure 4B) and showed an increase on day 18 of the RTE (Figure 4C, from 4.6% to 9.2% in the WT, whereas the SS strains remain at ~3.4%). ASVs *Fabibacter* (8 ASVs) showed higher abundances in WT strains at both temperatures (average of WT: 5.5%, SS: 0.8%) (Figure 4B) and an increase on day 18 of the experiment (from 3.7% to 7.3% in the WT, whereas the SS strains remained at ~0.9%) (Figure 4C). *Tropicimonas* (2 ASVs) was higher across the treatments (average of WT: 7.5%, SS: 2.4%), but decreased on day 18 of the experiment in the WT strains (from 10.8% to 4.4% in the WT, whereas the SS strains remained at ~2.4%). In comparison, the SS strains had a higher relative abundance of *Hyphobacterium* (11 ASVs, WT: 2.0%, SS: 11.3%), *Labrenzia* (15 ASVs, WT: 3.8%, SS: 10.3%), and *Roseitalea* (2 ASVs, WT: 0.9%, SS: 5.7%) at both temperatures (Figure 4B). For the SS strains, ASVs of the genus *Labrenzia* increased from day 2 to day 18 of the experiment (8.3% to 12.3%), but that increase was marginal for the WT strains with 3.5% at day 2 and 4.0% at day 18 of the RTE.

Some bacteria of the Rickettsiales_AB1 (13 ASVs) and the family Simkaniaceae (10 ASVs) were more abundant in the elevated temperature treatment and on day 18 of the RTE for both the WT and SS strains. The most abundant genus across all samples was *Marinobacter* with 24 ASVs (relative abundance across the temperatures in WT: 8.4%, SS: 7.8%) on average (Figure S5) and the most diverse genus was *Balneola* with 26 ASVs (relative abundance in WT: 7.1%, SS: 3.5%).

### 2.6. Defining Core ASVs for SS and WT

**Figure 5:**
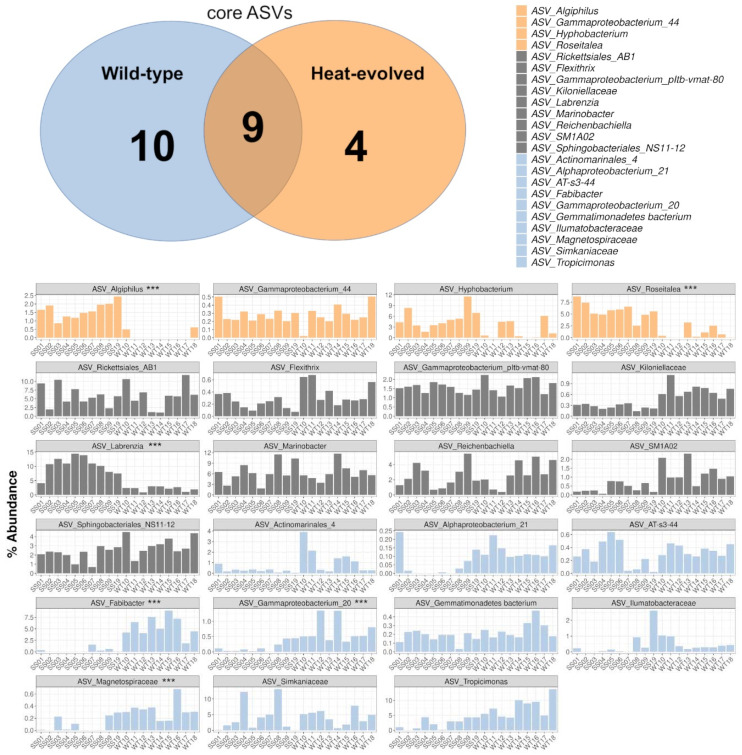
Core taxa in heat-evolved strains (SS) and wild-type (WT) strains. The Venn diagram shows the number of core ASVs for each strain group and the number of shared core ASVs across strains. Relative abundances are shown as bar plots for the core ASVs of the WT and SS strains. The color coding is indicative for the strain group in which the core ASV is detected (orange: SS strains; grey: shared core taxa between SS and WT; blue: WT strains). The three stars (***) next to the ASV genus assignment indicate a significantly different abundance between the WT and SS strains through the DESeq2 analysis, at significance threshold <0.001.

A total of 23 core ASVs were identified that were present in either all WT or SS samples, or both. Shared among the WT and SS samples were 9 out of the 23 core ASVs, whereas 10 and 4 ASVs were categorized as core only for the WT or SS samples, respectively (Figure 5).

Among the 10 core ASVs for the WT strains were ASVs of the genera *Tropicimonas* and *Fabibacter* (Figure 5). The SS strains had 4 associated core ASVs belonging to the genera *Algiphilus* (WT: 0.13%, SS: 1.66%), *Hyphobacterium* (ASV relative average abundance in all WT samples: 1.96%, SS: 5.40%), *Roseitalea* (WT: 0.90%, SS: 5.74%), and an uncultured Gammaproteobacterium (WT: 0.28%, SS: 0.30%). Part of the shared core taxa was one ASV assigned to the genus *Labrenzia* (relative abundance in WT: 2.18%, SS: 10.20%).

### 2.7. Statistical Differentiation of ASVs as Assessed with DESeq2

To obtain a more detailed understanding of the ASVs that are significantly enriched in one of the strain groups, a differential abundance analysis with DESeq2 was performed. A total of 99 ASVs were identified as differentially abundant between WT and SS with > 2 log2 fold-change (LFC) and a 0.001 *p*-value cutoff (Figure S6, supplementary data material). Enriched in the WT samples were 63 ASVs. These included, for example, core ASVs assigned to the genus *Fabibacter* (21-LFC, for relative abundance of core taxa see section before, *p*-value = 1.56 × 10^−18^), the family Magnetospiraceae (21-LFC, *p*-value = 9.66 × 10^−14^), and an ASV assigned to Gammaproteobateria (21-LFC, *p*-value = 4.75 × 10^−9^). Other significantly enriched taxa for the WT strains were, for example, an uncultured ASV assigned to the genus *Labrenzia* which had one of the highest log-fold changes (21-LFC, WT relative abundance: 1.51%, SS strains: 0.0001%, *p*-value = 2.75 × 10^−121^), an ASV of the genus *Balneola* (5-LFC, WT: 5.72% relative abundance, compared with SS strains: 0.54% relative abundance, *p*-value = 9.22 × 10^−13^) and an ASV of the genus *Marinobacter* (17-LFC, WT: 1.34%, SS strains: 0.027%, *p*-value = 2.72 × 10^−66^).

In comparison, enriched in the SS strains (versus WT) were 36 ASVs with > 2 LFC. Two of the SS core ASVs were significantly enriched in the SS strains, i.e., the *Algiphilus* core ASV (4-LCF, *p*-value = 4.24 × 10^−5^) and *Roseitalea* core ASV (3-LCF, *p*-value = 6.38 × 10^−10^). Other significantly enriched ASVs within the SS strains included, for example, an uncultured ASV assigned to *Fabibacter* (11-LCF, WT: 0.0002%, SS strains: 0.68%, *p*-value = 8.38 × 10^−30^) and an uncultured delta proteobacterium (9-LCF, WT: 0.0004%, SS strains: 0.29%, *p*-value = 1.28 × 10^−16^). In addition, the *Labrenzia* core ASV that was present in all samples for WT and SS strains was highly abundant in the SS strains and also significantly more enriched (2-LCF, WT: 2.18%, SS strains: 10.2%, *p*-value = 6.11 × 10^−17^).

We also analyzed relative ASV abundances between SS strains that have shown to confer their thermal tolerance to coral larvae (SS+: SS1, SS7, SS8) and strains that have not (SS−: SS2, SS3, SS4, SS5, SS6, SS9, SS19) [23]. The DESeq2 contrast of SS+ versus SS− strains showed 29 ASVs as significantly more abundant with > 2 LCF in one or the other SS group (Figure S7). However, only 4 of the ASVs met our criteria of being present in every strain of the enriched group (this criterium was only applied to the SS+ vs. SS− comparison due to the unbalanced design). These included an ASV enriched for SS+ of the genus *Turneriella* (24-LCF, SS+: 0.0608%, SS−: <0.0000%, *p*-value = 1.80 × 10^−21^). Enriched for SS− were ASVs assigned to an unknown genus of the family Magnetospiraceae which was also a core member of the WT strains (5-LCF, SS+: 0.006%, SS−: 0.132%, *p*-value = 2.85 × 10^−9^), an unknown genus of the family Cyclobacteriaceae (8-LCF, SS+: 0.04%, SS−: 5.89%, *p*-value = 2.14 × 10^−14^) and an ASV assigned to the genus *Balneola* (3-LCF, SS+: 0.42%, SS−: 3.48%, *p*-value < 0.0003).

## 3. Discussion

### 3.1. Physiological Performance of Wild-Type (WT) and Heat-Evolved (SS) Strains

Experimental evolution has the potential to fast-track thermal adaptation of coral-associated microbes [22,23,24]. Here, we show that the experimental evolution treatment resulted in SS strains with enhanced in vitro thermal performance. Although the WT strains grew better at ambient temperatures, some of the fastest growing SS strains (such as SS1, SS5 and SS19) grew as well as some of the slower growing WT strains (such as WT14, WT16 and WT17). Previously, the physiological performance of SS strains has only been compared with a small number of WT strains [22,23], whereas this is the first time that all available SS strains (*n* = 10) are compared with all available WT strains (*n* = 9 strains). Consistent with previous analyses [22,23], the SS strains showed significantly higher growth at elevated temperatures, maintained their photosynthetic activity and showed minimal amounts of reactive oxygen species compared with the WT strains.

### 3.2. Impacts of Thermal Selection on Bacterial Community Composition

Previous research has investigated how experimental evolution influences the adaptation of the coral associated Symbiodiniaceae itself [22,23,25]. Here we show for the first time that experimental evolution also affected other microbial organisms present in the culture aside from the microalgae.

The community composition difference between the WT and SS strains was largest when comparing beta diversities based on Bray–Curtis distances, which considers abundance data. Beta diversities measured in weighted UniFrac distances, which considers abundance data and phylogenetic relationships, also showed a significant but less prominent difference between the SS and the WT groups. Hence, most of the differences between the WT and the SS strains are based on the abundance of the taxa and presence/absence, whereas some of the taxonomic/phylogenetic distances are based on partial 16S rRNA sequences. At the same time, the overall number of observed bacterial ASVs and richness in the WT strains was higher compared with the SS strains (Chao1: +14.6%, Inverse Simpson index: +27.7%). This indicates a loss in the number of taxa for the SS strains, which is likely a response to the long-term experimental evolution treatment under elevated temperatures.

The stable adaptive state of the bacterial community of the SS strains at the higher temperature is further demonstrated during the 3-week RTE, where alpha diversity (Chao1) of the WT strains dropped significantly more at elevated temperatures compared with the SS strains (drop for WT: −18.9%, SS: −8.8%). In addition, we observed a reduction in the sample dispersal of beta diversity for the WT strains in response to the RTE but not for the SS strains, which were more stable during the experiment.

A reduction in alpha diversity was previously observed for wild-type cultures of other Symbiodiniaceae strains in response to short-term heat stress [20]. After 10 days of heat stress at 32 °C, bacterial richness (Inverse Simpson) was reduced for a thermally sensitive strain C1-123 by approximately 30%, whereas the bacterial richness of a more thermally tolerant strain D1a was more stable [20]. The differences in diversities in response to heat stress may originate from the availability of nutrients and resources within microalgae cultures, which can influence the interactions and abundance for some heterotrophic bacteria, for example due to cell lysis and metabolic adjustments [20]. Since the SS strains showed lower but more stable diversities in comparison with the WT, it suggests that the remaining taxa in the SS cultures show less mortality and metabolic adjustments in response to 31 °C compared with the WT strains.

### 3.3. Bacterial Communities Associated with WT and SS Strains

Different Symbiodiniaceae cultures are known to have different associated bacterial communities [18,19,20]. The dominant bacterial taxa in these assemblages may have important roles in nutrient provisioning to their hosts [20]. The C1-123 strain from Camp et al. [20], which is closely related to *Cladocopium* C1^acro^, was predominantly associated with *Actibacterium* (23%), and C1-124, another close relative, was dominated by *Hyphobacterium* (37%) and *Labrenzia* (22%) [20]. In the current study on *Cladocopium* C1^acro^, all strains were dominated by *Marinobacter* on average with 8.1% relative abundance across all strains. *Marinobacter* is frequently present in coral microbiomes [26,27,28] and in Symbiodiniaceae cultures [18,19,20], and has previously also been identified as a core microbiome member with a relative abundance of 3–5% across Symbiodiniaceae strains [19]. However, *Marinobacter* has in the previous studies not been identified as the most dominant genus. This shows that different strains in general, but also across laboratories, may have their own signature of dominant bacterial taxa.

Previously, several bacterial core taxa were reported to be shared by several *Cladocopium goreaui* strains. For example, at ambient conditions strain C1-123 had 25 core taxa and shared 8 of these with the strain C1-124 (37 core taxa), which were assessed from 8 replicates per strain [20]. Here we found fewer core ASVs among closely related strains of *Cladocopium* C1^acro^ that were derived from the same mother culture 6 years before this experiment. For the WT and the SS strains, we observed 19 and 13 core-associated ASVs, respectively, with 9 of them shared between the two groups. In our study, these core taxa were however defined across ~190 replicates for each of the WT and SS group. The hurdle for a core ASV to be present or detected in every of the ~190 replicates might have been too stringent to define meaningful core bacteria among the groups. This also indicates that most of the taxa were not detected in every replicate of a particular group. Since the strains have been established only 6 years prior to this experiment, it shows that this is enough time to create some amount of diversification in the bacterial community composition among laboratory grown strains.

### 3.4. Functional Potential

The WT bacterial communities were characterized by higher relative abundances of ASVs assigned to the genera *Tropicimonas* and *Fabibacter*, whereas both genera were mostly absent in the SS strains. Representatives of both genera were identified as core ASVs for the WT strains. *Tropicimonas* is a member of the Rhodobacteraceae family and has been reported as a component of the coral holobiont in both healthy and diseased corals (e.g., [29,30]). *Fabibacter* is a member of the Cyclobacteriaceae family and has been reported from other Symbiodiniaceae cultures [19].

In comparison, the SS strains had a higher abundance of genera such as *Roseitalea* and *Hyphobacterium* (both also with core ASVs for SS strains). In addition, the SS strains had a core ASV assigned to *Algiphilus*. *Hyphobacterium* of the family Hyphomonadaceae has previously been identified as the dominant taxon in the Symbiodiniaceae strain *Durusdinium trenchii* [19,20]. *Roseitalea* (order Rhizobiales) has been previously identified as a highly abundant taxon across 7 Symbiodiniaceae strains [18]. This presence/absence of taxa between the WT and SS strains may further translate into functional differences.

Further functional differences are suggested through abundance data of taxa in WT and SS strains. For example, although the genus *Labrenzia* had a core ASV present among all replicates of both WT and SS, the 15 *Labrenzia* ASVs were present at higher abundances for the SS strains compared with the WT strains (SS: 10.3%, WT: 3.8%). The generally high abundance of *Labrenzia* in our *Cladocopium* C1^acro^ cultures was expected given that it is usually found in relatively high abundance in Symbiodiniaceae cultures. For example, *Labrenzia* was previously identified as a core microbiome member for both corals and Symbiodiniaceae [19,31,32]. In other studies, *Labrenzia* had a 22% relative abundance in the bacterial community of the *C. goreaui* strain C1-124 [20] and 38.4% on average across cultures from 5 Symbiodiniaceae genera [19]. It has been detected in 11 different Symbiodiniaceae strains in loosely, closely and intracellular association [18]. These consistently high abundances for *Labrenzia* led to the hypothesis that it plays crucial, and potentially beneficial roles in cultured Symbiodiniaceae. Since some *Labrenzia* strains produce dimethylsulfoniopropionate [33,34], a known antioxidant that may be involved in Symbiodiniaceae heat tolerance [35], it is possible that the observed *Labrenzia* ASVs also have a functional role in the thermal tolerance of the SS *Cladocopium* C1^acro^ strains.

Additionally, we compared the ASV abundances among SS strains that previously have been shown to confer their thermal tolerance on coral larvae (SS+) with strains that have not (SS−) [23]. The SS+ strains showed a relatively higher abundance of an ASV assigned to the genus *Turneriella* and a relatively lower abundance of three ASVs belonging to the genera *Balneola*, and the Family Magnetospiraceae and Cyclobacteriaceae compared with the SS− strains. It is possible that these ASV abundance differences may have contribution to the characteristics of the SS+ and SS− strains. Due to the overall general similarity between the SS+ and SS− strain this is, however, highly speculative at this stage.

Aside from the compositional changes between WT and SS strains, we cannot exclude additional functional differences between the respective microbiomes of SS and WT that are hidden in genomic adjustments. Due to the long relative exposure to increased temperatures of the SS strains, we assume that the bacterial genomes have evolved along with the Symbiodiniaceae and that mutations leading to different phenotypes which perform well under the increased temperature will have spread [24,36,37]. Indeed, bacteria of the same strains can show distinct phenotypes but may still be distinguishable with the 16S rRNA marker gene. For example, some bacterial strains isolated from the coral model organism *Exaiptasia diaphana* have shown higher antioxidant capacities compared with strains of the same genus that were indistinguishable with partial 16S rRNA sequencing targeting the V5-V6 region [38,39], which is the same region that we targeted in this current paper. Hence, a community analysis based on abundance data with a generalized and relatively short marker gene may not reveal most functional differences between bacterial strains that are associated with experimental groups. Therefore, a metagenomic approach comparing bacterial genomes associated with different Symbiodiniaceae cultures is required to uncover genomic adaptations of taxa that are identical in their 16S rRNA gene sequence.

Experimental evolution can be used as a tool to enhance the thermal tolerance of microalgae and bacteria, although this has not yet been tested for coral- or Symbiodiniaceae-associated bacteria. This study provides novel insight into the bacterial communities associated with SS Cladocopium C1^acro^ and demonstrates that their bacterial community composition differs from the WT strains. We show that the bacterial communities of the SS strains are less diverse but more stable in response to acute changes in temperature. This in combination with the relatively high abundance of *Labrenzia* in the SS strains suggests a functional role for bacteria in Symbiodiniaceae thermal tolerance. These findings call for further functional analyses, genomic comparisons of bacteria associated with Symbiodiniaceae strains and to further test the performance of the heat-evolved bacterial strains in symbiosis with corals

## 4. Methods and Materials

### 4.1. Symbiodiniaceae Sample Collection and Long-Term Thermal Selection

The nineteen strains used in this study were derived from the same monoclonal mother culture, maintained at the Australian Institute of Marine Science, Townsville, Australia (culture number SCF055-01, Table S1). In brief, *Cladocopium* C1^acro^ cells were isolated from a colony of the coral species, *Acropora tenuis*, from Nelly Bay, Magnetic Island near Townsville, Australia in 2010. A monoclonal culture was subsequently created by plating an aliquot on agar and picking cells from a single colony forming unit, as described in Chakravarti et al. (2017). The monoclonal mother culture was characterized based on the ITS2 rDNA region (GenBank accession number AB778664.1). Starting in 2011, ten strains were subsequently heat-evolved (SS strains) through a rachet experiment [40] in which the thermal stress level was increased in a stepwise fashion and only populations that showed growth were transferred to the next rachet (a higher temperature). After the ratchet experiment was completed and ~6 years of growing at their respective temperature treatments, nine wild-type cultures (WT) which had been maintained at 27 °C and the ten SS were subjected to our reciprocal transplant experiment as reported in Buerger et al. [23].

### 4.2. Design of the Reciprocal Transplant Experiment (RTE)

The samples in this study are derived from the experiment reported in [23]. We describe the experimental design briefly below; see [23] for further experimental details.

The 9 WT and 10 SS cultures were exposed to a pre-acclimation period of six weeks at 27 °C to reset all culture conditions to the same temperature. Our reasoning was that acclimation to the same temperature will reveal stable rather than ephemeral differences among the cultures. Subsequently, the cultures were split into batches for a 21-day long reciprocal transplant experiment (RTE) with a starting cell density of 300,000 cells per mL and kept at 31 °C or 27 °C for 3 weeks (i.e., WT@27, SS@27, WT@31 and SS@31). We prepared samples to assess the bacterial community of the algal strains by dislodging algal cells from the walls of the culture containers and homogenizing the culture by vortexing for 1 min, followed by snap-freezing a 1 mL aliquot in liquid nitrogen. Samples were collected from different sets of flasks at two time-points: day 2 and day 18 of the 3-week RTE and stored at −80 °C until further processing. We collected five samples, each from different flasks, for each strain at both temperatures (31 °C and 27 °C; day 2 and day 18). WT with 9 strains × 2 temperatures × 2 time-points × 5 replicates = 180; and the SS with 10 strains × 2 temperatures × 2 time-points × 5 replicates = 200 (total *n* = 380).

Throughout the experiment, the cultures were kept in 1X Daigo’s IMK medium for Marine Microalgae (Nihon Pharmaceutical Co., Ltd., Tokyo, Japan) dissolved in sterile seawater (natural seawater, collected locally, autoclaved and 0.2 µm filtered). The cultures were maintained in two temperature controlled incubators at 27 °C ± 1°C and 31 °C ± 1 °C and at a light intensity of 65 ± 10 μmol photons m^−2^ s^−1^ (Sylvania FHO24W/T5/865 fluorescent tubes) under a 14:10 light:dark cycle in 25 cm^2^ flasks (Corning; Merck, Bayswater, Australia).

### 4.3. Physiological Measurements

Some of the physiological data are already published in [23], i.e., the data of the SS strains (SS1, SS2, SS3, SS4, SS5, SS6, SS7, SS8, SS9, SS19) and two WT strains (WT10 previously referred to as WT1, and WT15 previously referred to as WT2). The current manuscript provides additional physiological data for all available wild-types strains (additional data provided here: WT11, WT12, WT13, WT14, WT16, WT17, WT18) to better show and compare the physiological performance between the different microalgae groups.

The physiological response of the *Cladocopium* C1^acro^ strains to the RTE was monitored through measurements recording cell densities, photosynthetic activity and amount of reactive oxygen species in the culture medium. Samples were measured twice per week (at day 4, 7, 11, 15, 18 and 21) for cell densities and photosynthetic activity. For reactive oxygen species measurements, samples were only measured at the end of the experiment (day 21).

To collect the cell density data, cells were counted with an automated cell counter (Countess II FL Automated Cell Counter, Life Technologies, Thermo Fisher Scientific Australia Pty Ltd., Scoresby, Australia) with two technical replicates for each time-point. To maintain count accuracy, cell densities below 100,000 cells/mL were re-counted manually under an inverted fluorescence microscope and a hemocytometer with a 20× magnification.

For the photosynthetic data collection, the cultures were measured with an imaging pulse-amplitude modulation chlorophyll fluorometer (IPAM M-series, Walz, Germany). The maximum quantum yield of photosystem II was taken after dark-adaptation of the samples for 2-h on three-point locations with the parameter: measurement intensity 6, saturation pulse 10, gain 2, damping 2.

Reactive oxygen species measurements were taken from the culture medium to measure ROS that leaked from the microalgae. Samples of 1 mL were collected from the cultures and centrifuged for 5 min at 3000× *g*. Three technical replicates of 250 µL were transferred to a 96-well plate with 0.5 µL CellROX Orange stain (5 µM final concentration at 2.5 mM stock concentration) and incubated for 30 min at 37 °C. The plates were measured with a plate reader (Synergy H4, BioTek, Agilent, Santa Clara, CA, USA) at 545 nm absorption and 565 nm emission. Measurements across plates were normalized according to blanks, empty cells and cell numbers/mL [23].

### 4.4. DNA Extraction and 16S rRNA Gene Metabarcoding

DNA extractions were performed on the snap frozen 1 mL culture samples with a salting-out method. In brief, we added 0.75 mL of extraction buffer (20 mL extraction buffer: MiliQ 11.6 mL, Tris 2 mL at 1.0 M, EDTA 4 mL at 0.5 M, NaCl 400 µL at 5.0 M, SDS 2 mL at 10%) to the frozen culture sample and let it thaw on ice. Subsequently, we added 10 µL of 20 mg/mL lysozyme and incubated the samples for 15 min at room temperature. 100 mg of sterile glass beads (cat. number G8772, Merck, Bayswater, Australia) were added, and the samples were bead-beaten for 30 s at 30 Hz (Qiagen Tissue-Lyser II). To precipitate cell debris, we added 200 µL of 5 M KOAc to the tubes, incubated the mix on ice for 30 min and spun the tubes at max speed in a bench top centrifuge for 15 min. The supernatant was then transferred to a new tube and treated with 10 µL of 10 mg/mL RNase-A at 37 °C for 30 min. The DNA was precipitated by adding 700 µL isopropanol with an incubation of 15 min at room temperature. After spinning the samples again at max speed for 15 min, the supernatant was removed, the DNA pellet was washed twice with 150 µL of 70% ethanol and the final DNA airdried and resuspended in 25 µL MiliQ. DNA was stored at −20 °C until further processing.

The V5-V6 region of 16S rRNA was amplified using the polymerase chain reaction (PCR) and used for metabarcoding of the bacterial community. The following primers were used, 784F 5′ **TCGTCGGCAGCGTCAGATGTGTATAAGAGACAG** AGGATTAGATACCCTGGTA 3′ and 1061R 5′ **GTCTCGTGGGCTCGGAGATGTGTATAAGAGACAG** CRRCACGAGCTGACGAC 3′—the Illumina adapters are in bold. The PCR was performed in triplicate 10 µL reaction volumes, resulting in 30 µL pooled PCR product to buffer any bias created in a single PCR reaction. Each reaction consisted of 5.2 µL of MyTaq Red MasterMix (Bioline, Eveleigh, Australia), 0.2 µL of each primer (2 µM stock), 3.4 µL of nuclease-free water (Thermo Fisher Scientific Australia Pty Ltd., Scoresby, Australia), and 1 µL of DNA template. The PCR steps used were initial denaturation at 95 °C for 5 min followed by 30 amplification cycles (denaturation at 95 °C for 40 s, annealing at 55 °C for 40 s and extension at 72 °C for 1 min), and a final extension step of 7 min at 72 °C. The amplified product with the Illumina overhang adapters was sent to Ramaciotti Centre for Genomics, Australia for cleaning, library preparation and sequencing using the Illumina MiSeq platform with a V2 chemistry and 2 × 250 bp paired end reads. All processed sequences have been submitted to NCBI Genbank, detailed information regarding accession numbers (ON361601–ON362088) in Supplementary Materials.

### 4.5. Statistical Analysis

The individual strain replicates (#5 per strain) were averaged to one data entry to allow group comparisons between SS and WT strains without pseudoreplication. Hence, the number of samples was reduced for statistical analyses from #380 to #76.

Physiological data of cell densities and maximum quantum yield were analyzed for statistical differences in trends over time through a linear mixed effects model (LME) with random intercepts that accounted for strain variability as a random effect (R package *nlme*). Fixed factors were “group” (levels: WT, SS), “temperature” (levels: ambient, elevated) with “time” as a numeric variable (days: 4, 7, 11, 15, 18, 21). Model assumptions of normality, homogeneity, and equal variance were tested accordingly and met (Figure S1). The data of reactive oxygen species was analyzed as described above, except that the data was log-transformed in order to meet model assumptions and that “time” was not considered as a factor because ROS measurements were taken only at the end of the experiment. R-code and data files for all models and analyses are provided in the Supplementary Materials.

For the microbial community analysis, high-quality sequences with a phred score ≥30 were included in the downstream analyses using the quantitative insights in microbial ecology (QIIME2 version 2019.1) software package pipeline [41]. The sequences were trimmed to remove remainders of 16S rRNA primers using the QIIME2 plugin demux and dereplicated to enhance computational power. Then, the paired-end sequences were merged, potential chimeras were filtered out and amplicon sequence variants (ASVs) were assigned using the Deblur plugin [42] with a sequence similarity threshold of 99% [43]. All ASVs were identified taxonomically with the QIIME integrated SILVA database, version 132 [44] and the relative taxon abundances were calculated. Output tables such as ASV table, taxonomic table, phylogenetic tree and metadata were imported into RStudio v 3.5.3 and analyzed using the R package phyloseq [45].

Mitochondrial and chloroplast sequences were filtered out of the dataset, followed by the removal of taxa which had a minimum overall relative abundance of less than 10^−5^ to prevent the inclusion of sequencing errors. Contaminants were identified as any ASV that was 40% more prevalent in the control samples than in the *Cladocopium* C1^acro^ samples and removed from the dataset (supplementary data material). Rarefaction curves were generated to show that the samples have been sequenced deep enough to cover their diversity adequately (Figure S2).

Alpha diversity was measured using Chao1 and Inverse Simpson diversity indices [46]. The variation in alpha diversity community compositions was statistically tested using generalized linear mixed effects models with the factors “group” (levels: WT, SS) and “temperature” (levels: ambient, elevated) and with “experiment.stage” as a numeric variable (levels: day 2 and day 18 of the 3-week RTE coded in the model as 1 and 2, respectively). Strains were included in the model as a random effect. Model assumptions were validated by plotting fitted values versus residuals (homogeneity of variances), a histogram of the residuals with and a Q-Q plot (checking normal distribution) and residuals of the respective factors to check for data independence (Figure S1).

Beta diversity, i.e., differences in community composition based on taxon abundances, was measured on the raw data using a Bray–Curtis dissimilarity matrix (abundance data), weighted UniFrac distances (phylogenetic) and Jaccard dissimilarity index (presence/absence). A principal coordinate analysis (PCoA) was conducted to show the variation in community composition among samples. For both distance measures, the variation in community composition was statistically tested using a three-way PERMANOVA with “group”, “temperature”, “experimental.stage” as fixed factors. The data were then tested for multivariate homogeneity of group variances according to the dispersal of the respective sampling groups with a permutation test (function betadisper, vegan R package).

Core ASV associations were determined according to relative abundances across the entire dataset (#380 samples) and defined as an ASV that was present in every biological replicate [19]. Core ASVs were first determined for every strain, then the overall core ASVs were identified for the two groups WT and SS.

To identify ASVs that had a significant differential abundance between the strain groups, we used a pairwise abundance comparison with DESeq2 [47]. The DESeq2 model was adjusted with its contrasts for the respective groups to compare [WT versus SS]. We also compared the abundances of heat-evolved strains that conferred their thermal tolerance into the symbiosis with coral larvae (SS+: SS1, SS7, SS8) with the ASV abundances of strains that did not (SS−: SS2, SS3, SS4, SS5, SS6, SS9, SS19) [DESeq2 contrast: SS+ versus SS−]. To prevent false positives, we implemented a minimum log2 fold-change (LFC) threshold of >±2 LFC with a significance threshold of 0.01 to consider ASVs as significantly different abundant between groups. For the SS+ vs. SS− comparison, the enriched ASV had to be abundant in every strain of the enriched group, due to the unbalanced comparison.

## Data Availability

All data and statistics code is available in the Supplementary Materials. Sequences have been uploaded to NCBI (accession numbers ON361601–ON362088).

## Supplementary Materials

The following supporting information can be downloaded at: https://www.mdpi.com/article/10.3390/ijms23094913/s1.
