# Supplementary material for: Long-Term Heat Selection of the Coral Endosymbiont Cladocopium C1acro (Symbiodiniaceae) Stabilizes Associated Bacterial Communities
Source: Int J Mol Sci. 2022 Apr 28;23(9):4913. doi: 10.3390/ijms23094913 (PMC9101544; doi:10.3390/ijms23094913)
Supplement: Supplementary file 1 [file ijms-23-04913-s001.zip › MANUSCRIPT_DRAFT_PB_20220329_Supplementary.pdf]

## SUPPLEMENTARY MATERIAL

**Table S1. Details of wild-type (WT) and heat-evolved (SS) *C. goreaui* Symbiodiniaceae strains.** The culture collection code refers to the reference number at the Australian Institute of Marine Science (AIMS). [°C] indicates the permanent culture temperature of the strains. The column *"Conferring tolerance?"* refers to the experiment Buerger et al., [23] showing that some strains can increase the thermal tolerance of coral larvae. Other studies that studied the strains are reference in the last column "*References*".

| Strain ID | previous strain ID | Culture collection code (AIMS) | Type | °C | Strain | Conferring tolerance? | References |
|---|---|---|---|---|---|---|---|
| SS1 | - | SCF055-01.01 | C1 | 31 | SS | Yes | 1, 4 |
| SS2 | - | SCF055-01.02 | C1 | 31 | SS | No | 1, 4 |
| SS3 | - | SCF055-01.03 | C1 | 31 | SS | No | 1, 4 |
| SS4 | - | SCF055-01.04 | C1 | 31 | SS | No | 1, 2, 3, 4 |
| SS5 | - | SCF055-01.05 | C1 | 31 | SS | No | 1, 4 |
| SS6 | - | SCF055-01.06 | C1 | 31 | SS | No | 1, 4 |
| SS7 | - | SCF055-01.07 | C1 | 31 | SS | Yes | 1, 4 |
| SS8 | - | SCF055-01.08 | C1 | 31 | SS | Yes | 1, 4 |
| SS9 | - | SCF055-01.09 | C1 | 31 | SS | No | 1, 4 |
| SS19 | SS10 | SCF055-01.19 | C1 | 31 | SS | No | 1, 4 |
| WT10 | WT1 | SCF055-01.10 | C1 | 27 | WT | No | 1, 2, 3, 4 |
| WT11 | - | SCF055-01.11 | C1 | 27 | WT | No | 4 |
| WT12 | - | SCF055-01.12 | C1 | 27 | WT | No | 4 |
| WT13 | - | SCF055-01.13 | C1 | 27 | WT | No | 4 |
| WT14 | - | SCF055-01.14 | C1 | 27 | WT | No | 4 |
| WT15 | WT2 | SCF055-01.15 | C1 | 27 | WT | No | 1, 4 |
| WT16 | - | SCF055-01.16 | C1 | 27 | WT | No | 4 |
| WT17 | - | SCF055-01.17 | C1 | 27 | WT | No | 4 |
| WT18 | - | SCF055-01.18 | C1 | 27 | WT | No | 4 |

*** Table references:**

1 = Buerger et al., 2020 (tested individual strains after ~180 generations of experimental evolution)
2 = Chakravarti et al., 2017 (after ~103 generations)
3 = Chakravarti et al., 2020 (after ~103 generations)
4 = Quigley et al., 2021 (used a mixture of all strains, after ~51 generations)

Note: generation times are calculated from an experimental evolution start in year 2011.

**Table S2 Statistic outputs.** Tables are shown for generalized linear mixed effects models (A) cell densities, (B) maximum quantum yield, (C) reactive oxygen species (ROS), (D) Chao1, (E) Inverse Simpson index, (F) beta diversity Bray-Curtis distances and (G) beta diversity weighted UniFrac. Highlighted in grey are significant main effects and their interactions with a significance threshold of < 0.05.

| **(A) Cell Densities** | **numDF** | **denDF** | **F-value** | **p-value** |
|---|---|---|---|---|
| (Intercept) | 1 | 203 | 1701.59793 | < 0.001 |
| Group | 1 | 17 | 20.2399748 | < 0.001 |
| Time | 1 | 203 | 560.987117 | < 0.001 |
| Temperature | 1 | 203 | 877.330967 | < 0.001 |
| Group:Time | 1 | 203 | 6.43970983 | 0.012 |
| Group:Temperature | 1 | 203 | 149.039057 | < 0.001 |
| Time:Temperature | 1 | 203 | 217.076091 | < 0.001 |
| Group:Time:Temperature | 1 | 203 | 118.383852 | < 0.001 |

| **(B) YIELD** | **numDF** | **denDF** | **F-value** | **p-value** |
|---|---|---|---|---|
| (Intercept) | 1 | 200 | 5468.47155 | < 0.001 |
| Group | 1 | 17 | 16.934604 | < 0.001 |
| Time | 1 | 200 | 36.2477889 | 8.18E-09 |
| Temperature | 1 | 200 | 541.897578 | < 0.001 |
| Group:Time | 1 | 200 | 22.4694039 | 4.05E-06 |
| Group:Temperature | 1 | 200 | 247.230736 | < 0.001 |
| Time:Temperature | 1 | 200 | 39.0675843 | 2.43E-09 |
| Group:Time:Temperature | 1 | 200 | 66.4627461 | 3.85E-14 |

| **(C) ROS** | **numDF** | **denDF** | **F-value** | **p-value** |
|---|---|---|---|---|
| (Intercept) | 1 | 17 | 15109.7807 | < 0.001 |
| Group | 1 | 17 | 81.8069608 | 6.60E-08 |
| Temperature | 1 | 17 | 236.945857 | 2.05E-11 |
| Group:Temperature | 1 | 17 | 230.24707 | 2.58E-11 |

| **(D) CHAO1** | **numDF** | **denDF** | **F-value** | **p-value** |
|---|---|---|---|---|
| (Intercept) | 1 | 51 | 11659.9591 | < 0.001 |
| group | 1 | 17 | 54.2193125 | 1.11E-06 |
| temperature | 1 | 51 | 7.04580407 | 0.0106 |
| experiment.stage | 1 | 51 | 75.8595842 | 1.15E-11 |
| group:temperature | 1 | 51 | 0.57352107 | 0.452 |
| group:experiment.stage | 1 | 51 | 4.72807369 | 0.034 |
| temperature:experiment.stage | 1 | 51 | 3.28854473 | 0.075 |
| group:temperature:experiment.stage | 1 | 51 | 4.3259732 | 0.043 |

| **(E) Inverse Simpson** | **numDF** | **denDF** | **F-value** | **p-value** |
|---|---|---|---|---|
| (Intercept) | 1 | 51 | 863.9541 | 0 |
| group | 1 | 17 | 12.94839 | 0.002217 |
| temperature | 1 | 51 | 0.725071 | 0.398466 |
| experiment.stage | 1 | 51 | 6.03561 | 0.017463 |
| group:temperature | 1 | 51 | 0.025326 | 0.874186 |
| group:experiment.stage | 1 | 51 | 1.726375 | 0.194754 |
| temperature:experiment.stage | 1 | 51 | 0.012241 | 0.912335 |
| group:temperature:experiment.stage | 1 | 51 | 0.863663 | 0.357093 |

| **(F) Beta diversity Bray Curtis** | **DF** | **SumOfSqs** | **R2** | **F** | **Pr(>F)** |
|---|---|---|---|---|---|
| experiment.stage | 1 | 0.6881 | 0.04947 | 5.4714 | 0.001 |
| group | 1 | 3.24 | 0.23293 | 25.7635 | 0.001 |

| | | | | | |
|---|---|---|---|---|---|
| temperature | 1 | 0.3917 | 0.02816 | 3.1145 | 0.004 |
| experiment.stage:group | 1 | 0.252 | 0.01812 | 2.0042 | 0.034 |
| experiment.stage:temperature | 1 | 0.4348 | 0.03126 | 3.4575 | 0.002 |
| group:temperature | 1 | 0.2511 | 0.01805 | 1.9969 | 0.032 |
| experiment.stage:group:temperature | 1 | 0.1004 | 0.00722 | 0.7984 | 0.643 |
| Residual | 68 | 8.5515 | 0.61479 | | |
| Total | 75 | 13.9096 | 1 | | |

| **(G) Beta diversity weighted UniFrac** | **DF** | **SumOfSqs** | **R2** | **F** | **Pr(>F)** |
|---|---|---|---|---|---|
| group | 1 | 0.31704 | 0.11777 | 12.1349 | 0.001 |
| experiment.stage | 1 | 0.39956 | 0.14842 | 15.2935 | 0.001 |
| temperature | 1 | 0.06533 | 0.02427 | 2.5004 | 0.027 |
| group:experiment.stage | 1 | 0.01358 | 0.00504 | 0.5198 | 0.832 |
| group:temperature | 1 | 0.03293 | 0.01223 | 1.2603 | 0.252 |
| experiment.stage:temperature | 1 | 0.07632 | 0.02835 | 2.9211 | 0.007 |
| group:experiment.stage:temperature | 1 | 0.01079 | 0.00401 | 0.4131 | 0.909 |
| Residual | 68 | 1.77659 | 0.65992 | | |
| Total | 75 | 2.69214 | 1 | | |

| **(H) Beta diversity Jaccard** | **DF** | **SumOfSqs** | **R2** | **F** | **Pr(>F)** |
|---|---|---|---|---|---|
| group | 1 | 3.3391 | 0.15891 | 15.2671 | 0.001 |
| experiment.stage | 1 | 0.7931 | 0.03774 | 3.626 | 0.003 |
| temperature | 1 | 0.4892 | 0.02328 | 2.2369 | 0.005 |
| group:experiment.stage | 1 | 0.4079 | 0.01941 | 1.865 | 0.02 |
| group:temperature | 1 | 0.3826 | 0.01821 | 1.7496 | 0.023 |
| experiment.stage:temperature | 1 | 0.511 | 0.02432 | 2.3365 | 0.004 |
| group:experiment.stage:temperature | 1 | 0.2167 | 0.01031 | 0.9909 | 0.446 |
| Residual | 68 | 14.873 | 0.70780 | | |
| Total | 75 | 21.012 | 1.00000 | | |

**Table S3 Number of ASVs per genus.** ASV numbers are listed across al strains. Total number of ASVs is 488, total number of observed genera 178. Genera not listed in this table are with an ASV count of 1.

| # | Genus | # of ASVs | # | Genus | # of ASVs |
|---|---|---|---|---|---|
| **1** | *Acuticoccus* | 2 | **41** | *uncult_Cellvibrionales_BD2-7* | 2 |
| **2** | *Alcanivorax* | 4 | **42** | *uncult_Crocinitomicaceae* | 2 |
| **3** | *Aliikangiella* | 4 | **43** | *uncult_Cryomorphaceae* | 11 |
| **4** | *AT-s3-44* | 2 | **44** | *uncult_Cyclobacteriaceae* | 25 |
| **5** | *Balneola* | 26 | **45** | *uncult_Dehalococcoidia_SAR202* | 2 |
| **6** | *Bdellovibrio* | 8 | **46** | *uncult_Deltaproteobacterium_NB1-j* | 8 |
| **7** | *Blastopirellula* | 2 | **47** | *uncult_Flavobacteriales* | 2 |
| **8** | *Candidatus Tenderia* | 3 | **48** | *uncult_Flavobacteriales_NS9* | 2 |
| **9** | *Crocinitomix* | 6 | **49** | *uncult_Gaiellales* | 3 |
| **10** | *Ekhidna* | 3 | **50** | *uncult_Gammaproteobact._Ga0077536* | 2 |
| **11** | *Fabibacter* | 8 | **51** | *uncult_Gammaproteobacterium_JTB23* | 3 |
| **12** | *Fodinicurvata* | 2 | **52** | *uncult_Gammaproteobacterium_KI89A* | 2 |
| **13** | *Fulvivirga* | 2 | **53** | *uncult_Gammaproteobacterium_OM182* | 4 |
| **14** | *Halobacteriovorax* | 3 | **54** | *uncult_Ilumatobacteraceae* | 2 |
| **15** | *Hyphobacterium* | 11 | **55** | *uncult_Kiloniellaceae* | 5 |
| **16** | *Labrenzia* | 15 | **56** | *uncult_Leptospiraceae* | 2 |
| **17** | *Litorivivens* | 4 | **57** | *uncult_Magnetospiraceae* | 5 |
| **18** | *Maricaulis* | 2 | **58** | *uncult_Micavibrionaceae* | 3 |
| **19** | *Marinobacter* | 24 | **59** | *uncult_Microbacteriaceae* | 2 |
| **20** | *Marinoscillum* | 14 | **60** | *uncult_Myxococcales_P3OB-42* | 2 |
| **21** | *Mesorhizobium* | 2 | **61** | *uncult_Parvibaculaceae* | 2 |
| **22** | *Methyloceanibacter* | 2 | **62** | *uncult_Parvularculaceae* | 8 |
| **23** | *Mf105b01* | 3 | **63** | *uncult_Rhizobiaceae* | 4 |
| **24** | *Muricauda* | 2 | **64** | *uncult_Rhodobacteraceae* | 18 |
| **25** | *Owenweeksia* | 2 | **65** | *uncult_Rickettsiales_AB1* | 13 |
| **26** | *Pelagibius* | 2 | **66** | *uncult_Saccharospirillaceae* | 3 |
| **27** | *Peredibacter* | 4 | **67** | *uncult_Simkaniaceae* | 10 |
| **28** | *Pir4 lineage* | 2 | **68** | *uncult_Sphingobacteriales_NS11-12* | 2 |
| **29** | *Polycyclovorans* | 2 | **69** | *uncult_Terasakiellaceae* | 3 |
| **30** | *Pseudohongiella* | 5 | **70** | *Woeseia* | 3 |
| **31** | *Reichenbachiella* | 10 | | | |
| **32** | *Roseitalea* | 2 | | **All other genera** | **1** |
| **33** | *SM1A02* | 17 | | | |
| **34** | *Sva0996 marine group* | 2 | | | |
| **35** | *Tenacibaculum* | 2 | | | |
| **36** | *Tropicimonas* | 2 | | | |
| **37** | *Turneriella* | 4 | | | |
| **38** | *uncult_Bacteriovoracaceae* | 2 | | | |
| **39** | *uncult_Candidatus Kaiserbacteria* | 4 | | | |
| **40** | *uncult_Cellvibrionales* | 9 | | | |

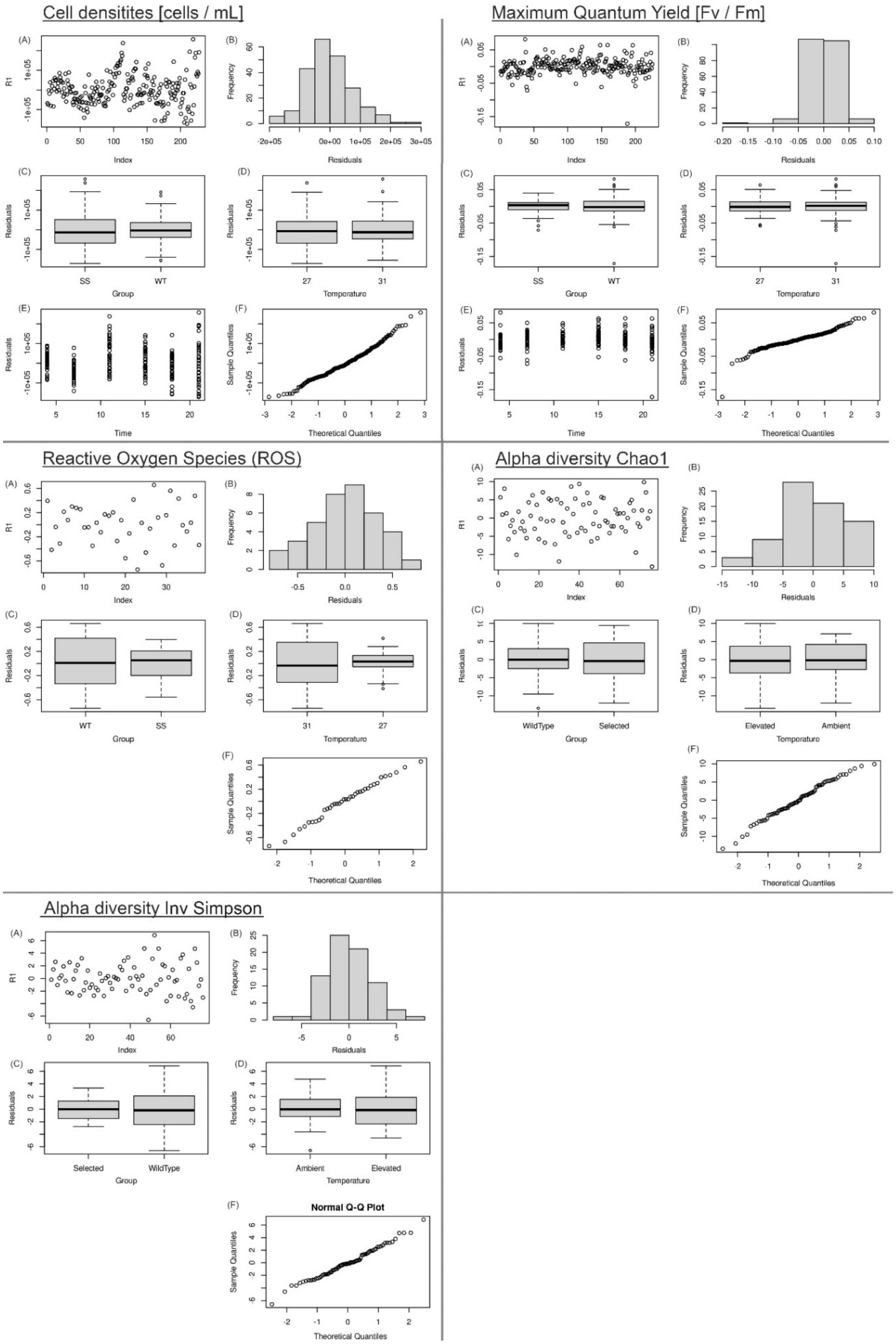


**Figure S1 Verification of generalized linear mixed effect model assumptions.** For each model the following plots are shown: (A) Fitted values versus residuals to test for homogeneity of variances, (B) histogram of the residuals to check for normal distribution of the data, (C), (D) and (E) residuals of the respective factors to check for data independence, (F) residuals Q-Q plot to check for normal distribution of the data.

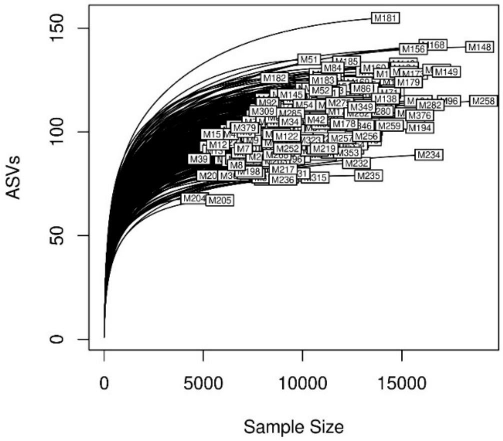


**Figure S2. Rarefaction curves of all samples.** All samples have reached asymptote as an indication that all diversity is covered with the amount of sequencing per sample.

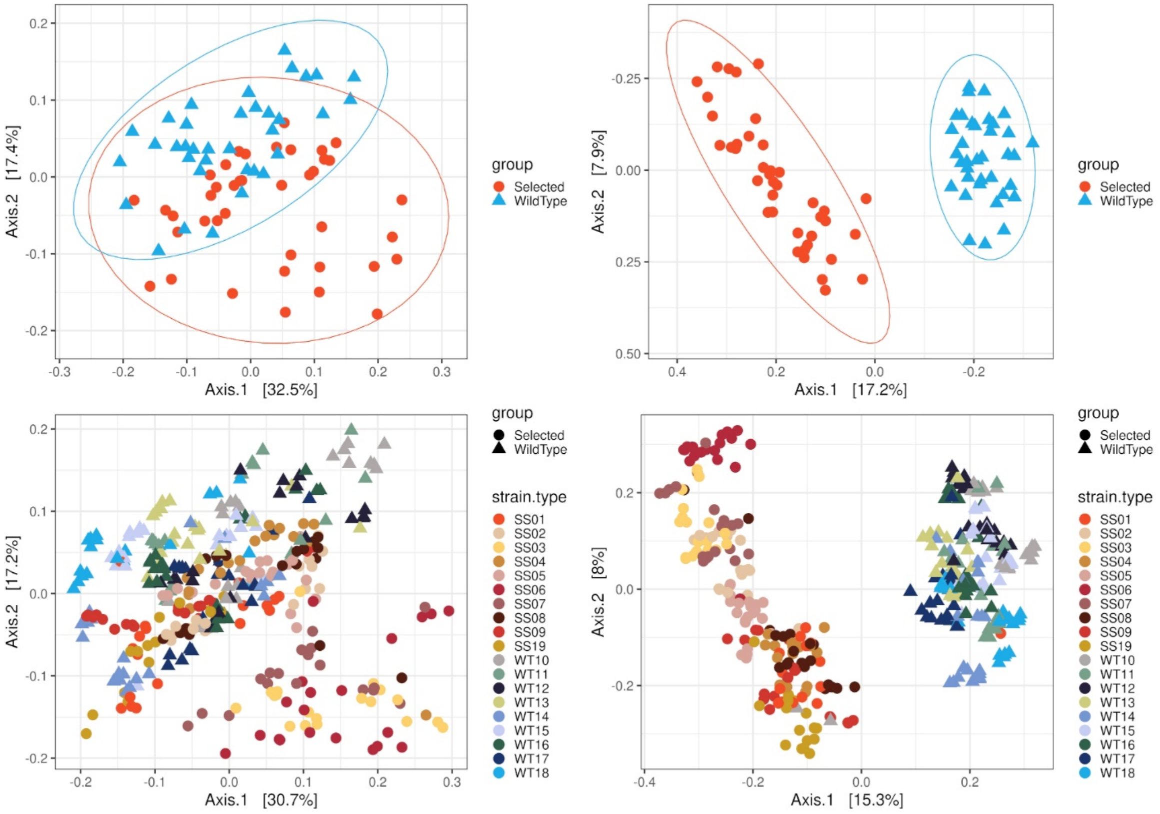


**Figure S3. PCoA visualization using weighted UniFrac (left panels) and Jaccard distances (right panels)** separating samples according to the microalgae group (SS or WT) based on a reduced dataset with average values across strains. On the bottom, the individual *Cladocopium* C1$^{acro}$ strains are shown according to the full dataset (left weighted UniFrac, right Jaccard).

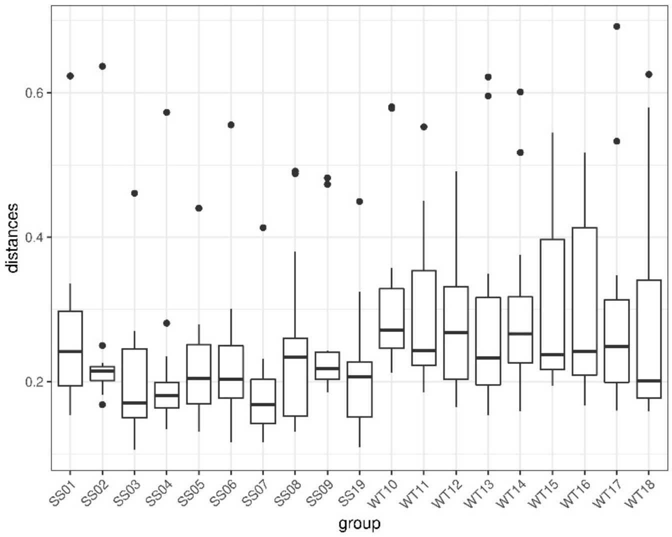


**Figure S4 Distance to centroids for individual strains calculated from PCoA on beta-diversity Bray-Curtis distance matrix.** The distance to the centroids of the heat-evolved strains (dispersal) is smaller compared to the wild-type strains. The smaller dispersal of the heat-evolved strains indicates that their beta-diversity composition is more similar between samples, while beta diversity has a larger spread for the samples of the wild-type strains.

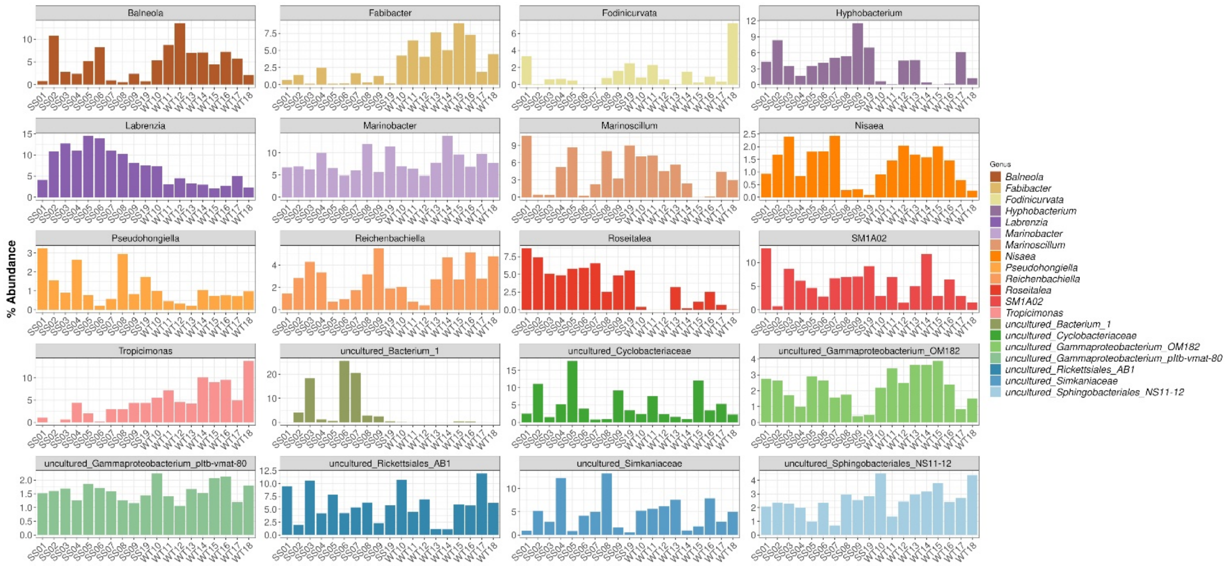


**Figure S5 Barplot showing respective samples strains with the top 20 bacteria of highest abundances in % displayed at the genus level.** Genera are color coded. Values for the strains are averaged across the two time-points and the two treatments.

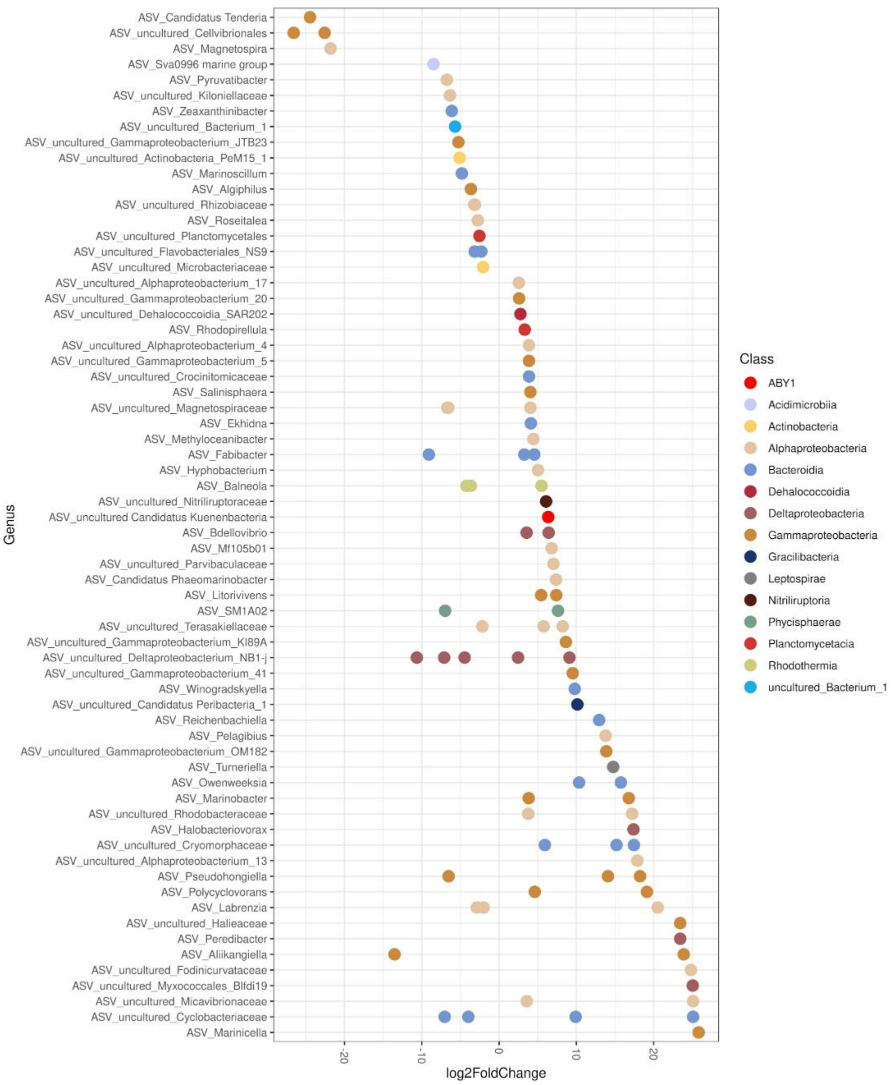


**Figure S6 DESeq2 differential abundance analysis with contrast adjustment to view WT and SS strains.** Significance level $p < 0.001$. Table with all ASVs and numeric log-fold changes is available in the supplementary data materials. Each dot in the plot represents one ASV with the respective genus an on y-axis and the colors as the family. Positive values indicate that an ASV is enriched in WT samples. Negative values indicate that an ASV is enriched in SS samples (SS+, and SS-).

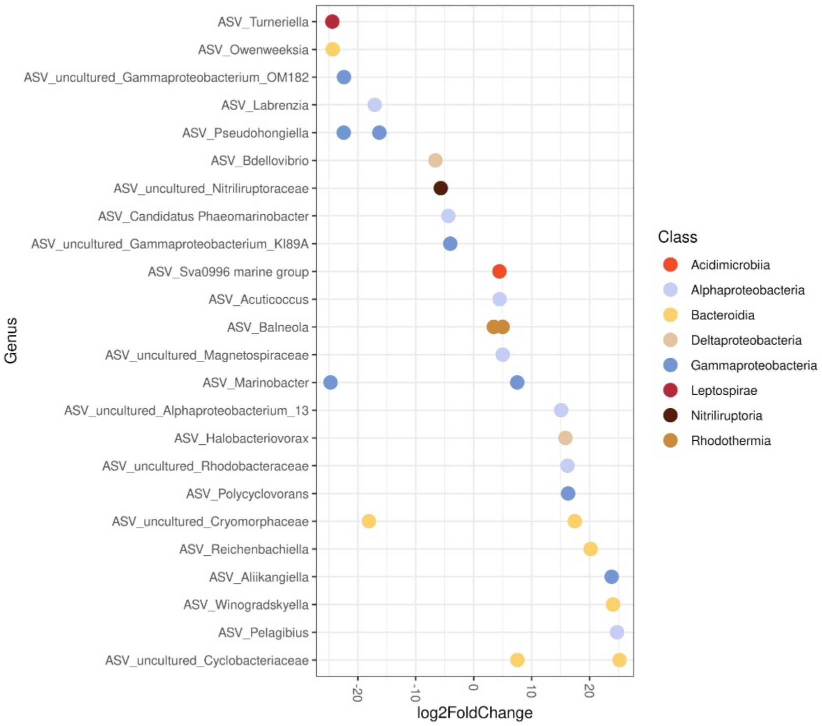


**Figure S7 DESeq2 differential abundance analysis with contrast adjustment to view SS conferring vs SS non-conferring strains.** Significance level $p < 0.001$. Table with all ASVs and numeric log-fold changes is available in the supplementary data materials. Each dot in the plot represents one ASV with the respective genus an on y-axis and the colors as the family. ASVs that have a positive log-fold change are enriched in SS- samples. ASVs that have a negative log-fold change are enriched in SS+ samples.
