# Supplementary material for: Long-Term Heat Selection of the Coral Endosymbiont Cladocopium C1acro (Symbiodiniaceae) Stabilizes Associated Bacterial Communities
Source: Int J Mol Sci. 2022 Apr 28;23(9):4913. doi: 10.3390/ijms23094913 (PMC9101544; doi:10.3390/ijms23094913)
Supplement: Supplementary file 1 [file ijms-23-04913-s001.zip › R_CODE_and_DATA_FILES.zip!/SupplementaryMaterials_R_notebook.nb.html]

R Notebook: Long-term heat selection of the coral endosymbiont Cladocopium C1 acro (Symbiodiniaceae) stabilizes associated bacterial communities


Code 

- Show All Code
- Hide All Code
- Download Rmd

# R Notebook: Long-term heat selection of the coral endosymbiont Cladocopium C1 acro (Symbiodiniaceae) stabilizes associated bacterial communities


```
## MANUSCRIPT AUTHORS:
# Patrick Buerger 1,2,* , Ruby Vanstone 2,* , Justin Maire 2 , Madeleine JH van Oppen 2,3

# Affiliations:
# 1 Applied Biosciences, Macquarie University, North Ryde, NSW 2113
# 2 School of BioSciences, The University of Melbourne, Parkville, VIC 3010
# 3 Australian Institute of Marine Science, PMB #3, Townsville, QLD 4810
# * both authors contributed equally to the manuscript.
```


```
# DATA ANALYSIS IN DISCUSSION WITH KAT DAMJANOVIC
# ANY QUESTIONS REGARDING THE CODE: patrick.buerger@mq.edu.au 

# CODE NEEDED A CLEAN UP - BUT THERE WASNT ENOUGH TIME BEFORE PUBLICATION :/
```


```
################################ SET UP WORKSPACE ###########################

#load libraries 
library(ape)
#BiocManager::install("phyloseq")
library(phyloseq)
library(magrittr)
library(ggplot2)
library(plyr)
library(dplyr)
library(tidyverse)
library(vegan)
#install.packages("indicspecies")
library(indicspecies)
#if (!requireNamespace("BiocManager", quietly = TRUE))
#    install.packages("BiocManager")
#BiocManager::install("DESeq2")
library(DESeq2)
library(RColorBrewer)
#install.packages("gapminder")
library(gapminder)
#install.packages("installr")
library(installr)
#BiocManager::install("mixOmics")
library(mixOmics)
#install.packages("RVAideMemoire")
library(RVAideMemoire)
library(rgl)
library(openxlsx)
#install.packages("sfsmisc")
library(sfsmisc)
library(ggpubr)
library(Hmisc)
#install.packages("Rcpp")
library(Rcpp)
library(car)
library(pairwiseAdonis)
#install.packages("gridExtra")
library(gridExtra)
#install.packages("wesanderson")
library(wesanderson)
library(RColorBrewer)
library(cowplot)
library(tidyr)
library(rstatix)
#install.packages("emmeans")
library(emmeans)
#attach(mtcars)

#BiocManager::install("phyloseq", force = TRUE)
library(phyloseq)

# SOME PACKAGES CLASH WITH DEPENDENCIES SUCH AS 
#BiocManager::install("microbiome")
library(microbiome)
```


```
############################ IMPORT DATA ##########################
############################ IMPORT DATA ##########################
############################ IMPORT DATA ##########################


# Read in OTU table
otuData <- read.table(
  "table.tsv",
  header = TRUE,
  sep = "\t",
  row.names = 1
)


# Read in taxonomy table
taxData <- read.table(
  "taxonomy.tsv",
  sep = "\t",
  fill = TRUE,
  row.names = 1,
  header = FALSE
)

# Read in metadata file
metaData2 <- import_qiime_sample_data('metadata.txt')

# Add levels of taxonomy to taxonomy table
colnames(taxData3) <- c("Domain", "Phylum", "Class", "Order", "Family", "Genus", "Species")

# Convert ASV and taxonomy tables for phyloseq compatibility
otuDataMat <- as.matrix(otuData)
taxDataMat <- as.matrix(taxData3)

# Import tree file as a phylosq object
tree <- read_tree("tree.nwk")

# Combine ASVs, taxonomy, and metadata files into a phyloseq object
phy <- phyloseq(
  otu_table(
    otuDataMat, 
    taxa_are_rows = T
  ),
  tax_table(taxDataMat),
  sample_data(metaData2)
)

# CONVERT characters into factors
sample_data(phy)$group <- as.factor(sample_data(phy)$group)
sample_data(phy)$strain.type <- as.factor(sample_data(phy)$strain.type)
sample_data(phy)$heat.tolerance <- as.factor(sample_data(phy)$heat.tolerance)
sample_data(phy)$experiment.stage <- as.factor(sample_data(phy)$experiment.stage)
sample_data(phy)$sampleID <- as.factor(sample_data(phy)$sampleID)
sample_data(phy)$temperature <- as.factor(sample_data(phy)$temperature)
sample_data(phy)$Type <- as.factor(sample_data(phy)$Type)
sample_data(phy)$replicate.number <- as.factor(sample_data(phy)$replicate.number)
sample_data(phy)$extraction.date <- as.factor(sample_data(phy)$extraction.date)
sample_data(phy)$time <- as.factor(sample_data(phy)$time)

# Merge the phyloseq objects into a single object
phy <- merge_phyloseq(phy, tree)

# Delete the matrices as they are not used in further analyses
rm(otuDataMat)
rm(taxDataMat)
```


```
############# Filtering of unwanted taxa and samples and rarefaction ###########
############# Filtering of unwanted taxa and samples and rarefaction ###########
############# Filtering of unwanted taxa and samples and rarefaction ###########


# Remove chloroplast and mitochondria
class2 <- phy %>%
  subset_taxa(
    Domain != "Bacteria" &
    Family  != "mitochondria" &
    Class   != "Chloroplast"
  )

# Remove unneeded samples (Mocks)
class3 <- subset_samples(class2, !(sampleID %in% c("Mock1", "Mock2", "Mock3")))

# Remove those ASVs that were not observed in any of the current samples
sum(taxa_sums(class3) == 0)
class30 <- class3
class3 <- prune_taxa(taxa_sums(class30) > 0, class3)

# Remove low-abundance samples
minTotRelAbun <- 1e-5
x <- taxa_sums(class3)
keepTaxa <- names(which((x / sum(x)) > minTotRelAbun))
class4 <- prune_taxa(keepTaxa, class3)

# clean up older files
rm(class30,class3,class2,x,minTotRelAbun,keepTaxa)
```


```
############## Identify contaminant sequences and remove them ################
############## Identify contaminant sequences and remove them ################
############## Identify contaminant sequences and remove them ################


# Finding taxa only present in the negative controls (the variable "Type" includes Coral, Water and NegativeCtrl, which are the blanks and no template PCRs:
class4.ngtv.ctrl <- subset_samples(class4, Type %in% c("NegCtrl"))

# From the dataset, remove taxa that are absent from the negative controls:
sum(taxa_sums(class4.ngtv.ctrl) == 0)
class4.ngtv.ctrl0 <- class4.ngtv.ctrl
class4.ngtv.ctrl <- prune_taxa(taxa_sums(class4.ngtv.ctrl0) > 0, class4.ngtv.ctrl)

# Calculating the relative abundance for all taxa in the complete dataset:
percent <- class4 %>%
  transform_sample_counts(function(x) {(x/sum(x))} ) 

# Select only the taxa present in the negative controls. Subsetting is based on the ASV identifier (row names)
percent.ngtv <- subset_taxa(percent, rownames(tax_table(percent)) %in% rownames(tax_table(class4.ngtv.ctrl)))

# Create these variables to export them as .csv files
output <- otu_table(percent.ngtv)
output2 <- tax_table(percent.ngtv)

# Write the list of the "negative control taxa" in a spreadsheet together with their abundances across all samples.
# I then created a new .xlsx sheet combining the abundance and taxonomic information.

### CONTAMINANTS TABLE, NEED TO DO EXTRA CALCULATIONS
write.table(output, file = "./Abundance_Blanks_16s_RelativeAbundance.csv", sep = ",")
write.table(output2, file = "./Abundance_Blanks_Taxa_16s_Taxa.csv", sep = ",")

# For example, ASVs that were identified as contaminants:
Marinobacter5 <- "06b5e1454ef185b5bc7f4ca47bd992b2"
Deltaproteobacteria <- "11c85c81bbf92551edab8ebebd5393e9"
Micrococcus <- "11e86a2321423aa8ab1507219e5dfa7b"
Pelomonas <- "1984c61c1eef5052f94a4ab69861ff01"
Saccharospirillaceae <- "2322789c09137ff7cddd240c82d6617c"
Micavibrionales <- "233f6c46c0a8d4b6bc76d92bac5018b4"
Leptospiraceae <- "2f26574604ac929004ba032a195a8ac2"
Oceanococcus <- "3dbab6fb4a538cd9806907048ce57f2c"
Marinoscillum2 <- "41a0864e669378c2a8c418c3d4665c7d"
Cyclobacteriaceae <- "50e87b427fbb4efb0166c496f54293ea"
Ochrobactrum <- "52a861498f6e7c09ea26b8fcf39a24ee"
Gammaproteobacteria <- "57bcd87cc81b962012eed4c67f78e5d5"
Cyclobacteriaceae1 <- "587b8d1cfb8f222bce211a94e0f6b4ea"
Flavobacteriales <- "68a643eed6cab3f919613505d7d0f99d"
Staphylococcus <- "7880fa1f62e6ea6e76f2715a469f43ba"
Parvularculaceae <- "7ebfed33fea995543992c368e5c97581"
Marinobacter <- "8131284d353c546e95b65b9e8fd626b0"
Marinobacter4 <- "a231a2d7b5762dc39583d37e605b3f6a"
Marinoscillum <- "a461aa9654c4914899df4e79e93fdec1"
OM182 <- "add36f351a8ccd93f646773da3aaebd5"
Labrenzia <- "bef1168257d4c3a8852faf149ab41337"
Marinobacter3 <- "c7ecf960ce6b5cf13a55160c4158e3a1"
Bermanellamarisrubri <- "c9b7375784a459212fb31444b7401c22"
Roseivirga <- "e112b1eb495e0a736adb0bd97a6d40ca"
OM1822 <- "d1c91c063173b3d53df0957d1fb39d71"
Micavibrionaceae <- "d2603537da3f30686b576ed40c77dea5"
Rhizobiaceae <- "f4b66f71f79d3d0ff34a3d9cb57223c6"
Kiloniellaceae <- "fa0ada565de08155b5ef1f1d3c0b6753"
Alishewanella <- "df683c9c7758203a0867a3109ab54097"
Staphylococcus2 <- "4098046809096a16b10b23c9614d2db4"

# We can put them in a list in order to remove them from the dataset
contamination <- c(Marinobacter5, Deltaproteobacteria, Micrococcus, Pelomonas, Saccharospirillaceae, Micavibrionales, Leptospiraceae, Oceanococcus, Marinoscillum2, Cyclobacteriaceae, Ochrobactrum, Gammaproteobacteria, Cyclobacteriaceae1, Flavobacteriales, Staphylococcus, Parvularculaceae, Marinobacter, Marinobacter4, Marinoscillum, OM182, Labrenzia, Marinobacter3, Bermanellamarisrubri, Roseivirga, OM1822, Micavibrionaceae, Rhizobiaceae, Kiloniellaceae, Alishewanella, Staphylococcus2)

# Removing contaminants from the dataset
class4.clean <- subset_taxa(class4, !(rownames(tax_table(class4)) %in% contamination))

# Clean Workspace
rm(contamination, class4.ngtv.ctrl0, class4.ngtv.ctrl, percent.ngtv, percent, output, output2)

rm(Marinobacter5, Deltaproteobacteria, Micrococcus, Pelomonas, Saccharospirillaceae, Micavibrionales, Leptospiraceae, Oceanococcus, Marinoscillum2, Cyclobacteriaceae, Ochrobactrum, Gammaproteobacteria, Cyclobacteriaceae1, Flavobacteriales, Staphylococcus, Parvularculaceae, Marinobacter, Marinobacter4, Marinoscillum, OM182, Labrenzia, Marinobacter3, Bermanellamarisrubri, Roseivirga, OM1822, Micavibrionaceae, Rhizobiaceae, Kiloniellaceae, Alishewanella, Staphylococcus2)


#############################
#### TOTAL taxa in data
#1359 ASVs in dataset

#### Remove taxa with mitochondria and chloroplast
# 0

#### Remove taxa that have 0 across all samples: 24
# Taxa left: 1335

#### remove low abundant taxa with minTotRelAbun <- 1e-5
# Taxa left: 518

#### remove contaminants that are more than 40% abundant in blanks
# Taxa left after clean in 'class4.clean': 488


# RENAME SOME OF THE ENTRIES
sample_data(class4.clean)$heat.tolerance <- revalue(sample_data(class4.clean)$heat.tolerance, c("SS_conferring"="SS+", "SS_non-conferring"="SS-","Wild-type"="WT"))

class4.clean.sub <- subset_samples(class4.clean, heat.tolerance %in% c("SS+", "SS-", "WT"))


# CREATE NEW VARIABLES $Group1
variable1 = as.character(get_variable(class4.clean.sub, "group"))
variable3 = as.character(get_variable(class4.clean.sub, "experiment.stage"))
sample_data(class4.clean.sub)$Group1 <- mapply(paste0, variable1, sep = ".", variable3)
#merge_samples(class4.clean.sub, "Group1")
#
sample_data(class4.clean.sub)$Group1 <- as.factor(sample_data(class4.clean.sub)$Group1)
#
sample_variables(class4.clean.sub)


# CREATE NEW VARIABLES $Group2
variable1 = as.character(get_variable(class4.clean.sub, "group"))
variable3 = as.character(get_variable(class4.clean.sub, "temperature"))
sample_data(class4.clean.sub)$Group2 <- mapply(paste0, variable1, sep = ".", variable3)
#merge_samples(class4.clean.sub, "Group2")
#
sample_data(class4.clean.sub)$Group2 <- as.factor(sample_data(class4.clean.sub)$Group2)
#
sample_variables(class4.clean.sub)


# CREATE NEW VARIABLES $Group3
variable1 = as.character(get_variable(class4.clean.sub, "group"))
variable2 = as.character(get_variable(class4.clean.sub, "temperature"))
variable3 = as.character(get_variable(class4.clean.sub, "experiment.stage"))
sample_data(class4.clean.sub)$Group3 <- mapply(paste0, variable1, sep = ".", variable2, sep = ".", variable3, collapse = "_")
#merge_samples(class4.clean.sub, "Merge")
#
sample_data(class4.clean.sub)$Group3 <- as.factor(sample_data(class4.clean.sub)$Group3)
#
sample_variables(class4.clean.sub)
#
sample_data(class4.clean.sub)$Group3 <- revalue(sample_data(class4.clean.sub)$Group3, c("Selected.Ambient.after"="SS.Ambient.After", "Selected.Ambient.before"="SS.Ambient.Before","Selected.Elevated.after"="SS.Elevated.After", "Selected.Elevated.before"="SS.Elevated.Before","WildType.Ambient.after"="WT.Ambient.After", "WildType.Ambient.before"="WT.Ambient.Before","WildType.Elevated.after"="WT.Elevated.After", "WildType.Elevated.before"="WT.Elevated.Before"))


# CREATE NEW VARIABLES $Group4
variable2 = as.character(get_variable(class4.clean.sub, "temperature"))
variable3 = as.character(get_variable(class4.clean.sub, "experiment.stage"))
sample_data(class4.clean.sub)$Group4 <- mapply(paste0, variable2, sep = ".", variable3, 
    collapse = "_")
#merge_samples(class4.clean.sub, "Merge")
#
sample_data(class4.clean.sub)$Group4 <- as.factor(sample_data(class4.clean.sub)$Group4)
#
sample_variables(class4.clean.sub)


# CREATE NEW VARIABLES $MERGE
variable1 = as.character(get_variable(class4.clean.sub, "strain.type"))
variable2 = as.character(get_variable(class4.clean.sub, "temperature"))
variable3 = as.character(get_variable(class4.clean.sub, "experiment.stage"))
sample_data(class4.clean.sub)$Merge1 <- mapply(paste0, variable1, sep = ".", variable2, sep = ".", variable3, 
    collapse = "_")
#merge_samples(class4.clean.sub, "Merge")
#
sample_data(class4.clean.sub)$Merge1 <- as.factor(sample_data(class4.clean.sub)$Merge1)
#
sample_variables(class4.clean.sub)
```


```
############## CREATE RAREFACTION CURVES ################
############## CREATE RAREFACTION CURVES ################
############## CREATE RAREFACTION CURVES ################
# https://micca.readthedocs.io/en/latest/phyloseq.html

# CHECK THAT SEQUENCED DEEP ENOUGH
# CHECK THAT SEQUENCED DEEP ENOUGH
# CHECK THAT SEQUENCED DEEP ENOUGH


sort(sample_sums(class4.clean.sub))
# THE CODE WILL ALSO CREATE A RAREFIED DATA SET "coralFR"
# Check here for lowest number for sample.size number in following code
set.seed(2)
coralFR = rarefy_even_depth(class4.clean.sub, sample.size = 4562, rngseed = 2)

time<-format(Sys.time(),"%d_%m_%Y")
Title <- paste("RareFaction")
FileName <- paste(Title,time,".jpg")
jpeg(file=FileName, width = 5000, height = 2500, res = 500)
par(mfrow=c(1,2))
rarecurve(t(otu_table(class4.clean.sub)), step=50, cex=0.5, ylab = "ASVs")
rarecurve(t(otu_table(coralFR)), step=50, cex=0.5, ylab = "ASVs")
dev.off()


# class4.clean.sub = FINAL DATA
# coralFR = Rarefied FINAL DATA #-> NOT USED HERE
```


```
############## AVOID PSEUDOREPLICATION ################
############## AVOID PSEUDOREPLICATION ################
############## AVOID PSEUDOREPLICATION ################

###############################
###############################
###############################
# TO AVOID PSEUDOREPLICATION: MERGE Strain replicates to one (here sum).
# READ THIS: https://github.com/joey711/phyloseq/issues/465
# REQUIRED FOR ALPHA DIVERSITY STATS AND BETA DIVERSITY
###############################
sample_variables(class4.clean.sub)

# Merge according to new variable, reduces sample size from 368 to 76 (due to approximately 5 replicates per sample)
merged_data <- merge_samples(class4.clean.sub, "Merge1")
sample_data(merged_data)$Merge1 <- levels(sample_data(class4.clean.sub)$Merge1)
sample_data(merged_data)$Merge1 <- as.factor(sample_data(merged_data)$Merge1)


# MAKE ALL OTHERS A FACTOR
sample_data(merged_data)$temperature <- as.factor(sample_data(merged_data)$temperature)
sample_data(merged_data)$group <- as.factor(sample_data(merged_data)$group)
sample_data(merged_data)$experiment.stage <- as.factor(sample_data(merged_data)$experiment.stage)
sample_data(merged_data)$strain.type <- as.factor(sample_data(merged_data)$strain.type)
sample_data(merged_data)$heat.tolerance <- as.factor(sample_data(merged_data)$heat.tolerance)
sample_data(merged_data)$Group1 <- as.factor(sample_data(merged_data)$Group1)
sample_data(merged_data)$Group2 <- as.factor(sample_data(merged_data)$Group2)
sample_data(merged_data)$Group3 <- as.factor(sample_data(merged_data)$Group3)
sample_data(merged_data)$Group4 <- as.factor(sample_data(merged_data)$Group4)
sample_data(merged_data)$Merge1 <- as.factor(sample_data(merged_data)$Merge1)
sample_data(merged_data)$sampleID <- as.factor(sample_data(merged_data)$sampleID)
sample_data(merged_data)$replicate.number <- as.factor(sample_data(merged_data)$replicate.number)
sample_data(merged_data)$extraction.date <- as.factor(sample_data(merged_data)$extraction.date)
sample_data(merged_data)$Type <- as.factor(sample_data(merged_data)$Type)
sample_data(merged_data)$time <- as.factor(sample_data(merged_data)$time)


# RENAME FACTORS
sample_data(merged_data)$temperature <- revalue(sample_data(merged_data)$temperature, c("1"="Ambient", "2"="Elevated"))
sample_data(merged_data)$group <- revalue(sample_data(merged_data)$group, c("1"="Selected", "2"="WildType"))
sample_data(merged_data)$experiment.stage <- revalue(sample_data(merged_data)$experiment.stage, c("1"="day 18", "2"="day 2"))
sample_data(merged_data)$strain.type <- revalue(sample_data(merged_data)$strain.type, c("1"="SS1", "2"="SS2", "3"="SS3", "4"="SS4", "5"="SS5", "6"="SS6", "7"="SS7", "8"="SS8", "9"="SS9", "10"="SS19", "11"="WT10", "12"="WT11", "13"="WT12", "14"="WT13", "15"="WT14", "16"="WT15", "17"="WT16", "18"="WT17", "19"="WT18"))
sample_data(merged_data)$heat.tolerance <- revalue(sample_data(merged_data)$heat.tolerance, c("1"="SS_plus", "2"="SS_minus","3"="WT"))
sample_data(merged_data)$Group1 <- revalue(sample_data(merged_data)$Group1, c("1"="SS before", "2"="SS after","3"="WT before", "4"="WT after"))
sample_data(merged_data)$Group2 <- revalue(sample_data(merged_data)$Group2, c("1"="SS ambient", "2"="SS elevated","3"="WT ambient", "4"="WT elevated"))
sample_data(merged_data)$Group3 <- revalue(sample_data(merged_data)$Group3, c("1"="SS.Ambient.After", "2"="SS.Ambient.Before","3"="SS.Elevated.After", "4"="SS.Elevated.Before","5"="WT.Ambient.After", "6"="WT.Ambient.Before","7"="WT.Elevated.After", "8"="WT.Elevated.Before"))
sample_data(merged_data)$Group4 <- revalue(sample_data(merged_data)$Group4, c("1"="Ambient.After", "2"="Ambient.Before","3"="Elevated.After", "4"="Elevated.Before"))


# ALSO REORDER FACTORS
sample_data(merged_data)$Group1 <- factor(sample_data(merged_data)$Group1, levels=c("SS before", "SS after", "WT before", "WT after"))
sample_data(merged_data)$Group3 <- factor(sample_data(merged_data)$Group3, levels=c("SS.Ambient.Before","SS.Ambient.After","SS.Elevated.Before","SS.Elevated.After","WT.Ambient.Before","WT.Ambient.After","WT.Elevated.Before","WT.Elevated.After"))
sample_data(merged_data)$Group4 <- factor(sample_data(merged_data)$Group4, levels=c("Ambient.Before", "Ambient.After", "Elevated.Before", "Elevated.After"))


#### merged_data = FINAL DATA SET FOR STATS
```


```
################# Alpha Diversity STATISTICS ############
################# Alpha Diversity STATISTICS ############
################# Alpha Diversity STATISTICS ############


# FOR ALPHA DIVERSITY: FIRST DO CALCULATE ALPHA DIVERSITY INDECES ON FULL DATA, THEN MERGE THE INDECES, see link. If OTU table is merged prior to calculating indices, it will get rid of small low abundance taxa.
# https://github.com/joey711/phyloseq/issues/465

# Estimate Richness on DATA
rich2 <- estimate_richness(class4.clean.sub)
sampleID <- rownames(rich2)
rich2 <- cbind(sampleID, rich2) %>% as.data.frame()

# Joining the metadata of the samples to the diversity indices
rich_fac2 <- rich2 %>% left_join(data.frame(sample_data(class4.clean.sub)))

# WRTIE DATA FILE
##### WRITE FILES ######
#write.csv(rich_fac2,"./stats_AlphaDiv_rich_fac_updated_MERGED_DATA.csv", row.names = FALSE)

# THEN MERGE SAMPLES ACCORDING TO COLUMN "MERGE"
aggregate1 <- aggregate(cbind(Chao1, InvSimpson) ~ Merge1, rich_fac2, mean)
rich_fac2 <- aggregate1 %>% left_join(data.frame(sample_data(merged_data)))


# RENAME FACTOR LEVELS
levels(rich_fac2$heat.tolerance) <- sub("SS_plus", "SS+", levels(rich_fac2$heat.tolerance))
levels(rich_fac2$heat.tolerance) <- sub("SS_minus", "SS-", levels(rich_fac2$heat.tolerance))


# REORDER FACTORS
rich_fac2$strain.type <- factor(rich_fac2$strain.type , levels=c("SS1","SS2","SS3","SS4","SS5","SS6","SS7","SS8","SS9","SS19","WT10","WT11","WT12","WT13","WT14","WT15","WT16","WT17","WT18"))
#
rich_fac2$heat.tolerance <- factor(rich_fac2$heat.tolerance , levels=c("SS+", "SS-","WT"))
#
rich_fac2$experiment.stage <- factor(rich_fac2$experiment.stage , levels=c("day 2", "day 18"))


#
Title <- paste("./ALPHA")
FileName <- paste(Title,time,".txt")
write.csv(rich_fac2,file = "rich_fac2.NEW_2022_2.csv")


###################################################
### LINEAR MIXED EFFECTS MODEL, ALPHA DIVERSITY ###
###################################################

# LOAD PACKAGE
library(plyr)
library(nlme)

# MAKE BACKUPENTRY
rich_fac3 <- rich_fac2

# MAKE experiment.stage numeric 
rich_fac3$experiment.stage <- revalue(sample_data(rich_fac3)$experiment.stage, c("before"="1", "after"="2")) 
rich_fac3$experiment.stage <- as.numeric(rich_fac3$experiment.stage)
str(rich_fac3)  


#### CHAO 1 #### CHAO 1 #### CHAO 1
#### CHAO 1 #### CHAO 1 #### CHAO 1
#### CHAO 1 #### CHAO 1 #### CHAO 1

# TRY DIFFERENT MODELS AND COMPARE FIT
Chao1.lme <- lme(Chao1 ~ group*temperature*experiment.stage, random = ~ 1|strain.type, data = rich_fac3, method="ML")
Chao2.lme <- lme(Chao1 ~ group*temperature*experiment.stage, random = ~ 1|experiment.stage, data = rich_fac3, method="ML")
anova(Chao1.lme,Chao2.lme)


Chao1.1.lme <- lme(Chao1 ~ group*temperature*experiment.stage, random = ~ 1|strain.type, data = rich_fac3)
LMEM.chao1 <- anova(Chao1.1.lme)
anova(Chao1.1.lme)
summary(Chao1.1.lme)
#

Title <- paste("./STATS_LMEM_2022_chao1_noPseudo")
FileName <- paste(Title,time,".txt")
write.table(LMEM.chao1, file = FileName, sep = "\t")


Chao1.1.lme <- lme(Chao1 ~ group*temperature*experiment.stage, random = ~ 1|strain.type,data = rich_fac3, control=list(msMaxIter=100,opt= "optim",msVerbose=TRUE), method="REML")
anova(Chao1.1.lme)
summary(Chao1.1.lme)


res_lme=residuals(Chao1.1.lme)
plot(res_lme)
qqnorm(res_lme)

plot(allEffects(Chao1.1.lme))


########## model VALIDATION 
time<-format(Sys.time(),"%d_%m_%Y")
Title <- paste("LMEM_Chao1_model_assumptions")
FileName <- paste(Title,time,".jpg")
#jpeg(file=FileName, width = 2220, height = 1280, res = 200)
jpeg(file=FileName, width = 2500, height = 3000, res = 400)
par(mfrow = c(3,2))
R1 <- resid(Chao1.1.lme)
plot(R1)
hist(R1, xlab = "Residuals", main = "")
plot(rich_fac3$group, R1, xlab = "Group", ylab = "Residuals")
plot(rich_fac3$temperature, R1, xlab = "Temperature", ylab = "Residuals")
#plot(rich_fac3$experiment.stage, R1, xlab = "experiment.stage", ylab = "Residuals")
qqnorm(R1)
dev.off() 


# REORDER CONTRASTS, THEN REFIT MODEL TO GET COMPARISONS. ALTERNATIVELY USE PACKAGE EMMEANS 
# https://cran.r-project.org/web/packages/emmeans/index.html
rich_fac3$temperature<-relevel(rich_fac3$temperature,"Elevated")
rich_fac3$group<-relevel(rich_fac3$group,"WildType")
rich_fac3$Group.Temp = with(rich_fac3, interaction(group,temperature))
#
rich_fac3.1.lme <- lme(Chao1 ~ experiment.stage*Group.Temp, random = ~ 1|strain.type, data = rich_fac3,control=list(msMaxIter=100,opt= "optim",msVerbose=TRUE), method="REML")
summary(rich_fac3.1.lme)
# CHECK OUTPUT IN R AT THE END OF THE CHUNK

# NOT ALL INFORMATION GETS SAVED WITH THE FOLLOWING CODE
Title <- paste("./STATS_LMEM_2022_CHAO1_noPseudo_RELEVEL_WT31")
FileName <- paste(Title,time,".txt")
capture.output(summary(rich_fac3.1.lme),file=FileName)


#### InvSimpson #### InvSimpson #### InvSimpson
#### InvSimpson #### InvSimpson #### InvSimpson
#### InvSimpson #### InvSimpson #### InvSimpson
InvSimpson.1.lme <- lme(InvSimpson ~ group*temperature*experiment.stage, random = ~ 1|strain.type, data = rich_fac3, method="ML")
InvSimpson.2.lme <- lme(InvSimpson ~ group*temperature*experiment.stage, random = ~ 1|experiment.stage, data = rich_fac3, method="ML")
anova(InvSimpson.1.lme,InvSimpson.2.lme)


InvSimpson.1.1.lme <- lme(InvSimpson ~ group*temperature*experiment.stage, random = ~ 1|strain.type, data = rich_fac3)
anova(InvSimpson.1.1.lme)
summary(InvSimpson.1.1.lme)

library(tidyverse)

InvSimpson.1.1.lme <- lme(InvSimpson ~ group*temperature*experiment.stage, random = ~ 1|strain.type, data = rich_fac3, control=list(msMaxIter=100,opt= "optim",msVerbose=TRUE), method="REML")
LMEM.InvSimpson.1.1.lme <- anova(InvSimpson.1.1.lme)
#
Title <- paste("./STATS_LMEM_2022_InvSimpson_noPseudo")
FileName <- paste(Title,time,".txt")
write.table(LMEM.InvSimpson.1.1.lme, file = FileName, sep = "\t")


summary(InvSimpson.1.1.lme)


res_lme=residuals(InvSimpson.1.1.lme)
plot(res_lme)
qqnorm(res_lme)

plot(allEffects(InvSimpson.1.1.lme))


########## model VALIDATION 
time<-format(Sys.time(),"%d_%m_%Y")
Title <- paste("LMEM_InvSimpson_model_assumptions")
FileName <- paste(Title,time,".jpg")
#jpeg(file=FileName, width = 2220, height = 1280, res = 200)
jpeg(file=FileName, width = 2500, height = 3000, res = 400)
par(mfrow = c(3,2))
R1 <- resid(InvSimpson.1.1.lme)
plot(R1)
hist(R1, xlab = "Residuals", main = "")
plot(rich_fac3$group, R1, xlab = "Group", ylab = "Residuals")
plot(rich_fac3$temperature, R1, xlab = "Temperature", ylab = "Residuals")
#plot(rich_fac3$experiment.stage, R1, xlab = "experiment.stage", ylab = "Residuals")
qqnorm(R1)
dev.off() 


# REORDER CONTRASTS, THEN REFIT MODEL TO GET COMPARISONS. ALTERNATIVELY USE PACKAGE EMMEANS 
# https://cran.r-project.org/web/packages/emmeans/index.html
rich_fac3$temperature<-relevel(rich_fac3$temperature,"Elevated")
rich_fac3$group<-relevel(rich_fac3$group,"WildType")
rich_fac3$Group.Temp = with(rich_fac3, interaction(group,temperature))
#
rich_fac3.1.lme <- lme(InvSimpson ~ experiment.stage*Group.Temp, random = ~ 1|strain.type, data = rich_fac3,control=list(msMaxIter=100,opt= "optim",msVerbose=TRUE), method="REML")
summary(rich_fac3.1.lme)
# CHECK OUTPUT IN R AT THE END OF THE CHUNK

# NOT ALL INFORMATION GETS SAVED WITH THE FOLLOWING CODE
Title <- paste("./STATS_LMEM_2022_InvSimpson_noPseudo_RELEVEL_WT31")
FileName <- paste(Title,time,".txt")
capture.output(rich_fac3.1.lme,file=FileName)
```


```
############ CREATE PLOTS - ALPHA DIV ############ 
############ CREATE PLOTS - ALPHA DIV ############ 
############ CREATE PLOTS - ALPHA DIV ############ 

# REFERENCE:
# https://rstudio-pubs-static.s3.amazonaws.com/330760_8bba830836324bf6b100d4e76f49e3d2.html#alpha-diversity


###### ALPHA DIVERSITY----------------------------------------------------------------------

mycolours <- c("#f46040", "#1dace8","#0c5979")
boxplot.chao1 <- ggboxplot(rich_fac2, x = "experiment.stage", y = "Chao1", 
  color = "group", palette = mycolours, facet.by = "temperature")

mycolours <- c("#f46040", "#1dace8","#0c5979")
boxplot.InvSimpson <- ggboxplot(rich_fac2, x = "experiment.stage", y = "InvSimpson", 
  color = "group", palette = mycolours, facet.by = "temperature")


time<-format(Sys.time(),"%d_%m_%Y")
Title <- paste("ALPHADIVERSITY_SUMMARY_3_test_NEW")
FileName <- paste(Title,time,".jpg")
#jpeg(file=FileName, width = 2220, height = 1280, res = 200)
jpeg(file=FileName, width = 2500, height = 3500, res = 500)
grid.arrange(boxplot.chao1, boxplot.InvSimpson, nrow = 2)
dev.off() 


alpha11.1 <- ggplot(data=rich_fac2, aes(x=experiment.stage, y=Chao1, fill=group)) +
  geom_boxplot(position =position_dodge(width  =.8)) +
  #geom_bar(position = 'dodge', stat="identity") +
  #geom_text(aes(label=paste(experiment.stage, "%")), position=position_dodge(width=0.9), vjust=-0.25) +
  facet_grid(. ~ temperature) + 
  theme(legend.position="none") +
  theme_bw()
alpha11 <- alpha11.1 + theme(legend.position="top") + scale_fill_manual(values = c("#f68c74", "#72c8eb"))
alpha11

alpha22.1 <- ggplot(data=rich_fac2, aes(x=experiment.stage, y=InvSimpson, fill=group)) +
  geom_boxplot(position =position_dodge(width  =.8)) +
  #geom_bar(position = 'dodge', stat="identity") +
  #geom_text(aes(label=paste(experiment.stage, "%")), position=position_dodge(width=0.9), vjust=-0.25) +
  facet_grid(. ~ temperature) + 
  theme(legend.position="none") +
  theme_bw()
alpha22 <- alpha22.1 + theme(legend.position="top") + scale_fill_manual(values = c("#f68c74", "#72c8eb"))
alpha22


time<-format(Sys.time(),"%d_%m_%Y")
Title <- paste("ALPHADIVERSITY_SUMMARY_3_test_NEW2")
FileName <- paste(Title,time,".jpg")
#jpeg(file=FileName, width = 2220, height = 1280, res = 200)
jpeg(file=FileName, width = 2500, height = 3000, res = 500)
grid.arrange(alpha11, alpha22, nrow = 2)
dev.off() 


#### EXPORT CHAO1 and INV_SIMPSON, GROUPED BY: SS vs WT - AND - INDIVIDUAL STRAINS ---------------------------------------
alpha11.1 <- ggplot(data=rich_fac2, aes(x=experiment.stage, y=Chao1, fill=group)) +
  geom_boxplot(position =position_dodge(width  =.8)) +
  #geom_bar(position = 'dodge', stat="identity") +
  #geom_text(aes(label=paste(experiment.stage, "%")), position=position_dodge(width=0.9), vjust=-0.25) +
  facet_grid(. ~ temperature) + 
  theme(legend.position="none") +
  theme_bw()
alpha11 <- alpha11.1 + theme(legend.position="none") + scale_fill_manual(values = c("#f68c74", "#72c8eb"))
#
alpha12.1 <- ggplot(data=rich_fac2, aes(x=experiment.stage, y=InvSimpson, fill=group)) +
  geom_boxplot(position =position_dodge(width  =.8)) +
  #geom_bar(position = 'dodge', stat="identity") +
  #geom_text(aes(label=paste(experiment.stage, "%")), position=position_dodge(width=0.9), vjust=-0.25) +
  facet_grid(. ~ temperature) + 
  theme(legend.position="none") +
  theme_bw()
alpha12 <- alpha12.1 + theme(legend.position="none") + scale_fill_manual(values = c("#f68c74", "#72c8eb"))
#
alpha13.1 <- ggplot(data=rich_fac2, aes(x=strain.type, y=Chao1, fill=group)) +
      theme_bw() +
  scale_fill_brewer(palette="Dark2") +
  geom_boxplot(position =position_dodge(width  =.8)) +
  #geom_bar(position = 'dodge', stat="identity") +
  #geom_text(aes(label=paste(experiment.stage, "%")), position=position_dodge(width=0.9), vjust=-0.25) +
  #facet_grid(. ~ heat.tolerance) + 
  theme(axis.text.x = element_text(angle = 45, hjust = 1))
alpha13 <- alpha13.1 + scale_fill_manual(values = c("#f68c74", "#72c8eb")) + theme(legend.position="none") 
#
alpha14.1 <- ggplot(data=rich_fac2, aes(x=strain.type, y=InvSimpson, fill=group)) +
    theme_bw() +
  geom_boxplot(position =position_dodge(width  =.8)) +        
  scale_fill_brewer(palette="Dark2") +
    #geom_bar(position = 'dodge', stat="identity") +
  #geom_text(aes(label=paste(experiment.stage, "%")),   position=position_dodge(width=0.9), vjust=-0.25) +
  #facet_grid(. ~strain.type) + 
  theme(legend.position="none", axis.text.x = element_text(angle = 45, hjust = 1))
alpha14 <- alpha14.1 + theme(legend.position="none") + scale_fill_manual(values = c("#f68c74", "#72c8eb"))
#

time<-format(Sys.time(),"%d_%m_%Y")
Title <- paste("ALPHADIVERSITY_SUMMARY_4b_Elevated_TEST")
FileName <- paste(Title,time,".jpg")
#jpeg(file=FileName, width = 2220, height = 1280, res = 200)
jpeg(file=FileName, width = 4500, height = 2500, res = 500)
grid.arrange(alpha11, alpha13, alpha12, alpha14, nrow = 2)
dev.off()
```


```
###################### Beta diversity STATISTICS ################################
###################### Beta diversity STATISTICS ################################
###################### Beta diversity STATISTICS ################################

######## BETA DIVERSITY - BRAY CURTIS - STATS -------------------------------------------

# set seed before analysis in order to generate the same outcome every time
set.seed(1)
################################

# Calculating dissimilarity matrix for SELECTED SAMPLES:
DistBC.coral.merged = phyloseq::distance(merged_data, method = "bray") 

# GENERATE METADATA FOR ADONIS
dat.coral.merged = as(sample_data(merged_data), "data.frame")

### ALTERNATIVELY SUPPLY OTU TABLE, 
#OTU1 = as(t(otu_table(class4.clean.sub)), "matrix")
# add "method = "bray" to adonis2 function..

### or CREATE DISTANCE MATRIX WITH FUNCTION vegdist from ASV table and take it instead of OTU1.
#dis <- vegdist(OTU1, method="bray")
# IF MATRIX IS SUPPLIED, ADONIS DOES NOT PERFORM DISTANCE CALCULATIONS. So no need to additionally add "method = "bray" when distance matrix is supplied.


adonis2_beta.merged <- adonis2(DistBC.coral.merged ~ group * experiment.stage * temperature, sqrt.dist = FALSE, add = FALSE, by = "terms", data = dat.coral.merged, permutations = 999)
print(adonis2_beta.merged)

#
Title <- paste("./STATS_Adonis2_Beta_Bray_2022_noPseudo")
FileName <- paste(Title,time,".txt")
capture.output(adonis2_beta.merged,file=FileName)


# REFERENCE:
# https://archetypalecology.wordpress.com/2018/02/21/permutational-multivariate-analysis-of-variance-permanova-in-r-preliminary/


######################################################################
### JACCARD
######################################################################
# Calculating dissimilarity matrix for SELECTED SAMPLES:
DistJCC.coral.merged = phyloseq::distance(merged_data, method = "jaccard") 

# GENERATE METADATA FOR ADONIS
dat.coral.merged = as(sample_data(merged_data), "data.frame")


adonis2_beta.merged <- adonis2(DistJCC.coral.merged ~ group * experiment.stage * temperature, sqrt.dist = FALSE, add = FALSE, by = "terms", data = dat.coral.merged, permutations = 999)
print(adonis2_beta.merged)

#
Title <- paste("./STATS_Adonis2_Beta_Bray_2022_noPseudo")
FileName <- paste(Title,time,".txt")
capture.output(adonis2_beta.merged,file=FileName)


# REFERENCE:
# https://archetypalecology.wordpress.com/2018/02/21/permutational-multivariate-analysis-of-variance-permanova-in-r-preliminary/


# FOR UNIFRAC STATS SEE CHUNKS BELOW
```


```
###################### Beta diversity DISPERSAL ################################
###################### Beta diversity DISPERSAL ################################
###################### Beta diversity DISPERSAL ################################

### BETA DIVERSITY DISPERSAL Bray curtis ------------------------------------------------

# REFERENCE:
# https://www.researchgate.net/post/How_should_I_correctly_manage_PERMANOVA_for_factors_with_interactions

#################################################################
# GROUP COMPARISONS
#################################################################
mod1 <- betadisper(DistBC.coral.merged, sample_data(merged_data)$group)
mod1
mod1.permut <- permutest(mod1, pairwise=TRUE, permutations=999)
mod1.permut

anova(mod1)
TukeyHSD(mod1)

Title <- paste("./STATS_Beta_Betadisper_2022_noPseudo_GROUP")
FileName <- paste(Title,time,".txt")
capture.output(mod1.permut,file=FileName)


####### ALSO POSSIBLE TO RUN THIS WITH AN ANOVA...
####### Run Test
#anova(mod1)
### Tukey's Honest Significant Differences
#mod.HSD <- TukeyHSD(mod1)
#mod.HSD
#plot(mod.HSD)

op <- par(mfrow = c(3, 2), mar = c(6, 4, 1, 2))
## Plot the groups and distances to centroids on the
## first two PCoA axes
pdf('Dispersal_SS_WT_PCOA_GROUP_merged.pdf')
plot(mod1) # plot with spider hull
#plot(mod1, hull=FALSE, ellipse=TRUE, conf = 0.95) 
#ordihull(mod, groups = sample_data(coral.sub)$group, draw="polygon", col="grey60")
#ordihull(mod, groups = sample_data(coral.sub)$group, draw="polygon", col=gray.colors(8, start = 0.4, end = 0.9, gamma = 8.2, alpha = NULL))
par(op)
dev.off()

DF1 <- data.frame(group = mod1$group, distances = mod1$distances)
### EXPORT BOXPLOT WITH DISTANCES IN Y AXIS-------------------------------------------------------
time<-format(Sys.time(),"%d_%m_%Y")
Title <- paste("Dispersal_group")
FileName <- paste(Title,time,".jpg")
#jpeg(file=FileName, width = 2220, height = 1280, res = 200)
jpeg(file=FileName, width = 5000, height = 4000, res = 800)
par(mfrow = c(1,1))
ggplot(data=DF1, aes(x=group, y=distances, fill=NULL)) +
  geom_boxplot(position =position_dodge(width  =.8)) +
  #geom_bar(position = 'dodge', stat="identity") +
  #geom_text(aes(label=paste(experiment.stage, "%")), position=position_dodge(width=0.9), vjust=-0.25) +
  #facet_grid(. ~strain.type) + 
  theme(legend.position="none", axis.text.x = element_text(angle = 45, hjust = 1)) +
  theme_bw() +
  theme(axis.text.x = element_text(angle = 45, hjust = 1)) 
dev.off() 


#################################################################
# HEAT.TOLERANCE COMPARISONS
#################################################################
mod2 <- betadisper(DistBC.coral.merged, sample_data(merged_data)$heat.tolerance)
mod2
mod2.permut <- permutest(mod2, pairwise=TRUE, permutations=999)
mod2.permut

anova(mod2)
TukeyHSD(mod2)

Title <- paste("./STATS_Beta_Betadisper_2022_noPseudo_HEAT.TOLERANCE")
FileName <- paste(Title,time,".txt")
capture.output(mod2.permut,file=FileName)


op <- par(mfrow = c(3, 2), mar = c(6, 4, 1, 2))
## Plot the groups and distances to centroids on the
## first two PCoA axes
pdf('Dispersal_SS_WT_PCOA_Heat.Tolerance.merged.pdf')
plot(mod2)
#ordihull(mod, groups = sample_data(coral.sub)$group, draw="polygon", col="grey60")
#ordihull(mod, groups = sample_data(coral.sub)$group, draw="polygon", col=gray.colors(8, start = 0.4, end = 0.9, gamma = 8.2, alpha = NULL))
par(op)
dev.off()

DF2 <- data.frame(group = mod2$group, distances = mod2$distances)
### EXPORT BOXPLOT WITH DISTANCES IN Y AXIS-------------------------------------------------------
time<-format(Sys.time(),"%d_%m_%Y")
Title <- paste("Dispersal_heat.tolerance")
FileName <- paste(Title,time,".jpg")
#jpeg(file=FileName, width = 2220, height = 1280, res = 200)
jpeg(file=FileName, width = 5000, height = 4000, res = 800)
par(mfrow = c(1,1))
ggplot(data=DF2, aes(x=group, y=distances, fill=NULL)) +
  geom_boxplot(position =position_dodge(width  =.8)) +
  #geom_bar(position = 'dodge', stat="identity") +
  #geom_text(aes(label=paste(experiment.stage, "%")), position=position_dodge(width=0.9), vjust=-0.25) +
  #facet_grid(. ~strain.type) + 
  theme(legend.position="none", axis.text.x = element_text(angle = 45, hjust = 1)) +
  theme_bw() +
  theme(axis.text.x = element_text(angle = 45, hjust = 1)) 
dev.off() 


#################################################################
# Experiment Stage COMPARISONS
#################################################################
mod3 <- betadisper(DistBC.coral.merged, sample_data(merged_data)$experiment.stage)
mod3
mod3.permut <- permutest(mod3, pairwise=TRUE, permutations=999)
mod3.permut

anova(mod3)
TukeyHSD(mod3)

Title <- paste("./STATS_Beta_Betadisper_2022_noPseudo_EXPERIMENT.STAGE")
FileName <- paste(Title,time,".txt")
capture.output(mod3.permut,file=FileName)


op <- par(mfrow = c(3, 2), mar = c(6, 4, 1, 2))
## Plot the groups and distances to centroids on the
## first two PCoA axes
pdf('Dispersal_SS_WT_PCOA_Experiment.stage.merged.pdf')
plot(mod3)
#ordihull(mod, groups = sample_data(coral.sub)$group, draw="polygon", col="grey60")
#ordihull(mod, groups = sample_data(coral.sub)$group, draw="polygon", col=gray.colors(8, start = 0.4, end = 0.9, gamma = 8.2, alpha = NULL))
par(op)
dev.off()

DF3 <- data.frame(group = mod3$group, distances = mod3$distances)
### EXPORT BOXPLOT WITH DISTANCES IN Y AXIS-------------------------------------------------------
time<-format(Sys.time(),"%d_%m_%Y")
Title <- paste("Dispersal_Experiment.Stage")
FileName <- paste(Title,time,".jpg")
#jpeg(file=FileName, width = 2220, height = 1280, res = 200)
jpeg(file=FileName, width = 5000, height = 4000, res = 800)
par(mfrow = c(1,1))
ggplot(data=DF3, aes(x=group, y=distances, fill=NULL)) +
  geom_boxplot(position =position_dodge(width  =.8)) +
  #geom_bar(position = 'dodge', stat="identity") +
  #geom_text(aes(label=paste(experiment.stage, "%")), position=position_dodge(width=0.9), vjust=-0.25) +
  #facet_grid(. ~strain.type) + 
  theme(legend.position="none", axis.text.x = element_text(angle = 45, hjust = 1)) +
  theme_bw() +
  theme(axis.text.x = element_text(angle = 45, hjust = 1)) 
dev.off() 


#################################################################
# Strain.type COMPARISONS
#################################################################
mod4 <- betadisper(DistBC.coral, sample_data(class4.clean.sub)$strain.type)
mod4
mod4.permut <- permutest(mod4, pairwise=TRUE, permutations=999)
mod4.permut

anova(mod4)
TukeyHSD(mod4)

Title <- paste("./STATS_Beta_Betadisper_2022_noPseudo_STRAIN.TYPE")
FileName <- paste(Title,time,".txt")
capture.output(mod4.permut,file=FileName)


op <- par(mfrow = c(3, 2), mar = c(6, 4, 1, 2))
## Plot the groups and distances to centroids on the
## first two PCoA axes
pdf('Dispersal_SS_WT_PCOA_strain.type.pdf')
plot(mod4)
#plot(mod4, hull=FALSE, ellipse=TRUE, conf = 0.95) 
#ordihull(mod, groups = sample_data(coral.sub)$group, draw="polygon", col="grey60")
#ordihull(mod, groups = sample_data(coral.sub)$group, draw="polygon", col=gray.colors(8, start = 0.4, end = 0.9, gamma = 8.2, alpha = NULL))
par(op)
dev.off()

DF4 <- data.frame(group = mod4$group, distances = mod4$distances)
### EXPORT BOXPLOT WITH DISTANCES IN Y AXIS-------------------------------------------------------
time<-format(Sys.time(),"%d_%m_%Y")
Title <- paste("Dispersal_strain.type")
FileName <- paste(Title,time,".jpg")
#jpeg(file=FileName, width = 2220, height = 1280, res = 200)
jpeg(file=FileName, width = 5000, height = 4000, res = 800)
par(mfrow = c(1,1))
ggplot(data=DF4, aes(x=group, y=distances, fill=NULL)) +
  geom_boxplot(position =position_dodge(width  =.8)) +
  #geom_bar(position = 'dodge', stat="identity") +
  #geom_text(aes(label=paste(experiment.stage, "%")), position=position_dodge(width=0.9), vjust=-0.25) +
  #facet_grid(. ~strain.type) + 
  theme(legend.position="none", axis.text.x = element_text(angle = 45, hjust = 1)) +
  theme_bw() +
  theme(axis.text.x = element_text(angle = 45, hjust = 1)) 
dev.off() 


#################################################################
# Temperature COMPARISONS
#################################################################
mod5 <- betadisper(DistBC.coral.merged, sample_data(merged_data)$temperature)
mod5
mod5.permut <- permutest(mod5, pairwise=TRUE, permutations=999)
mod5.permut

anova(mod5)
TukeyHSD(mod5)

Title <- paste("./STATS_Beta_Betadisper_2022_noPseudo_TEMPERATURE")
FileName <- paste(Title,time,".txt")
capture.output(mod5.permut,file=FileName)


op <- par(mfrow = c(3, 2), mar = c(6, 4, 1, 2))
## Plot the groups and distances to centroids on the
## first two PCoA axes
pdf('Dispersal_SS_WT_PCOA_temperature.merged.pdf')
plot(mod5)
#ordihull(mod, groups = sample_data(coral.sub)$group, draw="polygon", col="grey60")
#ordihull(mod, groups = sample_data(coral.sub)$group, draw="polygon", col=gray.colors(8, start = 0.4, end = 0.9, gamma = 8.2, alpha = NULL))
par(op)
dev.off()

DF5 <- data.frame(group = mod5$group, distances = mod5$distances)
### EXPORT BOXPLOT WITH DISTANCES IN Y AXIS-------------------------------------------------------
time<-format(Sys.time(),"%d_%m_%Y")
Title <- paste("Dispersal_temperature")
FileName <- paste(Title,time,".jpg")
#jpeg(file=FileName, width = 2220, height = 1280, res = 200)
jpeg(file=FileName, width = 5000, height = 4000, res = 800)
par(mfrow = c(1,1))
ggplot(data=DF5, aes(x=group, y=distances, fill=NULL)) +
  geom_boxplot(position =position_dodge(width  =.8)) +
  #geom_bar(position = 'dodge', stat="identity") +
  #geom_text(aes(label=paste(experiment.stage, "%")), position=position_dodge(width=0.9), vjust=-0.25) +
  #facet_grid(. ~strain.type) + 
  theme(legend.position="none", axis.text.x = element_text(angle = 45, hjust = 1)) +
  theme_bw() +
  theme(axis.text.x = element_text(angle = 45, hjust = 1)) 
dev.off()
```


```
###################### Beta diversity Unifrac Statistics ################################
###################### Beta diversity Unifrac Statistics ################################
###################### Beta diversity Unifrac Statistics ################################


# Set seed
set.seed(1)


# Calculating dissimilarity matrix for SELECTED SAMPLES:
DistwUni.coral.merged = phyloseq::distance(merged_data, method = "wunifrac") 

# GENERATE METADATA FOR ADONIS
dat.coral.merged = as(sample_data(merged_data), "data.frame")


### ADONIS BETA DIVERSITY - UNIFRAC -----------------------------------------------
adonis2_beta_uni <- adonis2(DistwUni.coral.merged ~ group * experiment.stage * temperature, sqrt.dist = FALSE, add = FALSE, by = "terms", data = dat.coral.merged, permutations = 999)
print(adonis2_beta_uni)

#
Title <- paste("./Adonis2_Beta_UNI")
FileName <- paste(Title,time,".txt")
capture.output(adonis2_beta_uni,file=FileName)


### BETA DIVERSITY DISPERSAL UNIFRAC ------------------------------------------------
mod1.merged.wUni <- betadisper(DistwUni.coral.merged, sample_data(merged_data)$group)
mod1.merged.wUni
permutest(mod1.merged.wUni, pairwise=TRUE, permutations=999)

mod2.merged.wUni <- betadisper(DistwUni.coral.merged, sample_data(merged_data)$experiment.stage)
mod2.merged.wUni
permutest(mod2.merged.wUni, pairwise=TRUE, permutations=999)

mod3.merged.wUni <- betadisper(DistwUni.coral.merged, sample_data(merged_data)$temperature)
mod3.merged.wUni
permutest(mod3.merged.wUni, pairwise=TRUE, permutations=999)


####### ALSO POSSIBLE TO RUN THIS WITH AN ANOVA...
####### Run Test
#anova(mod1.merged)
### Tukey's Honest Significant Differences
#mod.HSD <- TukeyHSD(mod1.merged)
#mod.HSD
#plot(mod.HSD)

op <- par(mfrow = c(3, 2), mar = c(6, 4, 1, 2))
## Plot the groups and distances to centroids on the
## first two PCoA axes
pdf('Dispersal_wUni_SS_WT_PCOA_GROUP_merged.pdf')
plot(mod1.merged.wUni) # plot with spider hull
#plot(mod1, hull=FALSE, ellipse=TRUE, conf = 0.95) 
#ordihull(mod, groups = sample_data(coral.sub)$group, draw="polygon", col="grey60")
#ordihull(mod, groups = sample_data(coral.sub)$group, draw="polygon", col=gray.colors(8, start = 0.4, end = 0.9, gamma = 8.2, alpha = NULL))
par(op)
dev.off()
```


```
############ CREATE PLOTS - BETA DIVERSITY ############ 
############ CREATE PLOTS - BETA DIVERSITY ############ 
############ CREATE PLOTS - BETA DIVERSITY ############ 


# Data 'merged' is used here to match visuals with statistics

### BRAY
class.pcoa_bray_merged <- ordinate(
  physeq = merged_data, 
  method = "PCoA", 
  distance = "bray")

b0 <- plot_ordination(
  physeq = merged_data,
  ordination = class.pcoa_bray_merged,
  color = "Group3",
  shape = "temperature",
  axes = 1:3,
  title = NULL) +
  geom_point(aes(color = Group3), size = 6) +
  theme_bw(base_size = 20) +
  #stat_ellipse(type = "norm", linetype = 2)
  #stat_ellipse(aes(group=group),type = "norm", linetype = 1) +
  stat_ellipse(aes(group=Group3),type = "norm", linetype = 1) +
  #coord_flip()
  scale_y_reverse() +
  scale_x_reverse()
#b1aa <- b0 + scale_color_manual(values = c("#f46040", "#ac270a", "#1dace8","#0c5979")) 
b1aa <- b0 + scale_color_manual(values = c("#ddb88e", "#f34c28","#ddb88e", "#f34c28","#849ba0","#1dace8","#849ba0","#1dace8")) + theme(legend.position = "none") 


time<-format(Sys.time(),"%d_%m_%Y")
Title <- paste("BETA_DIVERSITY_SUMMARY_BRAY_Fig1_new2_NON_RAREIF1")
FileName <- paste(Title,time,".jpg")
#jpeg(file=FileName, width = 2220, height = 1280, res = 200)
jpeg(file=FileName, width =7000, height = 5000, res = 650)
grid.arrange(b1aa, nrow = 1)
#plot_grid(b1a, b3a, align = "h", nrow = 1, rel_width = c(1/2, 1/2))
dev.off() 


#### PLOT INDIVIDUAL STRAINS
class.pcoa_bray <- ordinate(
  physeq = class4.clean.sub, 
  method = "PCoA", 
  distance = "bray")

b0 <- plot_ordination(
  physeq = class4.clean.sub,
  ordination = class.pcoa_bray,
  color = "heat.tolerance",
  shape = "group",
  axes = 1:3,
  title = NULL) +
  geom_point(aes(color = heat.tolerance), size = 6) +
  theme_bw(base_size = 20)
  #stat_ellipse(type = "norm", linetype = 2)
  #coord_flip() 
  #scale_y_reverse()
b1a <- b0 + scale_color_manual(values = c("#f34c28", "#ddb88e", "#1dace8")) #+ scale_shape_manual(values = c(21, 19, 24, 17))

b2a <- plot_ordination(
  physeq = class4.clean.sub,
  ordination = class.pcoa_bray,
  color = "strain.type",
  shape = "experiment.stage",
  axes = 1:3,
  title = NULL) +
  geom_point(aes(color = strain.type), size = 6) +
  theme_bw(base_size = 20) 
b3a <- b2a + scale_color_manual(values = c("#F24D29", "#E5C4A1", "#FCD16B", "#CC8B3C", "#D8A49B", "#B62A3D",  "#A35E60", "#541F12", "#D1362F", "#CB9E23", "#AEA8A8", "#76A08A", "#27223C", "#CECD7B", "#7496D2", "#C7CEF6", "#2E604A", "#1C366B", "#1DACE8")) + theme(legend.position = "none") #+ scale_shape_manual(values = c(17, 16, 18, 15))

time<-format(Sys.time(),"%d_%m_%Y")
Title <- paste("BETA_DIVERSITY_SUMMARY_BRAY_SFig1")
FileName <- paste(Title,time,".jpg")
#jpeg(file=FileName, width = 2220, height = 1280, res = 200)
jpeg(file=FileName, width = 5000, height = 7000, res = 500)
grid.arrange(b1a, b3a, nrow = 2)
#plot_grid(b1a, b3a, align = "h", nrow = 1, rel_width = c(1/2, 1/2))
dev.off() 


# EXPORTED PLOTS ONCE WITH AND ONCE WITHOUT LEGEND, THEN MERGED PLOTS AND LEGEND IN DIFFERENT SOFTWARE -> OTHERWISE, PLOTS WOULD NOT HAVE THE SAME SIZE DUE TO LEGEND SIZE DIFFERENCES.
time<-format(Sys.time(),"%d_%m_%Y")
Title <- paste("BETA_DIVERSITY_SUMMARY_BRAY_Fig2_TEST5")
FileName <- paste(Title,time,".jpg")
#jpeg(file=FileName, width = 2220, height = 1280, res = 200)
jpeg(file=FileName, width = 4000, height = 7000, res = 500)
grid.arrange(b1aa, b3a, nrow = 2)
dev.off() 


################ UNIFRAC --- Heat TOLERANCE vs STRAINS -------------------------------
################ UNIFRAC --- Heat TOLERANCE vs STRAINS -------------------------------
################ UNIFRAC --- Heat TOLERANCE vs STRAINS -------------------------------

### Weighted Unifrac
class.pcoa_wunifrac <- ordinate(
  physeq = merged_data, 
  method = "PCoA", 
  distance = "wunifrac")

b0 <- plot_ordination(
  physeq = merged_data,
  ordination = class.pcoa_wunifrac,
  color = "group",
  shape = "group",
  axes = 1:3,
  title = NULL) +
  geom_point(aes(color = group), size = 6) +
  theme_bw(base_size = 20) +
  #stat_ellipse(type = "norm", linetype = 2)
  #stat_ellipse(aes(group=group),type = "norm", linetype = 1) +
  stat_ellipse(aes(group=group),type = "norm", linetype = 1)
  #coord_flip()
#b1aa <- b0 + scale_color_manual(values = c("#f46040", "#ac270a", "#1dace8","#0c5979")) 
b1aa <- b0 + scale_color_manual(values = c("#f34c28", "#1dace8","#ddb88e", "#f34c28","#849ba0","#1dace8","#849ba0","#1dace8")) # + theme(legend.position = "none") 


time<-format(Sys.time(),"%d_%m_%Y")
Title <- paste("BETA_DIVERSITY_SUMMARY_wUNIFRAC_Fig1_new2_NON_RAREIF1")
FileName <- paste(Title,time,".jpg")
#jpeg(file=FileName, width = 2220, height = 1280, res = 200)
jpeg(file=FileName, width =6500, height = 5000, res = 650)
grid.arrange(b1aa, nrow = 1)
#plot_grid(b1a, b3a, align = "h", nrow = 1, rel_width = c(1/2, 1/2))
dev.off() 


#### PLOT INDIVIDUAL STRAINS
class.pcoa_wunifrac2 <- ordinate(
  physeq = class4.clean.sub, 
  method = "PCoA", 
  distance = "wunifrac")

b0 <- plot_ordination(
  physeq = class4.clean.sub,
  ordination = class.pcoa_wunifrac2,
  color = "group",
  shape = "group",
  axes = 1:3,
  title = NULL) +
  geom_point(aes(color = group), size = 6) +
  theme_bw(base_size = 20)
  #stat_ellipse(type = "norm", linetype = 2)
  #coord_flip() 
  #scale_y_reverse()
b1a <- b0 + scale_color_manual(values = c("#f34c28", "#1dace8")) #+ scale_shape_manual(values = c(21, 19, 24, 17))

b2a <- plot_ordination(
  physeq = class4.clean.sub,
  ordination = class.pcoa_wunifrac2,
  color = "strain.type",
  shape = "group",
  axes = 1:3,
  title = NULL) +
  geom_point(aes(color = strain.type), size = 6) +
  theme_bw(base_size = 20) 
b3a <- b2a + scale_color_manual(values = c("#F24D29", "#E5C4A1", "#FCD16B", "#CC8B3C", "#D8A49B", "#B62A3D",  "#A35E60", "#541F12", "#D1362F", "#CB9E23", "#AEA8A8", "#76A08A", "#27223C", "#CECD7B", "#7496D2", "#C7CEF6", "#2E604A", "#1C366B", "#1DACE8")) #+ theme(legend.position = "none") #+ scale_shape_manual(values = c(17, 16, 18, 15))

time<-format(Sys.time(),"%d_%m_%Y")
Title <- paste("BETA_DIVERSITY_SUMMARY_wUNIFRAC_SFig1")
FileName <- paste(Title,time,".jpg")
#jpeg(file=FileName, width = 2220, height = 1280, res = 200)
jpeg(file=FileName, width = 5000, height = 7000, res = 500)
grid.arrange(b1aa, b3a, nrow = 2)
#plot_grid(b1a, b3a, align = "h", nrow = 1, rel_width = c(1/2, 1/2))
dev.off() 


################ Jaccard --- Heat TOLERANCE vs STRAINS -------------------------------
################ Jaccard --- Heat TOLERANCE vs STRAINS -------------------------------
################ Jaccard --- Heat TOLERANCE vs STRAINS -------------------------------

### Weighted Unifrac
class.pcoa_jaccard <- ordinate(
  physeq = merged_data, 
  method = "PCoA", 
  distance = "jaccard")

b0jacc <- plot_ordination(
  physeq = merged_data,
  ordination = class.pcoa_jaccard,
  color = "group",
  shape = "group",
  axes = 1:3,
  title = NULL) +
  geom_point(aes(color = group), size = 6) +
  theme_bw(base_size = 20) +
  #stat_ellipse(type = "norm", linetype = 2)
  #stat_ellipse(aes(group=group),type = "norm", linetype = 1) +
  stat_ellipse(aes(group=group),type = "norm", linetype = 1) +
  scale_y_reverse() +
  scale_x_reverse()
#b1aa <- b0 + scale_color_manual(values = c("#f46040", "#ac270a", "#1dace8","#0c5979")) 
b1jacc <- b0jacc + scale_color_manual(values = c("#f34c28", "#1dace8","#ddb88e", "#f34c28","#849ba0","#1dace8","#849ba0","#1dace8")) # + theme(legend.position = "none") 


time<-format(Sys.time(),"%d_%m_%Y")
Title <- paste("BETA_DIVERSITY_SUMMARY_jaccard_Fig1_new2_NON_RAREIF1")
FileName <- paste(Title,time,".jpg")
#jpeg(file=FileName, width = 2220, height = 1280, res = 200)
jpeg(file=FileName, width =6500, height = 5000, res = 650)
grid.arrange(b1jacc, nrow = 1)
#plot_grid(b1a, b3a, align = "h", nrow = 1, rel_width = c(1/2, 1/2))
dev.off() 


#### PLOT INDIVIDUAL STRAINS
class.pcoa_jaccard2 <- ordinate(
  physeq = class4.clean.sub, 
  method = "PCoA", 
  distance = "jaccard")

b0jacc <- plot_ordination(
  physeq = class4.clean.sub,
  ordination = class.pcoa_jaccard2,
  color = "group",
  shape = "group",
  axes = 1:3,
  title = NULL) +
  geom_point(aes(color = group), size = 6) +
  theme_bw(base_size = 20)
  #stat_ellipse(type = "norm", linetype = 2)
  #coord_flip() 
  #scale_y_reverse()
b1jacca <- b0jacc + scale_color_manual(values = c("#f34c28", "#1dace8")) #+ scale_shape_manual(values = c(21, 19, 24, 17))

b2jacc <- plot_ordination(
  physeq = class4.clean.sub,
  ordination = class.pcoa_jaccard2,
  color = "strain.type",
  shape = "group",
  axes = 1:3,
  title = NULL) +
  geom_point(aes(color = strain.type), size = 6) +
  theme_bw(base_size = 20) 
b3jacc <- b2jacc + scale_color_manual(values = c("#F24D29", "#E5C4A1", "#FCD16B", "#CC8B3C", "#D8A49B", "#B62A3D",  "#A35E60", "#541F12", "#D1362F", "#CB9E23", "#AEA8A8", "#76A08A", "#27223C", "#CECD7B", "#7496D2", "#C7CEF6", "#2E604A", "#1C366B", "#1DACE8")) #+ theme(legend.position = "none") #+ scale_shape_manual(values = c(17, 16, 18, 15))

time<-format(Sys.time(),"%d_%m_%Y")
Title <- paste("BETA_DIVERSITY_SUMMARY_wUNIFRAC_SFig1")
FileName <- paste(Title,time,".jpg")
#jpeg(file=FileName, width = 2220, height = 1280, res = 200)
jpeg(file=FileName, width = 5000, height = 7000, res = 500)
grid.arrange(b1jacc, b3jacc, nrow = 2)
#plot_grid(b1a, b3a, align = "h", nrow = 1, rel_width = c(1/2, 1/2))
dev.off() 


time<-format(Sys.time(),"%d_%m_%Y")
Title <- paste("BETA_DIVERSITY_SUMMARY_wUNIFRAC_Jacc_SFig1")
FileName <- paste(Title,time,".jpg")
#jpeg(file=FileName, width = 2220, height = 1280, res = 200)
jpeg(file=FileName, width = 10000, height = 7000, res = 500)
grid.arrange(b1aa, b1jacc, b3a, b3jacc, nrow = 2)
#plot_grid(b1a, b3a, align = "h", nrow = 1, rel_width = c(1/2, 1/2))
dev.off() 


#REFERENCE: 
#  http://opencolor.tools/palettes/wesanderson/
```


```
###################### BAR PLOTS TO EXPLORE ABUNDANCES ######################
###################### BAR PLOTS TO EXPLORE ABUNDANCES ######################
###################### BAR PLOTS TO EXPLORE ABUNDANCES ######################

# RELATIVE ABUNDANCES


# MAKES FONT ITALIC
guide_italics <- guides(fill = guide_legend(label.theme = element_text(
  size = 15,
  face = "italic", colour = "Black", angle = 0
)))


# MAKE TAXA
coral.class <- class4.clean.sub %>% 
  tax_glom(taxrank = "Class")
coral.family <- class4.clean.sub %>% 
  tax_glom(taxrank = "Family")
coral.genus <- class4.clean.sub %>% 
  tax_glom(taxrank = "Genus")
coral.species <- class4.clean.sub %>% 
  tax_glom(taxrank = "Species")


################################################
# GENERATE FULL OTU TABLE
# GENERATE FULL OTU TABLE
# GENERATE FULL OTU TABLE

# Transform counts in relative abundance and select most abundant families
percent <- transform_sample_counts(merged_data, function(x) 100 * x/sum(x))
sample_data(percent)$Merge1 <- as.factor(sample_data(percent)$Merge1)
#
#
#FILL MISSING TAXONOMY
tax <- data.frame(tax_table(percent))
#
tax.clean <- data.frame(row.names = row.names(tax),
Kingdom = str_replace(tax[,1], "D_0__",""),
Phylum = str_replace(tax[,2], "D_1__",""),
Class = str_replace(tax[,3], "D_2__",""),
Order = str_replace(tax[,4], "D_3__",""),
Family = str_replace(tax[,5], "D_4__",""),
Genus = str_replace(tax[,6], "D_5__",""),
Species = str_replace(tax[,7], "D_6__",""),
stringsAsFactors = FALSE)
tax.clean[is.na(tax.clean)] <- ""
#
for (i in 1:7){ tax.clean[,i] <- as.character(tax.clean[,i])}
####### Fille holes in the tax table
tax.clean[is.na(tax.clean)] <- ""
for (i in 1:nrow(tax.clean)){
#  
if (tax.clean[i,2] == ""){
kingdom <- paste("Kingdom_", tax.clean[i,1], sep = "")
tax.clean[i, 2:7] <- kingdom
} else if (tax.clean[i,3] == ""){
phylum <- paste("Phylum_", tax.clean[i,2], sep = "")
tax.clean[i, 3:7] <- phylum
} else if (tax.clean[i,4] == ""){
class <- paste("Class_", tax.clean[i,3], sep = "")
tax.clean[i, 4:7] <- class
} else if (tax.clean[i,5] == ""){
order <- paste("Order_", tax.clean[i,4], sep = "")
tax.clean[i, 5:7] <- order
} else if (tax.clean[i,6] == ""){
family <- paste("Family_", tax.clean[i,5], sep = "")
tax.clean[i, 6:7] <- family
} else if (tax.clean[i,7] == ""){
tax.clean$Species[i] <- paste("Genus",tax.clean$Genus[i], sep = "_")
}
}
#
tax_table(percent) <- as.matrix(tax.clean)
##############################
#FILL MISSING TAXONOMY
#
# Export ASV Table
TopNOTUs = names(sort(taxa_sums(percent), TRUE))
newdat = prune_taxa(TopNOTUs, percent)
# newdatmelt1 <- psmelt(percent)
newdatmelt <- psmelt(newdat)
#
write.csv(newdatmelt,file = "FULL_OTU_TALBE_NEW_Merge1.NEW_2022.csv")


########################################################################
########################################################################
########################################################################


#### GROUP EXPERIMENTAL.STAGE
#### GROUP EXPERIMENTAL.STAGE
#### GROUP EXPERIMENTAL.STAGE


# To group samples by strain type (skip if i want all individual samples)
merged <- merge_samples(merged_data, "Group1")
sample_data(merged)$Group1 <- levels(sample_data(class4.clean.sub)$Group1)
#
#
# Transform counts in relative abundance and select most abundant families
percent <- transform_sample_counts(merged, function(x) 100 * x/sum(x))
sample_data(percent)$Group1 <- as.factor(sample_data(percent)$Group1)
#
#
#FILL MISSING TAXONOMY
tax <- data.frame(tax_table(percent))
#
tax.clean <- data.frame(row.names = row.names(tax),
Kingdom = str_replace(tax[,1], "D_0__",""),
Phylum = str_replace(tax[,2], "D_1__",""),
Class = str_replace(tax[,3], "D_2__",""),
Order = str_replace(tax[,4], "D_3__",""),
Family = str_replace(tax[,5], "D_4__",""),
#Genus = str_replace(tax[,6], "D_5__",""),
Genus = str_replace(str_replace(str_replace(tax[,6], "D_5__uncultured_Gammaproteobacterium","u.Gammaproteob."), "D_5__uncultured_","u."), "D_5__",""),
Species = str_replace(tax[,7], "D_6__",""),
stringsAsFactors = FALSE)
tax.clean[is.na(tax.clean)] <- ""
#
for (i in 1:7){ tax.clean[,i] <- as.character(tax.clean[,i])}
####### Fille holes in the tax table
tax.clean[is.na(tax.clean)] <- ""
for (i in 1:nrow(tax.clean)){
#  
if (tax.clean[i,2] == ""){
kingdom <- paste("Kingdom_", tax.clean[i,1], sep = "")
tax.clean[i, 2:7] <- kingdom
} else if (tax.clean[i,3] == ""){
phylum <- paste("Phylum_", tax.clean[i,2], sep = "")
tax.clean[i, 3:7] <- phylum
} else if (tax.clean[i,4] == ""){
class <- paste("Class_", tax.clean[i,3], sep = "")
tax.clean[i, 4:7] <- class
} else if (tax.clean[i,5] == ""){
order <- paste("Order_", tax.clean[i,4], sep = "")
tax.clean[i, 5:7] <- order
} else if (tax.clean[i,6] == ""){
family <- paste("Family_", tax.clean[i,5], sep = "")
tax.clean[i, 6:7] <- family
} else if (tax.clean[i,7] == ""){
tax.clean$Species[i] <- paste("Genus",tax.clean$Genus[i], sep = "_")
}
}
#
tax_table(percent) <- as.matrix(tax.clean)
#
##############################
#FILL MISSING TAXONOMY
#
# Export ASV Table
TopNOTUs = names(sort(taxa_sums(percent), TRUE))
newdat = prune_taxa(TopNOTUs, percent)
# newdatmelt1 <- psmelt(percent)
newdatmelt <- psmelt(newdat)
#
#write.csv(newdatmelt1,file = "ASV_TALBE_NEW_EXPERIMENT.STAGE_2022.csv")
#
# SHOW DIFFERENT GENERA
percent2<- percent %>% tax_glom(taxrank = "Genus", NArm = FALSE)
#
TopNOTUs = names(sort(taxa_sums(percent2), TRUE)[1:20])
newdat = prune_taxa(TopNOTUs, percent2)
# newdatmelt1 <- psmelt(percent)
newdatmelt1 <- psmelt(newdat)
#
newdatmelt1$Group1 <- factor(newdatmelt1$Group1, levels=c("WildType.before", "WildType.after","Selected.before", "Selected.after"))
#
levels(newdatmelt1$Group1) <- sub("WildType.before", "WT before", levels(newdatmelt1$Group1))
#
levels(newdatmelt1$Group1) <- sub("WildType.after", "WT after", levels(newdatmelt1$Group1))
#
levels(newdatmelt1$Group1) <- sub("Selected.before", "SS before", levels(newdatmelt1$Group1))
#
levels(newdatmelt1$Group1) <- sub("Selected.after", "SS after", levels(newdatmelt1$Group1))
#
getPalette = colorRampPalette(rev(brewer.pal(12, "Paired"))) 
PhylaPalette = getPalette(20)
#
alpha2 <- ggplot(newdatmelt1, aes(x = Group1, y = Abundance, fill = Genus)) +
    theme_bw() +
    guide_italics +
  geom_bar(stat = "identity", position="stack") +
      theme(legend.position="right", legend.text=element_text(size=20), axis.title.x = element_blank(), axis.text.x = element_text(angle = 45, hjust = 1)) +
  ylab("% Abundance") +
  facet_wrap(~Genus, scales= "free", nrow=7) +
  scale_fill_manual(values = PhylaPalette) 
#
#
time<-format(Sys.time(),"%d_%m_%Y")
Title <- paste("Abundances_2_Genus_Relative_RARE_GROUP_3_TEST1")
FileName <- paste(Title,time,".jpg")
jpeg(file=FileName, width = 7000, height = 11000, res = 700)
par(mfrow = c(1,1))
alpha2 + theme(strip.text.x = element_text(size = 9, color = "black", face = "italic")) + theme(axis.text=element_text(size=10),
        axis.title=element_text(size=11,face="bold"))
dev.off()   
 


#### GROUP TEMPERATURE
#### GROUP TEMPERATURE
#### GROUP TEMPERATURE
#
# To group samples by strain type (skip if i want all individual samples)
merged <- merge_samples(class4.clean.sub, "Group2")
sample_data(merged)$Group2 <- levels(sample_data(class4.clean.sub)$Group2)
#
#
# Transform counts in relative abundance and select most abundant families
percent <- transform_sample_counts(merged, function(x) 100 * x/sum(x))
sample_data(percent)$Group2 <- as.factor(sample_data(percent)$Group2)
#
#
#FILL MISSING TAXONOMY
tax <- data.frame(tax_table(percent))
#
tax.clean <- data.frame(row.names = row.names(tax),
Kingdom = str_replace(tax[,1], "D_0__",""),
Phylum = str_replace(tax[,2], "D_1__",""),
Class = str_replace(tax[,3], "D_2__",""),
Order = str_replace(tax[,4], "D_3__",""),
Family = str_replace(tax[,5], "D_4__",""),
Genus = str_replace(str_replace(str_replace(tax[,6], "D_5__uncultured_Gammaproteobacterium","u.Gammaproteob."), "D_5__uncultured_","u."), "D_5__",""),
Species = str_replace(tax[,7], "D_6__",""),
stringsAsFactors = FALSE)
tax.clean[is.na(tax.clean)] <- ""
#
for (i in 1:7){ tax.clean[,i] <- as.character(tax.clean[,i])}
####### Fille holes in the tax table
tax.clean[is.na(tax.clean)] <- ""
for (i in 1:nrow(tax.clean)){
#  
if (tax.clean[i,2] == ""){
kingdom <- paste("Kingdom_", tax.clean[i,1], sep = "")
tax.clean[i, 2:7] <- kingdom
} else if (tax.clean[i,3] == ""){
phylum <- paste("Phylum_", tax.clean[i,2], sep = "")
tax.clean[i, 3:7] <- phylum
} else if (tax.clean[i,4] == ""){
class <- paste("Class_", tax.clean[i,3], sep = "")
tax.clean[i, 4:7] <- class
} else if (tax.clean[i,5] == ""){
order <- paste("Order_", tax.clean[i,4], sep = "")
tax.clean[i, 5:7] <- order
} else if (tax.clean[i,6] == ""){
family <- paste("Family_", tax.clean[i,5], sep = "")
tax.clean[i, 6:7] <- family
} else if (tax.clean[i,7] == ""){
tax.clean$Species[i] <- paste("Genus",tax.clean$Genus[i], sep = "_")
}
}
#
tax_table(percent) <- as.matrix(tax.clean)
#
##############################
#FILL MISSING TAXONOMY
#
# Export ASV Table
TopNOTUs = names(sort(taxa_sums(percent), TRUE))
newdat = prune_taxa(TopNOTUs, percent)
# newdatmelt1 <- psmelt(percent)
newdatmelt <- psmelt(newdat)
#
write.csv(newdatmelt1,file = "ASV_TALBE_NEW_TEMPERATURE_2022.csv")
#
# SHOW DIFFERENT GENERA
percent2<- percent %>% tax_glom(taxrank = "Genus", NArm = FALSE)
#
TopNOTUs = names(sort(taxa_sums(percent2), TRUE)[1:20])
newdat = prune_taxa(TopNOTUs, percent2)
# newdatmelt1 <- psmelt(percent)
newdatmelt1 <- psmelt(newdat)
#
newdatmelt1$Group2 <- factor(newdatmelt1$Group2, levels=c("WildType.Ambient", "WildType.Elevated","Selected.Ambient", "Selected.Elevated"))
#
levels(newdatmelt1$Group2) <- sub("WildType.Ambient", "WT ambient", levels(newdatmelt1$Group2))
#
levels(newdatmelt1$Group2) <- sub("WildType.Elevated", "WT elevated", levels(newdatmelt1$Group2))
#
levels(newdatmelt1$Group2) <- sub("Selected.Ambient", "SS ambient", levels(newdatmelt1$Group2))
#
levels(newdatmelt1$Group2) <- sub("Selected.Elevated", "SS elevated", levels(newdatmelt1$Group2))
#
getPalette = colorRampPalette(rev(brewer.pal(12, "Paired"))) 
PhylaPalette = getPalette(20)
#
alpha2 <- ggplot(newdatmelt1, aes(x = Group2, y = Abundance, fill = Genus)) +
    theme_bw() +
    guide_italics +
  geom_bar(stat = "identity", position="stack") +
      theme(legend.position="right", legend.text=element_text(size=20), axis.title.x = element_blank(), axis.text.x = element_text(angle = 45, hjust = 1)) +
  ylab("% Abundance") +
  facet_wrap(~Genus, scales= "free", nrow=7) +
  scale_fill_manual(values = PhylaPalette) 
#
time<-format(Sys.time(),"%d_%m_%Y")
Title <- paste("Abundances_2_Genus_Relative_RARE_GROUP_3h")
FileName <- paste(Title,time,".jpg")
jpeg(file=FileName, width = 7000, height = 11000, res = 700)
par(mfrow = c(1,1))
alpha2 + theme(strip.text.x = element_text(size = 9, color = "black", face = "italic")) + theme(axis.text=element_text(size=10),
        axis.title=element_text(size=11,face="bold"))
dev.off()   


#### STRAIN.TYPE
#### STRAIN.TYPE
#### STRAIN.TYPE
#
# To group samples by strain type (skip if i want all individual samples)
merged <- merge_samples(merged_data, "strain.type")
sample_data(merged)$strain.type <- levels(sample_data(merged_data)$strain.type)
#
#
# Transform counts in relative abundance and select most abundant families
percent <- transform_sample_counts(merged, function(x) 100 * x/sum(x))
sample_data(percent)$strain.type <- as.factor(sample_data(percent)$strain.type)
#
#
#FILL MISSING TAXONOMY
tax <- data.frame(tax_table(percent))
#
tax.clean <- data.frame(row.names = row.names(tax),
Kingdom = str_replace(tax[,1], "D_0__",""),
Phylum = str_replace(tax[,2], "D_1__",""),
Class = str_replace(tax[,3], "D_2__",""),
Order = str_replace(tax[,4], "D_3__",""),
Family = str_replace(tax[,5], "D_4__",""),
Genus = str_replace(tax[,6], "D_5__",""),
Species = str_replace(tax[,7], "D_6__",""),
stringsAsFactors = FALSE)
tax.clean[is.na(tax.clean)] <- ""
#
for (i in 1:7){ tax.clean[,i] <- as.character(tax.clean[,i])}
####### Fille holes in the tax table
tax.clean[is.na(tax.clean)] <- ""
for (i in 1:nrow(tax.clean)){
#  
if (tax.clean[i,2] == ""){
kingdom <- paste("Kingdom_", tax.clean[i,1], sep = "")
tax.clean[i, 2:7] <- kingdom
} else if (tax.clean[i,3] == ""){
phylum <- paste("Phylum_", tax.clean[i,2], sep = "")
tax.clean[i, 3:7] <- phylum
} else if (tax.clean[i,4] == ""){
class <- paste("Class_", tax.clean[i,3], sep = "")
tax.clean[i, 4:7] <- class
} else if (tax.clean[i,5] == ""){
order <- paste("Order_", tax.clean[i,4], sep = "")
tax.clean[i, 5:7] <- order
} else if (tax.clean[i,6] == ""){
family <- paste("Family_", tax.clean[i,5], sep = "")
tax.clean[i, 6:7] <- family
} else if (tax.clean[i,7] == ""){
tax.clean$Species[i] <- paste("Genus",tax.clean$Genus[i], sep = "_")
}
}
#
tax_table(percent) <- as.matrix(tax.clean)
#
##############################
#FILL MISSING TAXONOMY
#
# Export ASV Table
TopNOTUs = names(sort(taxa_sums(percent), TRUE))
newdat = prune_taxa(TopNOTUs, percent)
# newdatmelt1 <- psmelt(percent)
newdatmelt <- psmelt(newdat)
#
write.csv(newdatmelt1,file = "FULL_OTU_TALBE_NEW_time.two.NEW_2308_2022_MERGED_DATA.csv")
#
# SHOW DIFFERENT GENERA
percent2<- percent %>% tax_glom(taxrank = "Genus", NArm = FALSE)
#
TopNOTUs = names(sort(taxa_sums(percent2), TRUE)[1:20])
newdat = prune_taxa(TopNOTUs, percent2)
# newdatmelt1 <- psmelt(percent)
newdatmelt1 <- psmelt(newdat)
#
getPalette = colorRampPalette(rev(brewer.pal(12, "Paired"))) 
PhylaPalette = getPalette(20)
#
alpha2 <- ggplot(newdatmelt1, aes(x = strain.type, y = Abundance, fill = Genus)) +
    theme_bw() +
    guide_italics +
  geom_bar(stat = "identity", position="stack") +
      theme(legend.position="right", legend.text=element_text(size=20), axis.title.x = element_blank(), axis.text.x = element_text(angle = 45, hjust = 1)) +
  ylab("% Abundance") +
  facet_wrap(~Genus, scales= "free", nrow=6) +
  scale_fill_manual(values = PhylaPalette) 
#
time<-format(Sys.time(),"%d_%m_%Y")
Title <- paste("Abundances_2_Genus_Relative_RARE_Strain_3")
FileName <- paste(Title,time,".jpg")
jpeg(file=FileName, width = 13000, height = 6000, res = 500)
par(mfrow = c(1,1))
alpha2 + theme(strip.text.x = element_text(size = 14, color = "black")) + theme(axis.text=element_text(size=14),
        axis.title=element_text(size=18,face="bold"))
dev.off()   


#### BARPLOT
#### BARPLOT
#### BARPLOT
#
# To group samples by strain type (skip if i want all individual samples)
merged <- merge_samples(class4.clean.sub, "strain.type")
sample_data(merged)$strain.type <- levels(sample_data(class4.clean.sub)$strain.type)
#
#
# Transform counts in relative abundance and select most abundant families
percent <- transform_sample_counts(merged, function(x) 100 * x/sum(x))
sample_data(percent)$strain.type <- as.factor(sample_data(percent)$strain.type)
#
#
#FILL MISSING TAXONOMY
tax <- data.frame(tax_table(percent))
#
tax.clean <- data.frame(row.names = row.names(tax),
Kingdom = str_replace(tax[,1], "D_0__",""),
Phylum = str_replace(tax[,2], "D_1__",""),
Class = str_replace(tax[,3], "D_2__",""),
Order = str_replace(tax[,4], "D_3__",""),
Family = str_replace(tax[,5], "D_4__",""),
Genus = str_replace(tax[,6], "D_5__",""),
Species = str_replace(tax[,7], "D_6__",""),
stringsAsFactors = FALSE)
tax.clean[is.na(tax.clean)] <- ""
#
for (i in 1:7){ tax.clean[,i] <- as.character(tax.clean[,i])}
####### Fille holes in the tax table
tax.clean[is.na(tax.clean)] <- ""
for (i in 1:nrow(tax.clean)){
#  
if (tax.clean[i,2] == ""){
kingdom <- paste("Kingdom_", tax.clean[i,1], sep = "")
tax.clean[i, 2:7] <- kingdom
} else if (tax.clean[i,3] == ""){
phylum <- paste("Phylum_", tax.clean[i,2], sep = "")
tax.clean[i, 3:7] <- phylum
} else if (tax.clean[i,4] == ""){
class <- paste("Class_", tax.clean[i,3], sep = "")
tax.clean[i, 4:7] <- class
} else if (tax.clean[i,5] == ""){
order <- paste("Order_", tax.clean[i,4], sep = "")
tax.clean[i, 5:7] <- order
} else if (tax.clean[i,6] == ""){
family <- paste("Family_", tax.clean[i,5], sep = "")
tax.clean[i, 6:7] <- family
} else if (tax.clean[i,7] == ""){
tax.clean$Species[i] <- paste("Genus",tax.clean$Genus[i], sep = "_")
}
}
#
tax_table(percent) <- as.matrix(tax.clean)
#
##############################
#FILL MISSING TAXONOMY
#
# Export ASV Table
TopNOTUs = names(sort(taxa_sums(percent), TRUE))
newdat = prune_taxa(TopNOTUs, percent)
# newdatmelt1 <- psmelt(percent)
newdatmelt <- psmelt(newdat)
#
#write.csv(newdatmelt1,file = "FULL_OTU_TALBE_NEW_time.two.NEW_2308.csv")
#
# SHOW DIFFERENT GENERA
percent2<- percent %>% tax_glom(taxrank = "Genus", NArm = FALSE)
#
TopNOTUs = names(sort(taxa_sums(percent2), TRUE)[1:20])
newdat = prune_taxa(TopNOTUs, percent2)
# newdatmelt1 <- psmelt(percent)
newdatmelt1 <- psmelt(newdat)
##
getPalette = colorRampPalette(rev(brewer.pal(12, "Paired"))) 
PhylaPalette = getPalette(20)
#
alpha2 <- ggplot(newdatmelt1, aes(x = strain.type, y = Abundance, fill = Genus)) +
    theme_bw() +
    guide_italics +
  geom_bar(stat = "identity", position="stack") +
      theme(legend.position="right", legend.text=element_text(size=20), axis.title.x = element_blank(), axis.text.x = element_text(angle = 45, hjust = 1)) +
  ylab("% Abundance") +
  facet_wrap(~group, scales= "free") +
  scale_fill_manual(values = PhylaPalette) 
#
time<-format(Sys.time(),"%d_%m_%Y")
Title <- paste("Abundances_2_Genus_Relative_RARE_HeatTol_32")
FileName <- paste(Title,time,".jpg")
jpeg(file=FileName, width = 10000, height = 3500, res = 700)
par(mfrow = c(1,1))
alpha2 + theme(strip.text.x = element_text(size = 8, color = "black")) + theme(axis.text=element_text(size=14),
        axis.title=element_text(size=14,face="bold"))
dev.off()   
####################


### EXPORT OTU TABLE FOR ABUNDANCE DATA
### EXPORT OTU TABLE FOR ABUNDANCE DATA
### EXPORT OTU TABLE FOR ABUNDANCE DATA
percent <- transform_sample_counts(merged_data, function(x) 100 * x/sum(x))
#
#FILL MISSING TAXONOMY
tax <- data.frame(tax_table(percent))
#
tax.clean <- data.frame(row.names = row.names(tax),
Kingdom = str_replace(tax[,1], "D_0__",""),
Phylum = str_replace(tax[,2], "D_1__",""),
Class = str_replace(tax[,3], "D_2__",""),
Order = str_replace(tax[,4], "D_3__",""),
Family = str_replace(tax[,5], "D_4__",""),
Genus = str_replace(tax[,6], "D_5__",""),
Species = str_replace(tax[,7], "D_6__",""),
stringsAsFactors = FALSE)
tax.clean[is.na(tax.clean)] <- ""
#
for (i in 1:7){ tax.clean[,i] <- as.character(tax.clean[,i])}
####### Fille holes in the tax table
tax.clean[is.na(tax.clean)] <- ""
for (i in 1:nrow(tax.clean)){
#  
if (tax.clean[i,2] == ""){
kingdom <- paste("Kingdom_", tax.clean[i,1], sep = "")
tax.clean[i, 2:7] <- kingdom
} else if (tax.clean[i,3] == ""){
phylum <- paste("Phylum_", tax.clean[i,2], sep = "")
tax.clean[i, 3:7] <- phylum
} else if (tax.clean[i,4] == ""){
class <- paste("Class_", tax.clean[i,3], sep = "")
tax.clean[i, 4:7] <- class
} else if (tax.clean[i,5] == ""){
order <- paste("Order_", tax.clean[i,4], sep = "")
tax.clean[i, 5:7] <- order
} else if (tax.clean[i,6] == ""){
family <- paste("Family_", tax.clean[i,5], sep = "")
tax.clean[i, 6:7] <- family
} else if (tax.clean[i,7] == ""){
tax.clean$Species[i] <- paste("Genus",tax.clean$Genus[i], sep = "_")
}
}
#
tax_table(percent) <- as.matrix(tax.clean)
#
##############################
#FILL MISSING TAXONOMY
#
# Export ASV Table
TopNOTUs = names(sort(taxa_sums(percent), TRUE))
newdat = prune_taxa(TopNOTUs, percent)
# newdatmelt1 <- psmelt(percent)
newdatmelt <- psmelt(newdat)
write.csv(newdatmelt,file = "FULL_OTU_TALBE_NEW_time.two.NEW_2308_MERGED_DATA2.csv")
```


```
###################### ADDITIONAL BAR PLOTS ######################
###################### ADDITIONAL BAR PLOTS ######################
###################### ADDITIONAL BAR PLOTS ######################

################## CLASS
################## CLASS
################## CLASS
# ALTERNATIVE PLOT
# ABUNDANCE PLOT WITH RELATIVE ABUNDANCE
# REFERENCE
# https://mibwurrepo.github.io/Microbial-bioinformatics-introductory-course-Material-2018/composition-plots.html
merged2 <- merge_samples(class4.clean.sub, "strain.type")
sample_data(merged2)$strain.type <- levels(sample_data(class4.clean.sub)$strain.type)
# Replace empty lines, otherwise "script out of bonds"
tax_table(merged2)[tax_table(merged2)[,"Class"]== "","Class"] <- "Unmatched_class"
ps1.com.fam <- microbiome::aggregate_top_taxa(merged2, "Class", top = 15)
#
#mycolors <- c(colorRampPalette(c("blue", "white", "red"))(n=12))
#
getPalette = colorRampPalette((brewer.pal(30, "Paired"))) 
PhylaPalette = getPalette(16)
#
#devtools::install_github("microsud/microbiomeutilities")
#library(microbiomeutilities)
#ps1.com.fam <- aggregate_top_taxa2(merged2, top = 10, "Family")
ps1.com.fam.rel <- microbiome::transform(ps1.com.fam, "compositional")
ps1.com.fam.rel@phy_tree <- NULL
#
alpha1 <- plot_composition(ps1.com.fam.rel, fill="Class") + 
  theme(legend.position = "bottom") +
  #scale_fill_brewer("Family", palette = "Paired") +
  scale_fill_manual(values = PhylaPalette) +
    theme_bw() +
  guide_italics +
  ylab("% Abundance of top 15 bacterial classes") +
  theme(legend.position="right", legend.text=element_text(size=20), axis.title.x = element_blank(), axis.text.x = element_text(angle = 45, hjust = 1)) 
  #facet_grid(~xlabel, scales = "free") +
  #facet_wrap(~"SS                                                                                              WT", scales= "free_x", nrow=1) +
#
time<-format(Sys.time(),"%d_%m_%Y")
Title <- paste("Abundances_3_Classes_TOP5_2_RARE")
FileName <- paste(Title,time,".jpg")
jpeg(file=FileName, width = 8000, height = 3000, res = 500)
par(mfrow = c(1,1))
alpha1 + theme(axis.text=element_text(size=17),
        axis.title=element_text(size=17,face="bold")) #+ scale_fill_manual(values = c("#7433A2",  "#A35E60", "#D1362F", "#CB9E23", "#AEA8A8", "#76A08A", "#27223C", "#CECD7B", "#7496D9", "#C7CEF6", "red", "blue", "yellow", "#1C366B", "#541F12","#F24D29", "#2ca1db", "#FCD16B", "#CC8B3C", "#112047"))
dev.off()   


################## FAMILY
################## FAMILY
################## FAMILY
# ALTERNATIVE PLOT
# ABUNDANCE PLOT WITH RELATIVE ABUNDANCE
# REFERENCE
# https://mibwurrepo.github.io/Microbial-bioinformatics-introductory-course-Material-2018/composition-plots.html
merged2 <- merge_samples(class4.clean.sub, "strain.type")
sample_data(merged2)$strain.type <- levels(sample_data(class4.clean.sub)$strain.type)
# Replace empty lines, otherwise "script out of bonds"
tax_table(merged2)[tax_table(merged2)[,"Family"]== "","Family"] <- "Unmatched_family"
ps1.com.fam <- microbiome::aggregate_top_taxa(merged2, "Family", top = 20)
#
#mycolors <- c(colorRampPalette(c("blue", "white", "red"))(n=12))
#
getPalette = colorRampPalette(brewer.pal(15, "Paired")) 
PhylaPalette = getPalette(21)
#
#devtools::install_github("microsud/microbiomeutilities")
#library(microbiomeutilities)
#ps1.com.fam <- aggregate_top_taxa2(merged2, top = 10, "Family")
ps1.com.fam.rel <- microbiome::transform(ps1.com.fam, "compositional")
ps1.com.fam.rel@phy_tree <- NULL
#
alpha1 <- plot_composition(ps1.com.fam.rel, fill="Family") + 
  theme(legend.position = "bottom") +
  #scale_fill_brewer("Family", palette = "Paired") +
  scale_fill_manual(values = PhylaPalette) +
    theme_bw() +
  guide_italics +
  ylab("% Abundance of top 20 bacterial families") +
  theme(legend.position="right", legend.text=element_text(size=20), axis.title.x = element_blank(), axis.text.x = element_text(angle = 45, hjust = 1)) 
  #facet_grid(~xlabel, scales = "free") +
  #facet_wrap(~"SS                                                                                              WT", scales= "free_x", nrow=1) +
#
time<-format(Sys.time(),"%d_%m_%Y")
Title <- paste("Abundances_3_Family_TOP5_2_RARE")
FileName <- paste(Title,time,".jpg")
jpeg(file=FileName, width = 10000, height = 3000, res = 500)
par(mfrow = c(1,1))
alpha1 + theme(axis.text=element_text(size=17),
        axis.title=element_text(size=17,face="bold")) #+ scale_fill_manual(values = c("#F24D29", "#2ca1db", "#FCD16B", "#CC8B3C", "#112047", "#7433A2",  "#A35E60", "#D1362F", "#CB9E23", "#AEA8A8", "#76A08A", "#27223C", "#CECD7B", "#7496D9", "#C7CEF6", "#2E604A", "#1C366B", "#541F12", "#1C366B", "#541F12"))
dev.off()   


################## GENERA
################## GENERA
################## GENERA
# BAR PLOT FIGURE FINAL
# ABUNDANCE PLOT WITH RELATIVE ABUNDANCE
# REFERENCE
# https://mibwurrepo.github.io/Microbial-bioinformatics-introductory-course-Material-2018/composition-plots.html
merged2 <- merge_samples(class4.clean.sub, "strain.type")
sample_data(merged2)$strain.type <- levels(sample_data(class4.clean.sub)$strain.type)
# Replace empty lines, otherwise "script out of bonds"
tax_table(merged2)[tax_table(merged2)[,"Genus"]== "","Genus"] <- "Unmatched_genus"
ps1.com.fam <- microbiome::aggregate_top_taxa(merged2, "Genus", top = 20)
#
#mycolors <- c(colorRampPalette(c("blue", "white", "red"))(n=12))
#
getPalette = colorRampPalette(rev(brewer.pal(12, "Paired"))) 
PhylaPalette = getPalette(21)
#
#devtools::install_github("microsud/microbiomeutilities")
#library(microbiomeutilities)
#ps1.com.fam <- aggregate_top_taxa2(merged2, top = 10, "Family")
ps1.com.fam.rel <- microbiome::transform(ps1.com.fam, "compositional")
ps1.com.fam.rel@phy_tree <- NULL
#
alpha1 <- plot_composition(ps1.com.fam.rel, fill="Genus") + 
  theme(legend.position = "bottom") +
  #scale_fill_brewer("Family", palette = "Paired") +
  scale_fill_manual(values = PhylaPalette) +
    theme_bw() +
  guide_italics +
  ylab("% Abundance of top 20 bacterial genera") +
  theme(legend.position="right", legend.text=element_text(size=20), axis.title.x = element_blank(), axis.text.x = element_text(angle = 45, hjust = 1)) 
time<-format(Sys.time(),"%d_%m_%Y")
Title <- paste("Abundances_3_Genera_TOP5_2_RARE")
FileName <- paste(Title,time,".jpg")
jpeg(file=FileName, width = 10000, height = 3000, res = 500)
par(mfrow = c(1,1))
alpha1 + theme(axis.text=element_text(size=17),
        axis.title=element_text(size=17,face="bold")) + theme(strip.text.x = element_text(size = 18, color = "black", face = "bold")) #+ scale_fill_manual(values = c("#7433A2",  "#A35E60", "#D1362F", "#CB9E23", "#AEA8A8", "#76A08A", "#27223C", "#CECD7B", "#7496D9", "#C7CEF6", "red", "blue", "yellow", "#1C366B", "#541F12","#F24D29", "#2ca1db", "#FCD16B", "#CC8B3C", "#112047"))
dev.off()
```


```
##################### CORE TAXA 1 ######################
##################### CORE TAXA 1 ######################
##################### CORE TAXA 1 ######################

### CORE TAXA 
# https://github.com/joey711/phyloseq/issues/293

# CREATE NEW VARIABLES $CoreTaxa
variable1 = as.character(get_variable(class4.clean.sub, "strain.type"))
variable2 = as.character(get_variable(class4.clean.sub, "temperature"))
variable3 = as.character(get_variable(class4.clean.sub, "replicate.number"))
sample_data(class4.clean.sub)$CoreTaxa <- mapply(paste0, variable1, variable2, variable3, 
    collapse = "_")
merge_samples(class4.clean.sub, "CoreTaxa")
#
sample_data(class4.clean.sub)$CoreTaxa <- as.factor(sample_data(class4.clean.sub)$CoreTaxa)
#
sample_variables(class4.clean.sub)


merged <- merge_samples(class4.clean.sub, "CoreTaxa")
sample_data(merged)$CoreTaxa <- levels(sample_data(class4.clean.sub)$CoreTaxa)
#
#
# Transform counts in relative abundance and select most abundant families
percent <- transform_sample_counts(merged, function(x) 100 * x/sum(x))
sample_data(percent)$CoreTaxa <- as.factor(sample_data(percent)$CoreTaxa)
#
#
#FILL MISSING TAXONOMY
tax <- data.frame(tax_table(percent))
#
tax.clean <- data.frame(row.names = row.names(tax),
Kingdom = str_replace(tax[,1], "D_0__",""),
Phylum = str_replace(tax[,2], "D_1__",""),
Class = str_replace(tax[,3], "D_2__",""),
Order = str_replace(tax[,4], "D_3__",""),
Family = str_replace(tax[,5], "D_4__",""),
Genus = str_replace(tax[,6], "D_5__",""),
Species = str_replace(tax[,7], "D_6__",""),
stringsAsFactors = FALSE)
tax.clean[is.na(tax.clean)] <- ""
#
for (i in 1:7){ tax.clean[,i] <- as.character(tax.clean[,i])}
####### Fille holes in the tax table
tax.clean[is.na(tax.clean)] <- ""
for (i in 1:nrow(tax.clean)){
#  
if (tax.clean[i,2] == ""){
kingdom <- paste("Kingdom_", tax.clean[i,1], sep = "")
tax.clean[i, 2:7] <- kingdom
} else if (tax.clean[i,3] == ""){
phylum <- paste("Phylum_", tax.clean[i,2], sep = "")
tax.clean[i, 3:7] <- phylum
} else if (tax.clean[i,4] == ""){
class <- paste("Class_", tax.clean[i,3], sep = "")
tax.clean[i, 4:7] <- class
} else if (tax.clean[i,5] == ""){
order <- paste("Order_", tax.clean[i,4], sep = "")
tax.clean[i, 5:7] <- order
} else if (tax.clean[i,6] == ""){
family <- paste("Family_", tax.clean[i,5], sep = "")
tax.clean[i, 6:7] <- family
} else if (tax.clean[i,7] == ""){
tax.clean$Species[i] <- paste("Genus",tax.clean$Genus[i], sep = "_")
}
}
#
tax_table(percent) <- as.matrix(tax.clean)
#
##############################
#FILL MISSING TAXONOMY
#
# Export ASV Table
TopNOTUs = names(sort(taxa_sums(percent), TRUE))
newdat = prune_taxa(TopNOTUs, percent)
# newdatmelt1 <- psmelt(percent)
newdatmelt <- psmelt(newdat)
#
write.csv(newdatmelt,file = "FULL_OTU_TALBE_NEW_20220214_SUPPLEMENT_CORETAXA_2022_TEST.csv")
```


```
##################### CORE TAXA 2 ######################
##################### CORE TAXA 2 ######################
##################### CORE TAXA 2 ######################

###########################################################################
# TAXA DISTRIBUTION AMONG STRAINS
###########################################################################
#install.packages("eulerr") # If not installed
library(eulerr)
#BiocManager::install("microbiome")
library(microbiome)
#devtools::install_github('microsud/microbiomeutilities')
library(microbiomeutilities)
#library(gplots)

#############################################################
# CORE MICROBIOME
#############################################################

# https://microbiome.github.io/tutorials/core_venn.html
table(meta(class4.clean.sub)$group, useNA = "always")

psdata.sub.rel <- microbiome::transform(class4.clean.sub, "compositional")
psdata.sub.rel2 <- prune_taxa(taxa_sums(psdata.sub.rel) > 0, psdata.sub.rel)
print(psdata.sub.rel2)


# CORE TAXA
# CORE TAXA
# CORE TAXA
core.members_WT_SS <- core_members(psdata.sub.rel2, detection = 0, prevalence = 98/100)
view(core.members_WT_SS)

SS_samples <- subset_samples(psdata.sub.rel2, group %in% c("Selected"))
core.members_SS <- core_members(SS_samples, detection = 0, prevalence = 97/100)
view(core.members_SS)

WT_samples <- subset_samples(class4.clean, group %in% c("WildType"))
core.members_WT <- core_members(WT_samples, detection = 0, prevalence = 97/100)
view(core.members_WT)


# ALTERNATIVE METHOD
# GET CORE ASVs FOR WT and SS together
# GET CORE ASVs FOR WT and SS together
# GET CORE ASVs FOR WT and SS together
Samples_for_Core <- unique(as.character(meta(psdata.sub.rel2)$group))
print(Samples_for_Core)


######
list_core3 <- c() # an empty object to store information
#
for (n in Samples_for_Core){ # for each variable n in Samples_for_Core
    #print(paste0("Identifying Core Taxa for ", n))
    #
    ps.sub <- subset_samples(psdata.sub.rel2, group == n) # Choose sample from Samples_for_Core by n
    #
    core_m <- core_members(ps.sub, # ps.sub is phyloseq selected with only samples from g 
                           detection = 0, # 0.001 in at least 90% samples 
                           prevalence = 0.97)
    print(paste0("No. of core taxa in ", n, " : ", length(core_m))) # print core taxa identified in each Samples_for_Core
    list_core3[[n]] <- core_m # add to a list core taxa for each group.
    #print(list_core)
}

print(list_core3)

WT_SS_core_df <- ldply (list_core3, data.frame)
WT_SS_core_df_2 <- table(WT_SS_core_df$X..i..) %>% sort() %>% ldply (data.frame)
WT_SS_core_allStrains <- WT_SS_core_df_2 %>% filter_at(vars(2:2), any_vars(. > 1))
WT_SS_core_IndividualStrains_temp <- WT_SS_core_df_2 %>% filter_at(vars(2:2), any_vars(. < 2))
WT_SS_core_IndividualStrains1 <- filter(WT_SS_core_df, X..i.. %in% WT_SS_core_IndividualStrains_temp$.id)
WT_SS_core_IndividualStrains2 <- table(WT_SS_core_IndividualStrains1$.id) %>% sort() %>% ldply (data.frame)

print.data.frame(WT_SS_core_allStrains) %>% nrow
print.data.frame(WT_SS_core_IndividualStrains2)
print.data.frame(WT_SS_core_IndividualStrains1)

capture.output(table(df$X..i..) %>% sort(), file = "Venndiagram_ALL_core.txt")

### VENN DIAGRAM
#https://microbiome.github.io/tutorials/core_venn.html
# ALTERNATIVE VENN
# http://www.interactivenn.net/
#library(VennDiagram)

plot(venn(list_core3))


# ADD ADDITONAL NAMES TO Taxa
# ADD ADDITONAL NAMES TO Taxa
# ADD ADDITONAL NAMES TO Taxa
# remove species name and add genus instad to ASVs
#FILL MISSING TAXONOMY
tax <- data.frame(tax_table(psdata.sub.rel2))
#
tax.clean <- data.frame(row.names = row.names(tax),
Kingdom = str_replace(tax[,1], "D_0__",""),
Phylum = str_replace(tax[,2], "D_1__",""),
Class = str_replace(tax[,3], "D_2__",""),
Order = str_replace(tax[,4], "D_3__",""),
Family = str_replace(tax[,5], "D_4__",""),
Genus = str_replace(tax[,6], "D_5__",""),
Species = str_replace(tax[,7], "D_6__",""),
stringsAsFactors = FALSE)
tax.clean[is.na(tax.clean)] <- ""
#
for (i in 1:7){ tax.clean[,i] <- as.character(tax.clean[,i])}
####### Fille holes in the tax table
tax.clean[is.na(tax.clean)] <- ""
for (i in 1:nrow(tax.clean)){
#  
if (tax.clean[i,2] == ""){
kingdom <- paste("Kingdom_", tax.clean[i,1], sep = "")
tax.clean[i, 2:7] <- kingdom
} else if (tax.clean[i,3] == ""){
phylum <- paste("Phylum_", tax.clean[i,2], sep = "")
tax.clean[i, 3:7] <- phylum
} else if (tax.clean[i,4] == ""){
class <- paste("Class_", tax.clean[i,3], sep = "")
tax.clean[i, 4:7] <- class
} else if (tax.clean[i,5] == ""){
order <- paste("Order_", tax.clean[i,4], sep = "")
tax.clean[i, 5:7] <- order
} else if (tax.clean[i,6] == ""){
family <- paste("Family_", tax.clean[i,5], sep = "")
tax.clean[i, 6:7] <- family
} else if (tax.clean[i,7] == ""){
tax.clean$Species[i] <- paste("Genus",tax.clean$Genus[i], sep = "_")
}
}
#
tax_table(psdata.sub.rel2) <- as.matrix(tax.clean)
tax_table(psdata.sub.rel2) <- tax_table(psdata.sub.rel2)[,1:6]
coral.sub.rel.f <- format_to_besthit(psdata.sub.rel2)
taxa_names(coral.sub.rel.f)[1:5]
#


list_core <- c() # an empty object to store information
#
for (n in Samples_for_Core){ # for each variable n in Samples_for_Core
    #print(paste0("Identifying Core Taxa for ", n))
    #
    ps.sub <- subset_samples(coral.sub.rel.f, group == n) # Choose sample from Samples_for_Core by n
    #
    core_m <- core_members(ps.sub, # ps.sub is phyloseq selected with only samples from g 
                           detection = 0.00000001, # 0.001 in at least 90% samples 
                           prevalence = 0.97)
    print(paste0("No. of core taxa in ", n, " : ", length(core_m))) # print core taxa identified in each Samples_for_Core
    list_core[[n]] <- core_m # add to a list core taxa for each group.
    #print(list_core)
}

print(list_core)
######################################################################
######################################################################
######################################################################


# PLOT RELATIVE ABUNDANCE OF CORE TAXA
# PLOT RELATIVE ABUNDANCE OF CORE TAXA
# PLOT RELATIVE ABUNDANCE OF CORE TAXA


#Get ASV names that were core too subset taxa for plot
list <- do.call(c, list(list_core3$Selected, list_core3$WildType)) %>% unique()

#### GROUP
# To group samples by strain type (skip if i want all individual samples)
merged <- merge_samples(class4.clean.sub, "strain.type")
sample_data(merged)$strain.type <- levels(sample_data(class4.clean.sub)$strain.type)
#
#
# Transform counts in relative abundance and select most abundant families
percent <- transform_sample_counts(merged, function(x) 100 * x/sum(x))
sample_data(percent)$strain.type <- as.factor(sample_data(percent)$strain.type)
#
#
#FILL MISSING TAXONOMY
tax <- data.frame(tax_table(percent))
#
tax.clean <- data.frame(row.names = row.names(tax),
Kingdom = str_replace(tax[,1], "D_0__",""),
Phylum = str_replace(tax[,2], "D_1__",""),
Class = str_replace(tax[,3], "D_2__",""),
Order = str_replace(tax[,4], "D_3__",""),
Family = str_replace(tax[,5], "D_4__",""),
Genus = str_replace(str_replace(str_replace(tax[,6], "D_5__uncultured_","ASV_"), "D_5__uncultured ","ASV_"), "D_5__","ASV_"),
Species = str_replace(tax[,7], "D_6__",""),
stringsAsFactors = FALSE)
tax.clean[is.na(tax.clean)] <- ""
#
for (i in 1:7){ tax.clean[,i] <- as.character(tax.clean[,i])}
####### Fille holes in the tax table
tax.clean[is.na(tax.clean)] <- ""
for (i in 1:nrow(tax.clean)){
#  
if (tax.clean[i,2] == ""){
kingdom <- paste("Kingdom_", tax.clean[i,1], sep = "")
tax.clean[i, 2:7] <- kingdom
} else if (tax.clean[i,3] == ""){
phylum <- paste("Phylum_", tax.clean[i,2], sep = "")
tax.clean[i, 3:7] <- phylum
} else if (tax.clean[i,4] == ""){
class <- paste("Class_", tax.clean[i,3], sep = "")
tax.clean[i, 4:7] <- class
} else if (tax.clean[i,5] == ""){
order <- paste("Order_", tax.clean[i,4], sep = "")
tax.clean[i, 5:7] <- order
} else if (tax.clean[i,6] == ""){
family <- paste("Family_", tax.clean[i,5], sep = "")
tax.clean[i, 6:7] <- family
} else if (tax.clean[i,7] == ""){
tax.clean$Species[i] <- paste("Genus",tax.clean$Genus[i], sep = "_")
}
}
#
tax_table(percent) <- as.matrix(tax.clean)
#
#
# SUBSET CORE TAXA FROM TABLE FOR PLOTTING
TopNOTUs = list #c("3f04430bc4eb0055b83e52a5468f2527", "92d6ffe25278cf4129bc66bfa1d6579b", "dfe02187aa7832d02e723ab6593a1cbd", "1ac5b8d681aef2a2e7246d3b86ad3d8b", "273a94b43baaa36f6d355ffa9e801a1b", "3c2ae8043f8dfc767653c8c306688e74", "40fbd3a7827374ed6cfacced463d80ef", "4a6626c191f1e73a77d26912889dcc9a", "4bd24aae96bcf09372f2c781c32db368", "50bb7f8fe47e8dfef2396f53622589b0", "5f2e9c1f54abfeb4330c917ec662041f", "5ff4e59898d66dd4864478a50556e451", "66986c4feae7f80b67f29196bef82163", "73f5bbe3b46eb015602e5bb77ffcb145", "8bf587c7640b64e109cd87f63effc923", "8f9d2a619b60c9e7bf2599c58f59b6c2", "bc62c2b699ff3ab4aa0fb330a0d36a75", "db710575c907df4ced02a3465fa38028", "e35676a9186a6be974d9296e68d0b084", "efeeaf66b9a004b23a2df59cc6475b1e", "f0239ba674bd407e955efb1813658fd8", "f5ba0f1e87d281f02e744f545baf4c38", "fdc2d924c41c68f31236d9b0b9d94dbb")
#
newdat = prune_taxa(TopNOTUs, percent)
newdatmelt1 <- psmelt(newdat)
#
#write.csv(newdatmelt1,file = "NEW_CORE_TAXA_TABLE_2022_3.csv")
#
# REORDER TAXA ON PLOT
newdatmelt2 <- newdatmelt1 %>% mutate(across(Genus, factor, levels=c("ASV_Algiphilus", "ASV_Gammaproteobacterium_44", "ASV_Hyphobacterium", "ASV_Roseitalea", "ASV_Rickettsiales_AB1", "ASV_Flexithrix", "ASV_Gammaproteobacterium_pItb-vmat-80", "ASV_Kiloniellaceae", "ASV_Labrenzia", "ASV_Marinobacter", "ASV_Reichenbachiella", "ASV_SM1A02", "ASV_Sphingobacteriales_NS11-12 ", "ASV_Actinomarinales_4", "ASV_Alphaproteobacterium_21", "ASV_AT-s3-44", "ASV_Fabibacter", "ASV_Gammaproteobacterium_20", "ASV_Gemmatimonadetes bacterium", "ASV_Ilumatobacteraceae", "ASV_Magnetospiraceae", "ASV_Simkaniaceae", "ASV_Tropicimonas")))
#
#
getPalette = colorRampPalette(rev(brewer.pal(12, "Paired"))) 
PhylaPalette = getPalette(25)
#
alpha2 <- ggplot(newdatmelt2, aes(x = strain.type, y = Abundance, fill = Genus)) +
    theme_bw() +
    guide_italics +
  geom_bar(stat = "identity", position="stack") +
      theme(legend.position="right", legend.text=element_text(size=20), axis.title.x = element_blank(), axis.text.x = element_text(angle = 45, hjust = 1)) +
  ylab("% Abundance") +
  facet_wrap(~Genus, scales= "free", nrow=6) +
  #scale_fill_manual(values = PhylaPalette) 
  scale_fill_manual(values = c("#fec185",  "#fec185", "#fec185", "#fec185", "#808080", "#808080", "#808080", "#808080", "#808080", "#808080", "#808080", "#808080", "#808080", "#b8cee6", "#b8cee6","#b8cee6", "#b8cee6", "#b8cee6", "#b8cee6", "#b8cee6","#b8cee6", "#b8cee6", "#b8cee6", "#b8cee6", "#b8cee6","#b8cee6", "#b8cee6", "#b8cee6", "#b8cee6","#b8cee6", "#b8cee6", "#b8cee6", "#b8cee6", "#b8cee6","#b8cee6", "#b8cee6", "#b8cee6","#b8cee6", "#b8cee6", "#b8cee6", "#b8cee6", "#b8cee6"))
#
time<-format(Sys.time(),"%d_%m_%Y")
Title <- paste("Abundances_2_Genus_Relative_RARE_CORE_ASVs")
FileName <- paste(Title,time,".jpg")
jpeg(file=FileName, width = 15000, height = 7000, res = 500)
par(mfrow = c(1,1))
alpha2 + theme(strip.text.x = element_text(size = 16, color = "black")) + theme(axis.text=element_text(size=14),axis.title=element_text(size=20,face="bold"))
dev.off()
```


```
############### DIFFERENTIAL ABUNDANCE ANALYSIS - DESEQ2 #############
############### DIFFERENTIAL ABUNDANCE ANALYSIS - DESEQ2 #############
############### DIFFERENTIAL ABUNDANCE ANALYSIS - DESEQ2 #############


# COMPARE SPECIES ASSOCIATED
# THREE DIFFERENT GROUPS: SS+, SS- and WT

# PREPARE DATA


# Transform counts in relative abundance and select most abundant families
#
merged_data2 <- merged_data
#FILL MISSING TAXONOMY
tax <- data.frame(tax_table(merged_data2))
#
tax.clean <- data.frame(row.names = row.names(tax),
Kingdom = str_replace(tax[,1], "D_0__",""),
Phylum = str_replace(tax[,2], "D_1__",""),
Class = str_replace(tax[,3], "D_2__",""),
Order = str_replace(tax[,4], "D_3__",""),
Family = str_replace(tax[,5], "D_4__",""),
Genus = str_replace(tax[,6], "D_5__","ASV_"),
Species = str_replace(tax[,7], "D_6__",""),
stringsAsFactors = FALSE)
tax.clean[is.na(tax.clean)] <- ""
#
for (i in 1:7){ tax.clean[,i] <- as.character(tax.clean[,i])}
####### Fille holes in the tax table
tax.clean[is.na(tax.clean)] <- ""
for (i in 1:nrow(tax.clean)){
#  
if (tax.clean[i,2] == ""){
kingdom <- paste("Kingdom_", tax.clean[i,1], sep = "")
tax.clean[i, 2:7] <- kingdom
} else if (tax.clean[i,3] == ""){
phylum <- paste("Phylum_", tax.clean[i,2], sep = "")
tax.clean[i, 3:7] <- phylum
} else if (tax.clean[i,4] == ""){
class <- paste("Class_", tax.clean[i,3], sep = "")
tax.clean[i, 4:7] <- class
} else if (tax.clean[i,5] == ""){
order <- paste("Order_", tax.clean[i,4], sep = "")
tax.clean[i, 5:7] <- order
} else if (tax.clean[i,6] == ""){
family <- paste("Family_", tax.clean[i,5], sep = "")
tax.clean[i, 6:7] <- family
} else if (tax.clean[i,7] == ""){
tax.clean$Species[i] <- paste("Genus",tax.clean$Genus[i], sep = "_")
}
}
#
tax_table(merged_data2) <- as.matrix(tax.clean)
#
##############################


# EITHER: DESEQ ON GENERA ... or DESEQ on ASVs... then continue after ### START
#merged_data_genus <- merged_data2 %>% tax_glom(taxrank = "Genus", NArm = FALSE)
GenusFiltered <- subset_taxa(merged_data, Genus != "NA")
physeqGenus = tax_glom(GenusFiltered, "Genus")

psdata.sub_SS <- physeqGenus
data.deseq2_SS <- phyloseq_to_deseq2(psdata.sub_SS, ~ 0 + heat.tolerance) 
data.deseq2_SS$heat.tolerance <- relevel(data.deseq2_SS$heat.tolerance, ref = "WT")


# OR: DESEQ ON ASVs
psdata.sub_SS <- merged_data2
data.deseq2_SS <- phyloseq_to_deseq2(psdata.sub_SS, ~ 0 + heat.tolerance) 
data.deseq2_SS$heat.tolerance <- relevel(data.deseq2_SS$heat.tolerance, ref = "WT")


############# START
############# START
############# START

data.deseq2.test_SS <- DESeq(data.deseq2_SS, test="Wald", fitType="parametric") # performing statistical test
head(data.deseq2.test_SS)
resultsNames(data.deseq2.test_SS)


# APPLY DIFFERENT CONTRASTS
res1 <- results(data.deseq2.test_SS, cooksCutoff = FALSE, contrast=c("heat.tolerance","SS_minus","SS_plus"))
head(res1)

res2 <- results(data.deseq2.test_SS, cooksCutoff = FALSE,  contrast=c("heat.tolerance","SS_minus","WT"))
head(res2)

res3 <- results(data.deseq2.test_SS, cooksCutoff = FALSE, contrast=c("heat.tolerance","SS_plus","WT"))
head(res3)

res4 <- results(data.deseq2.test_SS, cooksCutoff = FALSE, contrast=c(1,-0.5,-0.5))
head(res4)


# To interpret the results, you can type:
mcols(res1)$description # CHECK THIS ONE HERE

mcols(res2)$description # DISREGARD

mcols(res3)$description # DISREGARD

mcols(res4)$description # CHECK THIS ONE HERE


# PLOT THE DIFFERENT CONTRASTS
alpha <- 0.001 # setting significance threshold value
logfold1 <- 2
logfold2 <- -2

sigtab_res0 <- res1[which(res1$log2FoldChange < logfold2 | res1$log2FoldChange > logfold1), ] # putting in a table significant taxa
sigtab_res1 <- sigtab_res0[which(sigtab_res0$padj < alpha), ] # putting in a table significant taxa
#sigtab_res1 <- res1[which(res1$padj < alpha), ] # putting in a table significant taxa
sigtab_res1 <- cbind(as(sigtab_res1, "data.frame"), as(tax_table(psdata.sub_SS)[rownames(sigtab_res1), ], "matrix"))

#dim(sigtab)
#
x = tapply(sigtab_res1$log2FoldChange, sigtab_res1$Family, function(x) max(x))
x = sort(x, FALSE)
sigtab_res1$Family = factor(as.character(sigtab_res1$Family), levels=names(x))
x = tapply(sigtab_res1$log2FoldChange, sigtab_res1$Genus, function(x) max(x))
x = sort(x, TRUE)
sigtab_res1$Genus = factor(as.character(sigtab_res1$Genus), levels=names(x))
#
# REFERENCE
# https://joey711.github.io/phyloseq-extensions/DESeq2.html
# 
#
TEST_SS <- ggplot(sigtab_res1, aes(x=log2FoldChange, y=Genus, color=Class)) + geom_point(size=4) +
  theme_bw() +
  theme(axis.text.x = element_text(angle = -90, hjust = 0, vjust=0.5))
#
TEST3_SS <- TEST_SS + scale_color_manual(values = c("#F24D29", "#C7CEF6", "#FCD16B", "#E5C4A1", "#7496D2", "#B62A3D",  "#541F12", "#CC8B3C", "#7496D2", "#CB9E23", "#541F12", "#76A08A", "#27223C", "#CECD7B", "#7496D2", "#C7CEF6", "#2E604A", "#1C366B", "#1DACE8","#F24D29", "#E5C4A1", "#FCD16B", "#CC8B3C", "#D8A49B", "#B62A3D",  "#A35E60", "#541F12", "#D1362F", "#CB9E23", "#AEA8A8", "#76A08A", "#27223C", "#CECD7B", "#7496D2", "#C7CEF6", "#2E604A", "#1C366B", "#1DACE8", "#CECD7B", "#7496D2", "#C7CEF6", "#2E604A", "#1C366B", "#1DACE8"))
#
time<-format(Sys.time(),"%d_%m_%Y")
Title <- paste("DeSeq2_Genus2_SScomp_res1a")
FileName <- paste(Title,time,".jpg")
#jpeg(file=FileName, width = 5220, height = 1280, res = 200)
jpeg(file=FileName, width = 4000, height = 3500, res = 500)
TEST3_SS
dev.off() 


# And you will obtain a list, where the second element is:
# "[2] log2 fold change (MAP): Treatment B vs A"

# Since it is for example written "B vs A" (and not "A vs B"),
# it means that "B" is the reference treatment to which A is compared.

# So a LogFoldChange of x means that the abundance of that particular taxa
# is 2^(x) times greater in B than in A. If x is positive,
# the taxa is more abundant in B, while if x is negative, 2^x < 1 and thus the taxa
# will be more abundant in A.
#positive means more abundant in Wildtype but if negative taxa is more abundant in selected 

posigtab_res1 = sigtab_res1[sigtab_res1[, "log2FoldChange"] > 0, ]
posigtab_res1 = posigtab_res1[, c("baseMean", "log2FoldChange", "lfcSE", "padj", "Phylum", "Class", "Family", "Genus")]
#output results 
write.csv(as.data.frame(posigtab_res1), file="deseq-res1_SSminus-enriched-results.csv")

posigtab2_res1 = sigtab_res1[sigtab_res1[, "log2FoldChange"] < 0, ]
posigtab2_res1 = posigtab2_res1[, c("baseMean", "log2FoldChange", "lfcSE", "padj", "Phylum", "Class", "Family", "Genus")]
#output results 
write.csv(as.data.frame(posigtab2_res1), file="deseq-res1_SSplus-enriched-results.csv")

# To interpret the results, you can type:
mcols(res1)$description
# WRITE BOTH OUTPUTS
write.csv(as.data.frame(sigtab_res1), file="deseq_res1_COMP_threeway_SSminus_SSplus_TEST.csv")


#
# PLOT THE DIFFERENT CONTRASTS
alpha <- 0.001 # setting significance threshold value
logfold1 <- 2
logfold2 <- -2
#
sigtab_res00 <- res4[which(res4$log2FoldChange < logfold2 | res4$log2FoldChange > logfold1), ] # putting in a table significant taxa
sigtab_res4 <- sigtab_res00[which(sigtab_res00$padj < alpha), ] # putting in a table significant taxa
#sigtab_res4 <- res4[which(res4$padj < alpha), ] # putting in a table significant taxa
sigtab_res4 <- cbind(as(sigtab_res4, "data.frame"), as(tax_table(psdata.sub_SS)[rownames(sigtab_res4), ], "matrix"))
sigtab_res4
#dim(sigtab)
#
x = tapply(sigtab_res4$log2FoldChange, sigtab_res4$Family, function(x) max(x))
x = sort(x, FALSE)
sigtab_res4$Family = factor(as.character(sigtab_res4$Family), levels=names(x))
x = tapply(sigtab_res4$log2FoldChange, sigtab_res4$Genus, function(x) max(x))
x = sort(x, TRUE)
sigtab_res4$Genus = factor(as.character(sigtab_res4$Genus), levels=names(x))
#
# REFERENCE
# https://joey711.github.io/phyloseq-extensions/DESeq2.html
#
#
TEST_SS <- ggplot(sigtab_res4, aes(x=log2FoldChange, y=Genus, color=Class)) + geom_point(size=4) +
  theme_bw() +
  theme(axis.text.x = element_text(angle = -90, hjust = 0, vjust=0.5))
#
TEST3_SS <- TEST_SS + scale_color_manual(values = c("#FF0000", "#C7CEF6", "#FCD16B", "#E5C4A1", "#7496D2", "#B62A3D",  "#A35E60", "#CC8B3C", "#1C366B", "#808080", "#541F12", "#76A08A", "#D1362F", "#CECD7B", "#1DACE8"))#, "#C7CEF6", "#2E604A", "#1C366B", "#1DACE8","#F24D29", "#E5C4A1", "#FCD16B", "#CC8B3C", "#D8A49B", "#B62A3D",  "#A35E60", "#541F12", "#D1362F", "#CB9E23", "#AEA8A8", "#76A08A", "#27223C", "#CECD7B", "#7496D2", "#C7CEF6", "#2E604A", "#1C366B", "#1DACE8", "#CECD7B", "#7496D2", "#C7CEF6", "#2E604A", "#1C366B", "#1DACE8"))#"#F24D29", "#E5C4A1", "#FCD16B", "#CC8B3C", "#D8A49B", "#B62A3D",  "#A35E60", "#541F12", "#D1362F", "#CB9E23", "#AEA8A8", "#76A08A", "#27223C", "#CECD7B", "#7496D2", "#C7CEF6", "#2E604A", "#1C366B", "#1DACE8","#F24D29", "#E5C4A1", "#FCD16B", "#CC8B3C", "#D8A49B", "#B62A3D",  "#A35E60", "#541F12", "#D1362F", "#CB9E23", "#AEA8A8", "#76A08A", "#27223C", "#CECD7B", "#7496D2", "#C7CEF6", "#2E604A", "#1C366B", "#1DACE8","#F24D29", "#E5C4A1", "#FCD16B", "#CC8B3C", "#D8A49B", "#B62A3D",  "#A35E60", "#541F12", "#D1362F", "#CB9E23", "#AEA8A8", "#76A08A", "#27223C", "#CECD7B", "#7496D2", "#C7CEF6", "#2E604A", "#1C366B", "#1DACE8", "#CECD7B", "#7496D2", "#C7CEF6", "#2E604A", "#1C366B", "#1DACE8","#C7CEF6", "#2E604A", "#1C366B", "#1DACE8", "#CECD7B", "#7496D2", "#C7CEF6", "#2E604A", "#1C366B", "#1DACE8", "#CC8B3C"))
#
time<-format(Sys.time(),"%d_%m_%Y")
Title <- paste("DeSeq2_Genus2_SScomp_res4a")
FileName <- paste(Title,time,".jpg")
#jpeg(file=FileName, width = 5220, height = 1280, res = 200)
jpeg(file=FileName, width = 5000, height = 6000, res = 500)
TEST3_SS
dev.off() 


# CHECK ASVs that were significantly enriched in SS non-conferring 
# cleaning up the table for legibility.
posigtab_res4 = sigtab_res4[sigtab_res4[, "log2FoldChange"] > 0, ]
posigtab_res4 = posigtab_res4[, c("baseMean", "log2FoldChange", "lfcSE", "padj", "Phylum", "Class", "Family", "Genus")]

# write output
write.csv(as.data.frame(posigtab_res4), file="deseq-res4_WT_together-enriched-results.csv")


# CHECK ASVs that were significantly enriched in SS conferring tolerance
# cleaning up the table for legibility.
posigtab2_res4 = sigtab_res4[sigtab_res4[, "log2FoldChange"] < 0, ]
posigtab2_res4 = posigtab2_res4[, c("baseMean", "log2FoldChange", "lfcSE", "padj", "Phylum", "Class", "Family", "Genus")]

# write output
write.csv(as.data.frame(posigtab2_res4), file="deseq-res4_SS_together-enriched-results.csv")

mcols(res4)$description

# WRITE BOTH OUTPUTS
write.csv(as.data.frame(sigtab_res4), file="deseq_res4_COMP_threeway_WT_vs_SS.csv")
```


```
########## PHSYSIOLOGICAL DATA ##########
########## PHSYSIOLOGICAL DATA ##########
########## PHSYSIOLOGICAL DATA ##########


####### CELL DENSITIES - PLOT STRAINS ########
####### CELL DENSITIES - PLOT STRAINS ########
####### CELL DENSITIES - PLOT STRAINS ########

df_tidy <- read.csv("../../PHYSIOLOGY/Strains_CellD.csv")


# MAKE THIS DATA INPUT
#df_tidy <- gather(df_merged, Cell, Ratio, -id,-Time)

df_tidy <- unite(df_tidy, unique_id, c(id, Cell), sep="_", remove = FALSE)

df_tidy_mean2 <- df_tidy %>%
    group_by(Time, id) %>%
    summarise(n = n(), mean=mean(Ratio))
#
df_tidy_mean2 <- df_tidy %>%
    filter(!is.na(Ratio)) %>%
    group_by(Time, id) %>%
    summarise(n = n(), mean=mean(Ratio))
#
df_tidy_mean2 <- df_tidy %>%
    filter(!is.na(Ratio)) %>%
    group_by(Time, id) %>%
    summarise(n = n(),
           mean = mean(Ratio),
         median = median(Ratio),
             sd = sd(Ratio))

df_tidy_mean2 <- df_tidy %>%
    filter(!is.na(Ratio)) %>%
    group_by(Time, id) %>%
    summarise(n = n(),
           Cell_Density = mean(Ratio),
         median = median(Ratio),
             sd = sd(Ratio)) %>%
    mutate(sem = sd / sqrt(n - 1),
      CI_lower = Cell_Density + qt((1-0.95)/2, n - 1) * sem,
      CI_upper = Cell_Density - qt((1-0.95)/2, n - 1) * sem,
      CI_upper1 = Cell_Density + sd,
      CI_lower1 = Cell_Density - sd)


df_tidy_mean2$id2 <- gsub(" ","",as.character(df_tidy_mean2$id))
df_tidy_mean2$id2 <- gsub("31","",as.character(df_tidy_mean2$id2))
df_tidy_mean2$id2 <- gsub("27","",as.character(df_tidy_mean2$id2))
df_tidy_mean2$id2 <- as.factor((df_tidy_mean2)$id2)
df_tidy_mean2$id2 <- fct_relevel(df_tidy_mean2$id2, rev)
#
df_tidy_mean2$id3 <- gsub("[0-9]","",as.character(df_tidy_mean2$id2))
df_tidy_mean2$id3 <- as.factor((df_tidy_mean2)$id3)
df_tidy_mean2$id3 <- fct_relevel(df_tidy_mean2$id3, sort)
df_tidy_mean2$id3 <- fct_relevel(df_tidy_mean2$id3, rev)


color_list <- c("#F24D29", "#8d202f", "#F24D29", "#8d202f", "#F24D29",  "#8d202f", "#F24D29", "#8d202f", "#F24D29","#8d202f", "#F24D29", "#8d202f", "#F24D29", "#8d202f", "#F24D29","#8d202f", "#F24D29", "#8d202f", "#F24D29","#8d202f", "#1C366B", "#1DACE8", "#1C366B","#1DACE8", "#1C366B", "#1DACE8", "#1C366B", "#1DACE8", "#1C366B", "#1DACE8", "#1C366B", "#1DACE8", "#1C366B","#1DACE8", "#1C366B", "#1DACE8", "#1C366B", "#1DACE8")

#color_list <- c("#E5C4A1", "#FCD16B", "#CC8B3C", "#D8A49B", "#B62A3D",  "#A35E60", "#541F12", "#D1362F", "#CB9E23","#F24D29", "#E5C4A1", "#FCD16B", "#CC8B3C", "#D8A49B", "#B62A3D",  "#A35E60", "#541F12", "#D1362F", "#CB9E23","#AEA8A8", "#76A08A", "#27223C", "#CECD7B", "#7496D2", "#C7CEF6", "#2E604A", "#1C366B", "#1DACE8","#76A08A", "#AEA8A8", "#76A08A", "#27223C", "#CECD7B", "#7496D2", "#C7CEF6", "#2E604A", "#1C366B", "#1DACE8")


time<-format(Sys.time(),"%d_%m_%Y")
Title <- paste("PhysiologicalData_CellD3")
FileName <- paste(Title,time,".jpg")
#jpeg(file=FileName, width = 2220, height = 1280, res = 200)
jpeg(file=FileName, width = 6000, height = 5000, res = 890)
ggplot(df_tidy_mean2, aes(x=Time, y=Cell_Density, color = id))+
  geom_line(aes(x=Time, y=Cell_Density, color=id), size=1, show.legend = F)+
  #geom_ribbon(aes(ymin=CI_lower1,ymax=CI_upper1,fill=id),color="grey",alpha=0.5) +
  theme_light(base_size = 16) + 
  scale_fill_manual(values=color_list) +
  #xlim(1,22) +
  scale_color_manual(values=color_list) +
  theme_bw(base_size = 16)
dev.off() 


time<-format(Sys.time(),"%d_%m_%Y")
Title <- paste("PhysiologicalData_CellD4")
FileName <- paste(Title,time,".jpg")
#jpeg(file=FileName, width = 2220, height = 1280, res = 200)
jpeg(file=FileName, width = 7000, height = 3000, res = 700)
ggplot(df_tidy_mean2, aes(x=Time, y=Cell_Density, color = id))+
  geom_line(aes(x=Time, y=Cell_Density, color=id), size=1, show.legend = F) +
  #geom_ribbon(aes(ymin=CI_lower1,ymax=CI_upper1,fill=id),color="grey",alpha=0.4) +
  scale_fill_manual(values=color_list) + scale_color_manual(values=color_list) +
  facet_grid(.~id3) +
  ylim(0,1200000) +
  theme_bw(base_size = 16)
dev.off() 

# STRAINS NEXT TO EACH OTHER
plot_CellD3 <- ggplot(df_tidy_mean2, aes(x=Time, y=Cell_Density, color = id))+
  geom_line(aes(x=Time, y=Cell_Density, color=id), size=1, show.legend = F) +
  #geom_ribbon(aes(ymin=CI_lower1,ymax=CI_upper1,fill=id),color="grey",alpha=0.4) +
  scale_fill_manual(values=color_list) + scale_color_manual(values=color_list) +
  facet_grid(.~id3) +
  #ylim(0,1200000) +
  theme_bw(base_size = 16)


ggplot(df_tidy_mean, aes(x=Time, y=Cell_Density, color = id1))+
  geom_line(aes(x=Time, y=Cell_Density, color=id1), size=1)+
  geom_ribbon(aes(ymin=CI_lower1,ymax=CI_upper1,fill=id1),color="grey",alpha=0.4) +
  scale_fill_manual(values=color_list) + scale_color_manual(values=color_list) +
  facet_grid(.~id2) +
  #xlim(1,22) +
  theme_bw(base_size = 16)


ggplot(df_tidy_mean, aes(x=Time, y=Cell_Density, color = id1))+
  geom_line(aes(x=Time, y=Cell_Density, color=id1), size=1)+
  geom_ribbon(aes(ymin=CI_lower1,ymax=CI_upper1,fill=id1),color="grey",alpha=0.4) +
  scale_fill_manual(values=color_list) + scale_color_manual(values=color_list) +
  facet_grid(id2~.)+ theme(legend.position="none") +
  #xlim(1,22) +
  theme_bw(base_size = 16)
```


```
########## PHSYSIOLOGICAL DATA ##########
########## PHSYSIOLOGICAL DATA ##########
########## PHSYSIOLOGICAL DATA ##########


####### CELL DENSITIES - PLOT AVERAGE ########
####### CELL DENSITIES - PLOT AVERAGE ########
####### CELL DENSITIES - PLOT AVERAGE ########

# https://thenode.biologists.com/visualizing-data-one-more-time/education/

df_tidy <- read.csv("../../PHYSIOLOGY/Strains_CellD.csv")


# MAKE THIS DATA INPUT
# df_tidy <- gather(df_merged, Cell, Ratio, -id,-Time)

df_tidy <- unite(df_tidy, unique_id, c(id1, Cell), sep="_", remove = FALSE)

df_tidy_mean <- df_tidy %>%
    group_by(Time, id1) %>%
    summarise(n = n(), mean=mean(Ratio))
#
df_tidy_mean <- df_tidy %>%
    filter(!is.na(Ratio)) %>%
    group_by(Time, id1) %>%
    summarise(n = n(), mean=mean(Ratio))
#
df_tidy_mean <- df_tidy %>%
    filter(!is.na(Ratio)) %>%
    group_by(Time, id1) %>%
    summarise(n = n(),
           mean = mean(Ratio),
         median = median(Ratio),
             sd = sd(Ratio))

df_tidy_mean <- df_tidy %>%
    filter(!is.na(Ratio)) %>%
    group_by(Time, id1) %>%
    summarise(n = n(),
           Cell_Density = mean(Ratio),
         median = median(Ratio),
             sd = sd(Ratio)) %>%
    mutate(sem = sd / sqrt(n - 1),
      CI_lower = Cell_Density + qt((1-0.95)/2, n - 1) * sem,
      CI_upper = Cell_Density - qt((1-0.95)/2, n - 1) * sem,
      CI_upper1 = Cell_Density + sd,
      CI_lower1 = Cell_Density - sd)

df_tidy_mean$id2 <- gsub(" ","",as.character(df_tidy_mean$id1))
df_tidy_mean$id2 <- gsub("31","",as.character(df_tidy_mean$id2))
df_tidy_mean$id2 <- gsub("27","",as.character(df_tidy_mean$id2))
#
df_tidy_mean$id2 <- as.factor((df_tidy_mean)$id2)
df_tidy_mean$id2 <- fct_relevel(df_tidy_mean$id2, rev)


color_list1 <- c("#ff9f1c", "#f34c28","#008dc7", "#4f8696")


time<-format(Sys.time(),"%d_%m_%Y")
Title <- paste("PhysiologicalData_CellD2")
FileName <- paste(Title,time,".jpg")
#jpeg(file=FileName, width = 2220, height = 1280, res = 200)
jpeg(file=FileName, width = 7000, height = 5000, res = 890)
ggplot(df_tidy_mean, aes(x=Time, y=Cell_Density, color = id1))+
  geom_line(aes(x=Time, y=Cell_Density, color=id1), size=1)+
  geom_ribbon(aes(ymin=CI_lower1,ymax=CI_upper1,fill=id1),color="grey",alpha=0.4) +
  theme_light(base_size = 16) + 
  scale_fill_manual(values=color_list1) +
  #xlim(1,22) +
  scale_color_manual(values=color_list1) +
  theme_bw(base_size = 16)
dev.off() 


# GENERATE PLOTS WITH GRAPHS NEXT TO EACH OTHER
plot_CellD_1 <- ggplot(df_tidy_mean, aes(x=Time, y=Cell_Density, color = id1))+
  geom_line(aes(x=Time, y=Cell_Density, color=id1), size=1)+
  #geom_smooth(aes(ymin=CI_lower1,ymax=CI_upper1,fill=id1),color="grey",alpha=0.4) +
  geom_ribbon(aes(ymin=CI_lower1,ymax=CI_upper1,fill=id1),color="grey",alpha=0.4) +
  scale_fill_manual(values=color_list1) + scale_color_manual(values=color_list1) +
  facet_grid(.~id2) +
  #ylim(0,1200000) +
  theme_bw(base_size = 16)


time<-format(Sys.time(),"%d_%m_%Y")
Title <- paste("PhysiologicalData_CellD")
FileName <- paste(Title,time,".jpg")
#jpeg(file=FileName, width = 2220, height = 1280, res = 200)
jpeg(file=FileName, width = 7000, height = 3000, res = 700)
ggplot(df_tidy_mean, aes(x=Time, y=Cell_Density, color = id1))+
  geom_line(aes(x=Time, y=Cell_Density, color=id1), size=1)+
  #geom_smooth(aes(ymin=CI_lower1,ymax=CI_upper1,fill=id1),color="grey",alpha=0.4) +
  geom_ribbon(aes(ymin=CI_lower1,ymax=CI_upper1,fill=id1),color="grey",alpha=0.4) +
  scale_fill_manual(values=color_list1) + scale_color_manual(values=color_list1) +
  facet_grid(.~id2) +
  ylim(0,1200000) +
  theme_bw(base_size = 16)
dev.off() 


# USE THIS CODE FOR GRAPHS WITH A STACKED OUTPUT
plot_CellD_1 <- ggplot(df_tidy_mean, aes(x=Time, y=Cell_Density, color = id1))+
  geom_line(aes(x=Time, y=Cell_Density, color=id1), size=1)+
  geom_ribbon(aes(ymin=CI_lower1,ymax=CI_upper1,fill=id1),color="grey",alpha=0.4) +
  scale_fill_manual(values=color_list1) + scale_color_manual(values=color_list1) +
  facet_grid(id2~.)+ theme(legend.position="none") +
  #xlim(1,22) +
  theme_bw(base_size = 16)
```


```
########## PHSYSIOLOGICAL DATA ##########
########## PHSYSIOLOGICAL DATA ##########
########## PHSYSIOLOGICAL DATA ##########


####### MAXIMUM QUANTUM YIELD - Plot Strains ########
####### MAXIMUM QUANTUM YIELD - Plot Strains ########
####### MAXIMUM QUANTUM YIELD - Plot Strains ########

df_MQY <- read.csv("../../PHYSIOLOGY/Strains_MQY.csv")


# MAKE THIS DATA INPUT
#df_MQY <- gather(df_merged, Cell, Yield, -id,-Time)

df_MQY <- unite(df_MQY, unique_id, c(id, Cell), sep="_", remove = FALSE)

df_MQY_mean2 <- df_MQY %>%
    group_by(Time, id) %>%
    summarise(n = n(), mean=mean(Yield))

#
df_MQY_mean2 <- df_MQY %>%
    filter(!is.na(Yield)) %>%
    group_by(Time, id) %>%
    summarise(n = n(), mean=mean(Yield))

#
df_MQY_mean2 <- df_MQY %>%
    filter(!is.na(Yield)) %>%
    group_by(Time, id) %>%
    summarise(n = n(),
           mean = mean(Yield),
         median = median(Yield),
             sd = sd(Yield))

df_MQY_mean2 <- df_MQY %>%
    filter(!is.na(Yield)) %>%
    group_by(Time, id) %>%
    summarise(n = n(),
           MQY = mean(Yield),
         median = median(Yield),
             sd = sd(Yield)) %>%
    mutate(sem = sd / sqrt(n - 1),
      CI_lower = MQY + qt((1-0.95)/2, n - 1) * sem,
      CI_upper = MQY - qt((1-0.95)/2, n - 1) * sem,
      CI_upper1 = MQY + sd,
      CI_lower1 = MQY - sd)


df_MQY_mean2$id2 <- gsub(" ","",as.character(df_MQY_mean2$id))
df_MQY_mean2$id2 <- gsub("31","",as.character(df_MQY_mean2$id2))
df_MQY_mean2$id2 <- gsub("27","",as.character(df_MQY_mean2$id2))
df_MQY_mean2$id2 <- as.factor((df_MQY_mean2)$id2)
df_MQY_mean2$id2 <- fct_relevel(df_MQY_mean2$id2, rev)
#
df_MQY_mean2$id3 <- gsub("[0-9]","",as.character(df_MQY_mean2$id2))
df_MQY_mean2$id3 <- as.factor((df_MQY_mean2)$id3)
df_MQY_mean2$id3 <- fct_relevel(df_MQY_mean2$id3, sort)
df_MQY_mean2$id3 <- fct_relevel(df_MQY_mean2$id3, rev)


color_list <- c("#F24D29", "#8d202f", "#F24D29", "#8d202f", "#F24D29",  "#8d202f", "#F24D29", "#8d202f", "#F24D29","#8d202f", "#F24D29", "#8d202f", "#F24D29", "#8d202f", "#F24D29","#8d202f", "#F24D29", "#8d202f", "#F24D29","#8d202f", "#1C366B", "#1DACE8", "#1C366B","#1DACE8", "#1C366B", "#1DACE8", "#1C366B", "#1DACE8", "#1C366B", "#1DACE8", "#1C366B", "#1DACE8", "#1C366B","#1DACE8", "#1C366B", "#1DACE8", "#1C366B", "#1DACE8")

#color_list <- c("#E5C4A1", "#FCD16B", "#CC8B3C", "#D8A49B", "#B62A3D",  "#A35E60", "#541F12", "#D1362F", "#CB9E23","#F24D29", "#E5C4A1", "#FCD16B", "#CC8B3C", "#D8A49B", "#B62A3D",  "#A35E60", "#541F12", "#D1362F", "#CB9E23","#AEA8A8", "#76A08A", "#27223C", "#CECD7B", "#7496D2", "#C7CEF6", "#2E604A", "#1C366B", "#1DACE8","#76A08A", "#AEA8A8", "#76A08A", "#27223C", "#CECD7B", "#7496D2", "#C7CEF6", "#2E604A", "#1C366B", "#1DACE8")


time<-format(Sys.time(),"%d_%m_%Y")
Title <- paste("PhysiologicalData_MQY3")
FileName <- paste(Title,time,".jpg")
#jpeg(file=FileName, width = 2220, height = 1280, res = 200)
jpeg(file=FileName, width = 6000, height = 5000, res = 890)
ggplot(df_MQY_mean2, aes(x=Time, y=MQY, color = id))+
  geom_line(aes(x=Time, y=MQY, color=id), size=1, show.legend = F)+
  #geom_ribbon(aes(ymin=CI_lower1,ymax=CI_upper1,fill=id),color="grey",alpha=0.5) +
  theme_light(base_size = 16) + 
  scale_fill_manual(values=color_list) +
  #xlim(1,22) +
  scale_color_manual(values=color_list) +
  theme_bw(base_size = 16)
dev.off() 


time<-format(Sys.time(),"%d_%m_%Y")
Title <- paste("PhysiologicalData_MQY4")
FileName <- paste(Title,time,".jpg")
#jpeg(file=FileName, width = 2220, height = 1280, res = 200)
jpeg(file=FileName, width = 7000, height = 3000, res = 700)
ggplot(df_MQY_mean2, aes(x=Time, y=MQY, color = id))+
  geom_line(aes(x=Time, y=MQY, color=id), size=1, show.legend = F) +
  #geom_ribbon(aes(ymin=CI_lower1,ymax=CI_upper1,fill=id),color="grey",alpha=0.4) +
  scale_fill_manual(values=color_list) + scale_color_manual(values=color_list) +
  facet_grid(.~id3) +
  #ylim(0,1200000) +
  theme_bw(base_size = 16)
dev.off() 

# STRAINS NEXT TO EACH OTHER
plot_MQY3 <- ggplot(df_MQY_mean2, aes(x=Time, y=MQY, color = id))+
  geom_line(aes(x=Time, y=MQY, color=id), size=1, show.legend = F) +
  #geom_ribbon(aes(ymin=CI_lower1,ymax=CI_upper1,fill=id),color="grey",alpha=0.4) +
  scale_fill_manual(values=color_list) + scale_color_manual(values=color_list) +
  facet_grid(.~id3) +
  #ylim(0,1200000) +
  theme_bw(base_size = 16)


ggplot(df_MQY_mean, aes(x=Time, y=MQY, color = id1))+
  geom_line(aes(x=Time, y=MQY, color=id1), size=1)+
  geom_ribbon(aes(ymin=CI_lower1,ymax=CI_upper1,fill=id1),color="grey",alpha=0.4) +
  scale_fill_manual(values=color_list) + scale_color_manual(values=color_list) +
  facet_grid(.~id2) +
  #xlim(1,22) +
  theme_bw(base_size = 16)


ggplot(df_MQY_mean, aes(x=Time, y=MQY, color = id1))+
  geom_line(aes(x=Time, y=MQY, color=id1), size=1)+
  geom_ribbon(aes(ymin=CI_lower1,ymax=CI_upper1,fill=id1),color="grey",alpha=0.4) +
  scale_fill_manual(values=color_list) + scale_color_manual(values=color_list) +
  facet_grid(id2~.)+ theme(legend.position="none") +
  #xlim(1,22) +
  theme_bw(base_size = 16)
```


```
########## PHSYSIOLOGICAL DATA ##########
########## PHSYSIOLOGICAL DATA ##########
########## PHSYSIOLOGICAL DATA ##########


####### MQY - PLOT AVERAGE ########
####### MQY - PLOT AVERAGE ########
####### MQY - PLOT AVERAGE ########

# https://thenode.biologists.com/visualizing-data-one-more-time/education/

df_MQY <- read.csv("../../PHYSIOLOGY/Strains_MQY.csv")


# MAKE THIS DATA INPUT
#df_MQY <- gather(df_merged, Cell, Yield, -id,-Time)

df_MQY <- unite(df_MQY, unique_id, c(id1, Cell), sep="_", remove = FALSE)

df_MQY_mean <- df_MQY %>%
    group_by(Time, id1) %>%
    summarise(n = n(), mean=mean(Yield))
#
df_MQY_mean <- df_MQY %>%
    filter(!is.na(Yield)) %>%
    group_by(Time, id1) %>%
    summarise(n = n(), mean=mean(Yield))
#
df_MQY_mean <- df_MQY %>%
    filter(!is.na(Yield)) %>%
    group_by(Time, id1) %>%
    summarise(n = n(),
           mean = mean(Yield),
         median = median(Yield),
             sd = sd(Yield))

df_MQY_mean <- df_MQY %>%
    filter(!is.na(Yield)) %>%
    group_by(Time, id1) %>%
    summarise(n = n(),
           MQY = mean(Yield),
         median = median(Yield),
             sd = sd(Yield)) %>%
    mutate(sem = sd / sqrt(n - 1),
      CI_lower = MQY + qt((1-0.95)/2, n - 1) * sem,
      CI_upper = MQY - qt((1-0.95)/2, n - 1) * sem,
      CI_upper1 = MQY + sd,
      CI_lower1 = MQY - sd)

df_MQY_mean$id2 <- gsub(" ","",as.character(df_MQY_mean$id1))
df_MQY_mean$id2 <- gsub("31","",as.character(df_MQY_mean$id2))
df_MQY_mean$id2 <- gsub("27","",as.character(df_MQY_mean$id2))
#
df_MQY_mean$id2 <- as.factor((df_MQY_mean)$id2)
df_MQY_mean$id2 <- fct_relevel(df_MQY_mean$id2, rev)


color_list1 <- c("#ff9f1c", "#f34c28","#008dc7", "#4f8696")


time<-format(Sys.time(),"%d_%m_%Y")
Title <- paste("PhysiologicalData_MQY2")
FileName <- paste(Title,time,".jpg")
#jpeg(file=FileName, width = 2220, height = 1280, res = 200)
jpeg(file=FileName, width = 7000, height = 5000, res = 890)
ggplot(df_MQY_mean, aes(x=Time, y=MQY, color = id1))+
  geom_line(aes(x=Time, y=MQY, color=id1), size=1)+
  geom_ribbon(aes(ymin=CI_lower1,ymax=CI_upper1,fill=id1),color="grey",alpha=0.4) +
  theme_light(base_size = 16) + 
  scale_fill_manual(values=color_list1) +
  #xlim(1,22) +
  scale_color_manual(values=color_list1) +
  theme_bw(base_size = 16)
dev.off() 


time<-format(Sys.time(),"%d_%m_%Y")
Title <- paste("PhysiologicalData_MQY")
FileName <- paste(Title,time,".jpg")
#jpeg(file=FileName, width = 2220, height = 1280, res = 200)
jpeg(file=FileName, width = 7000, height = 3000, res = 700)
ggplot(df_MQY_mean, aes(x=Time, y=MQY, color = id1))+
  geom_line(aes(x=Time, y=MQY, color=id1), size=1)+
  #geom_smooth(aes(ymin=CI_lower1,ymax=CI_upper1,fill=id1),color="grey",alpha=0.4) +
  geom_ribbon(aes(ymin=CI_lower1,ymax=CI_upper1,fill=id1),color="grey",alpha=0.4) +
  scale_fill_manual(values=color_list1) + scale_color_manual(values=color_list1) +
  facet_grid(.~id2) +
  #ylim(0,1200000) +
  theme_bw(base_size = 16)
dev.off() 

ggplot(df_MQY_mean, aes(x=Time, y=MQY, color = id1))+
  geom_line(aes(x=Time, y=MQY, color=id1), size=1)+
  geom_ribbon(aes(ymin=CI_lower1,ymax=CI_upper1,fill=id1),color="grey",alpha=0.4) +
  scale_fill_manual(values=color_list1) + scale_color_manual(values=color_list1) +
  facet_grid(.~id2) +
  #xlim(1,22) +
  theme_bw(base_size = 16)


# STACKED
plot_MQY1 <- ggplot(df_MQY_mean, aes(x=Time, y=MQY, color = id1))+
  geom_line(aes(x=Time, y=MQY, color=id1), size=1)+
  geom_ribbon(aes(ymin=CI_lower1,ymax=CI_upper1,fill=id1),color="grey",alpha=0.4) +
  scale_fill_manual(values=color_list1) + scale_color_manual(values=color_list1) +
  facet_grid(id2~.)+ theme(legend.position="none") +
  #xlim(1,22) +
  theme(legend.position = "none") +
  theme_bw(base_size = 16)


# NEXT TO EACH OTHER
plot_MQY1 <- ggplot(df_MQY_mean, aes(x=Time, y=MQY, color = id1))+
  geom_line(aes(x=Time, y=MQY, color=id1), size=1)+
  #geom_smooth(aes(ymin=CI_lower1,ymax=CI_upper1,fill=id1),color="grey",alpha=0.4) +
  geom_ribbon(aes(ymin=CI_lower1,ymax=CI_upper1,fill=id1),color="grey",alpha=0.4) +
  scale_fill_manual(values=color_list1) + scale_color_manual(values=color_list1) +
  facet_grid(.~id2) +
  theme(legend.position = "none") +
  #ylim(0,1200000) +
  theme_bw(base_size = 16)


### EXPORT PLOTS TOGETHER
### EXPORT PLOTS TOGETHER
### EXPORT PLOTS TOGETHER
time<-format(Sys.time(),"%d_%m_%Y")
Title <- paste("PhysiologicalData_CellD_MQY2")
FileName <- paste(Title,time,".jpg")
#jpeg(file=FileName, width = 2220, height = 1280, res = 200)
jpeg(file=FileName, width = 10000, height = 4000, res = 800)
grid.arrange(plot_CellD_1, plot_MQY1, nrow = 1)
dev.off() 
plot_MQY1

time<-format(Sys.time(),"%d_%m_%Y")
Title <- paste("PhysiologicalData_CellD_MQY3")
FileName <- paste(Title,time,".jpg")
#jpeg(file=FileName, width = 2220, height = 1280, res = 200)
jpeg(file=FileName, width = 10000, height = 7000, res = 900)
grid.arrange(plot_CellD3, plot_MQY3, nrow = 2)
dev.off() 
plot_MQY1
```


```
########## PHSYSIOLOGICAL DATA ##########
########## PHSYSIOLOGICAL DATA ##########
########## PHSYSIOLOGICAL DATA ##########


####### ROS - PLOT AVERAGE ########
####### ROS - PLOT AVERAGE ########
####### ROS - PLOT AVERAGE ########

# https://thenode.biologists.com/visualizing-data-one-more-time/education/

library(dplyr)

df_ROS <- read.csv("../../PHYSIOLOGY/ROS.csv")

df_ROS$id2 <- gsub(" ","",as.character(df_ROS$Treatment))
df_ROS$id2 <- gsub("31","",as.character(df_ROS$id2))
df_ROS$id2 <- gsub("27","",as.character(df_ROS$id2))
#
df_ROS$StrainTemp2 <- as.factor((df_ROS)$StrainTemp2)
df_ROS$StrainTemp2 <- fct_relevel(df_ROS$StrainTemp2, sort)


df_ROS$StrainTemp <- fct_relevel(df_ROS$StrainTemp, "WT10_27", "WT11_27", "WT12_27", "WT13_27", "WT14_27", "WT15_27", "WT16_27", "WT17_27","WT18_27", "WT10_31", "WT11_31", "WT12_31", "WT13_31", "WT14_31", "WT15_31", "WT16_31", "WT17_31", "WT18_31", "SS1_27", "SS2_27", "SS3_27", "SS4_27", "SS5_27", "SS6_27", "SS7_27", "SS8_27", "SS9_27", "SS19_27", "SS1_31", "SS2_31", "SS3_31", "SS4_31", "SS5_31", "SS6_31", "SS7_31", "SS8_31", "SS9_31", "SS19_31")


df_ROS$Treatment <- fct_relevel(df_ROS$Treatment, "WT 27", "WT 31", "SS 27", "SS 31")


#CellD2.lme <- lme(ROS ~ Group*Temperature, random = ~ 1|Strain, data = df_ROS, method="ML")


color_list1 <- c("#ff9f1c", "#f34c28","#008dc7", "#4f8696")
#
p<-ggplot(df_ROS, aes(x=StrainTemp, y=ROS, color=Treatment)) +
  geom_boxplot(position =position_dodge(width  =.8)) +
  scale_fill_manual(values=color_list1) + scale_color_manual(values=color_list1) +
  theme_bw(base_size = 16) +
  theme(axis.text.x = element_text(angle = 45, hjust = 1,size=8))
  #theme(legend.position="none") 
p #+ facet_wrap(~ id2)


color_list1 <- c("#008dc7", "#4f8696","#ff9f1c", "#f34c28")
#
p<-ggplot(df_ROS, aes(x=Treatment, y=ROS, color=Treatment)) +
  geom_boxplot(position =position_dodge(width  =.8)) +
  scale_fill_manual(values=color_list1) + scale_color_manual(values=color_list1) +
  theme_bw(base_size = 16) +
  theme(axis.text.x = element_text(size=14))
  #theme(legend.position="none") 
p #+ facet_wrap(~ id2)


time<-format(Sys.time(),"%d_%m_%Y")
Title <- paste("PhysiologicalData_CellD_MQY2_ROS3")
FileName <- paste(Title,time,".jpg")
#jpeg(file=FileName, width = 2220, height = 1280, res = 200)
jpeg(file=FileName, width = 7000, height = 7000, res = 800)
grid.arrange(plot_CellD_1, plot_MQY1, p, nrow = 3)
dev.off() 


color_list1 <- c("#F24D29", "#8d202f","#1C366B", "#1DACE8")
#
p<-ggplot(df_ROS, aes(x=StrainTemp, y=ROS, color=Treatment)) +
  geom_boxplot(position =position_dodge(width  =.8)) +
  scale_fill_manual(values=color_list1) + scale_color_manual(values=color_list1) +
  theme_bw(base_size = 16) +
  theme(axis.text.x = element_text(angle = 45, hjust = 1,size=8))+
  theme(legend.position="none") 
p #+ facet_wrap(~ id2)


time<-format(Sys.time(),"%d_%m_%Y")
Title <- paste("PhysiologicalData_CellD_MQY2_ROS2")
FileName <- paste(Title,time,".jpg")
#jpeg(file=FileName, width = 2220, height = 1280, res = 200)
jpeg(file=FileName, width = 7000, height = 7000, res = 800)
grid.arrange(plot_CellD3, plot_MQY3, p, nrow = 3)
dev.off()
```


```
########## PHSYSIOLOGICAL DATA ##########
########## PHSYSIOLOGICAL DATA ##########
########## PHSYSIOLOGICAL DATA ##########


########## LINEAR MIXED EFFECTS MODEL ###################
########## LINEAR MIXED EFFECTS MODEL ###################
########## LINEAR MIXED EFFECTS MODEL ###################


library (nlme)
library (lattice)
library (grid)
library (colorspace)
library (effects)
library (car)


############################################################
##### LINEAR MIXED EFFECTS MODEL - CELL DENSITIES
############################################################

CellD_data <- read.delim("../../PHYSIOLOGY/CellD_MixedEffects2.txt", header=TRUE)#, stringsAsFactors=TRUE)


str(CellD_data)
CellD_data$Ratio <- as.numeric(CellD_data$Ratio)
CellD_data$Time <- as.numeric(CellD_data$Time)
CellD_data$Temperature <- as.factor(CellD_data$Temperature)
CellD_data$Group <- as.factor(CellD_data$Group)
CellD_data$Strain <- as.factor(CellD_data$Strain)
str(CellD_data)


CellD1.lme <- lme(Ratio ~ Group*Time*Temperature, random = ~ 1|Time, data = CellD_data, method="ML")
CellD2.lme <- lme(Ratio ~ Group*Time*Temperature, random = ~ 1|Strain, data = CellD_data, method="ML")

anova(CellD1.lme, CellD2.lme)

# BEST MODEL WITH Yield3.lme

CellD4.1.lme <- lme(Ratio ~ Group*Time*Temperature, random = ~ 1|Strain, data = CellDnoZero)
anova(CellD4.1.lme)
LMEM.CellD <- anova(CellD4.1.lme)
summary(CellD4.1.lme)
#
Title <- paste("./STATS_LMEM_2022_CellD_noPseudo")
FileName <- paste(Title,time,".txt")
write.table(LMEM.CellD, file = FileName, sep = "\t")


CellD4.1.lme <- lme(Ratio ~ Group*Time*Temperature, random = ~ 1|Strain, data = CellDnoZero, control=list(msMaxIter=100,opt= "optim",msVerbose=TRUE), method="REML")
anova(CellD4.1.lme)
summary(CellD4.1.lme)

res_lme=residuals(CellD4.1.lme)
plot(res_lme)
qqnorm(res_lme)

plot(allEffects(CellD4.1.lme))


########## model VALIDATION 
time<-format(Sys.time(),"%d_%m_%Y")
Title <- paste("LMEM_CELLD_model_assumptions")
FileName <- paste(Title,time,".jpg")
#jpeg(file=FileName, width = 2220, height = 1280, res = 200)
jpeg(file=FileName, width = 2500, height = 3000, res = 400)
par(mfrow = c(3,2))
R1 <- resid(CellD4.1.lme)
plot(R1)
hist(R1, xlab = "Residuals", main = "")
plot(CellDnoZero$Group, R1, xlab = "Group", ylab = "Residuals")
plot(CellDnoZero$Temperature, R1, xlab = "Temperature", ylab = "Residuals")
plot(CellDnoZero$Time, R1, xlab = "Time", ylab = "Residuals")
qqnorm(R1)
dev.off()


CellDnoZero$Temperature<-relevel(CellDnoZero$Temperature,"31")
CellDnoZero$Group<-relevel(CellDnoZero$Group,"SS")
CellDnoZero$Group.Temp = with(CellDnoZero, interaction(Group,Temperature))
#
CellD4.1.lme <- lme(Ratio ~ Time*Group.Temp, random = ~ 1|Strain, data = CellDnoZero,control=list(msMaxIter=100,opt= "optim",msVerbose=TRUE), method="REML")
summary(CellD4.1.lme)

#
Title <- paste("./STATS_LMEM_2022_CELLD_noPseudo_RELEVEL_WT31")
FileName <- paste(Title,time,".txt")
#capture.output(summary(CellD4.1.lme),file=FileName)
write.table(test, file = FileName, sep = "\t")
```


```
########## PHSYSIOLOGICAL DATA ##########
########## PHSYSIOLOGICAL DATA ##########
########## PHSYSIOLOGICAL DATA ##########


################### LINEAR MIXED EFFECTS MODEL ###################
################### LINEAR MIXED EFFECTS MODEL ###################
################### LINEAR MIXED EFFECTS MODEL ###################

library (nlme)
library (lattice)
library (grid)
library (colorspace)
library (effects)
library (car)


############################################################
##### nlme - MAXIMUM QUANTUM YIELD
############################################################

Yield_data <- read.delim("../../PHYSIOLOGY/PAM_MixedEffects2.txt", header=TRUE)


### CHECK THAT ALL ARE FACTORS, if not MAKE THEM FACTORS
# MAKE TIME NUMERIC
str(Yield_data)

Yield_data$Yield <- as.numeric(Yield_data$Yield)
Yield_data$Time <- as.numeric(Yield_data$Time)
Yield_data$Temperature <- as.factor(Yield_data$Temperature)
Yield_data$Group <- as.factor(Yield_data$Group)
Yield_data$Strain <- as.factor(Yield_data$Strain)
#Yield_data$Period <- as.factor(Yield_data$Period)
str(Yield_data)


# MAKE DATASET WITH ZEROs REMOVED AND CHECK IF IT IMPROVES THE FIT
# YieldnoZero <- subset (Yield_data, Yield >  0)

# TRY SUITABLE MODEL AND CHECK FIT, model with lowest AIC will be considered. If negative AIC more negative = better model. YOU GET THE AIC MODELS FROM THE ANOVA MODEL COMPARISON
# COMPARE MODELS WITH MAXIMUM LIKELIHOOD "ML"
#
# FOR THE MODELS "random = ~ 1|STRAIN" => strain as random factor with random intercepts
# FOR THE MODELS "random = ~ TIME|STRAIN" => time and strain as random factors with random intercepts and random slopes.
Yield1.lme <- lme(Yield ~ Group*Time*Temperature, random = ~ 1|Time, data = Yield_data, method="ML")

Yield2.lme <- lme(Yield ~ Group*Time*Temperature, random = ~ 1|Strain, data = Yield_data, method="ML")


anova(Yield1.lme, Yield2.lme)


# COMPARE MODELS
anova(Yield1.lme, Yield2.lme)

anova(Yield3.lme, Yield4.lme)


# refit the BEST OR MODEL THAT MAKES SENSE MOST with REML, method not defined = automatically REML
Yield3.1.lme <- lme(Yield ~ Group*Time*Temperature, random = ~ 1|Strain, data = YieldnoZero)
anova(Yield3.1.lme)
LMEM.Yield <- anova(Yield3.1.lme)
summary(Yield3.1.lme)
#

Title <- paste("./STATS_LMEM_2022_Yield_noPseudo")
FileName <- paste(Title,time,".txt")
write.table(LMEM.Yield, file = FileName, sep = "\t")


# OR PUT REML IN, WRITE LIKE THIS:
Yield3.1.lme <- lme(Yield ~ Group*Time*Temperature, random = ~ 1|Strain, data = YieldnoZero, control=list(msMaxIter=100,opt= "optim",msVerbose=TRUE), method="REML")
anova(Yield3.1.lme)
#
summary(Yield3.1.lme)


# CHECK MODEL ASSUMPTIONS
res_lme=residuals(Yield3.1.lme)
plot(res_lme)
qqnorm(res_lme)

plot(allEffects(Yield3.1.lme))


# EXPORT PLOT FOR MODEL ASSUMPTIONS
########## model VALIDATION 
########## model VALIDATION 
time<-format(Sys.time(),"%d_%m_%Y")
Title <- paste("LMEM_Yield_model_assumptions")
FileName <- paste(Title,time,".jpg")
#jpeg(file=FileName, width = 2220, height = 1280, res = 200)
jpeg(file=FileName, width = 2500, height = 3000, res = 400)
par(mfrow = c(3,2))
R1 <- resid(Yield3.1.lme)
plot(R1)
hist(R1, xlab = "Residuals", main = "")
plot(YieldnoZero$Group, R1, xlab = "Group", ylab = "Residuals")
plot(YieldnoZero$Temperature, R1, xlab = "Temperature", ylab = "Residuals")
plot(YieldnoZero$Time, R1, xlab = "Time", ylab = "Residuals")
qqnorm(R1)
dev.off()

# Fig. X.Y Model validation graphs. 1) Fitted values versus residuals - homogeneity. 2) Histogram of the residuals - normality. 3) Residuals versus Origin - independence. 4) Residuals versus Treatment - independence. 5) Residuals versus Time - independence. 6) Q-Q plot - normality.


########## Multiple comparisons / adjust reference level...
# ADJUST CONTRASTS FOR "POST-HOC-STYLE" COMPARISON
YieldnoZero$Temperature<-relevel(YieldnoZero$Temperature,"27")
YieldnoZero$Group<-relevel(YieldnoZero$Group,"SS")
YieldnoZero$Group.Temp = with(YieldnoZero, interaction(Group,Temperature))
#
Yield3.2.1ST.lme <- lme(Yield ~ Time*Group.Temp, random = ~ 1|Strain, data = YieldnoZero,control=list(msMaxIter=100,opt= "optim",msVerbose=TRUE), method="REML")
summary(Yield3.2.1ST.lme)
#

Title <- paste("./STATS_LMEM_2022_YIELD_noPseudo_RELEVEL_SS27")
FileName <- paste(Title,time,".txt")
capture.output(Yield3.2.1ST.lme,file=FileName)


# ADJUST CONTRASTS FOR "POST-HOC-STYLE" COMPARISON
YieldnoZero$Temperature<-relevel(YieldnoZero$Temperature,"27") # ADJUST "27" to "31" depends on the comparison that you want
YieldnoZero$Group<-relevel(YieldnoZero$Group,"WT") # ADJUST "WT" to "SS" depends on the comparison you want to see.
YieldnoZero$Group.Temp = with(YieldnoZero, interaction(Group,Temperature))
#
Yield3.2.1ST.lme <- lme(Yield ~ Time*Group.Temp, random = ~ 1|Strain, data = YieldnoZero,control=list(msMaxIter=100,opt= "optim",msVerbose=TRUE), method="REML")
summary(Yield3.2.1ST.lme)

#The last page of the summary output should give you stats like this one here:
#   Value   Std.Error   DF  t-value p-value
#(Intercept)    0.382717    0.011142857 199 34.3464 0
#Time   -0.0077613  0.000617513 199 -12.56863   0
#Group.TempSS.31    -0.0001189  0.015286042 199 -0.00778    0.9938
#Group.TempWT.27    0.0290861   0.012216662 199 2.38086 0.0182
#Group.TempSS.27    0.0409231   0.015421018 199 2.65372 0.0086
#Time:Group.TempSS.31   0.0078022   0.000847495 199 9.20622 0
#Time:Group.TempWT.27   0.0088792   0.000869301 199 10.21415    0
#Time:Group.TempSS.27   0.0068947   0.000854284 199 8.07077 0


# EXPORT DOES NOT WORK CURRENTLY... NEED TO ADJUST TO GET THE TABLE ABOVE, BUT YOU CAN SEE THE OUTPUT IN R FOR NOW.
Title <- paste("./STATS_LMEM_2022_YIELD_noPseudo_RELEVEL_WT31")
FileName <- paste(Title,time,".txt")
capture.output(Yield3.2.1ST.lme,file=FileName)


### IF REGRESSION IS REQUIRED.. CHECK THIS OUT:
### IF REGRESSION IS REQUIRED.. CHECK THIS OUT:
### IF REGRESSION IS REQUIRED.. CHECK THIS OUT:

YieldnoZero$Temperature<-relevel(YieldnoZero$Temperature,"31")
YieldnoZero$Group<-relevel(YieldnoZero$Group,"WT")
summary(Yield3.2.1ST.lme)


# Reference level Combined West:
# Fixed effects: Yield ~ Time * Ori.Treat 
#(Intercept)        0.3827170   0.011142857 199 34.34640    0.0000
#Time               -0.0077613  0.000617513 199 -12.56863   0.0000
#Group.TempSS.31    -0.0001189  0.015286042 199 -0.00778    0.9938

############ Regressions 1st Period, y = a + bx #############
# y = 0.3827170 (0.011142857) - 0.0077613 (0.000617513) x 


## PLOT
YieldCombined <- subset(YieldnoZero, Temperature == "31")
YieldCombined$Ori.TreatCom = with(YieldCombined, interaction(Group,Temperature))
scatterplot(Yield~Time*Group, data=YieldCombined)
```


```
########## PHSYSIOLOGICAL DATA ##########
########## PHSYSIOLOGICAL DATA ##########
########## PHSYSIOLOGICAL DATA ##########


################### LINEAR MIXED EFFECTS MODEL ###################
################### LINEAR MIXED EFFECTS MODEL ###################
################### LINEAR MIXED EFFECTS MODEL ###################

library (nlme)
library (lattice)
library (grid)
library (colorspace)
library (effects)
library (car)


############################################################
##### nlme - ROS
############################################################

ROS_data <- read.delim("../../PHYSIOLOGY/ROS_MixedEffects2.txt", header=TRUE)#, stringsAsFactors=TRUE)


str(ROS_data)
ROS_data$ROS <- as.numeric(ROS_data$ROS)
ROS_data$Temperature <- as.factor(ROS_data$Temperature)
ROS_data$Group <- as.factor(ROS_data$Group)
ROS_data$Strain <- as.factor(ROS_data$Strain)
str(ROS_data)

ROS_data$ROSlog=log(ROS_data$ROS)
str(ROS_data)

ROS2.lme <- lme(ROS ~ Group*Temperature, random = ~ 1|Strain, data = ROS_data, method="ML")
ROS4.lme <- lme(ROSlog ~ Group*Temperature, random = ~ 1|Strain, data = ROS_data, method="ML")

anova(ROS2.lme)
anova(ROS4.lme)


ROS4.1.lme <- lme(ROSlog ~ Group*Temperature, random = ~ 1|Strain, data = ROS_data)
anova(ROS4.1.lme)
LMEM.ROS <- anova(ROS4.1.lme)
summary(ROS4.1.lme)

#
Title <- paste("./STATS_LMEM_2022_ROS_noPseudo")
FileName <- paste(Title,time,".txt")
write.table(LMEM.ROS, file = FileName, sep = "\t")


res_lme=residuals(ROS4.1.lme)
plot(res_lme)
qqnorm(res_lme)

plot(allEffects(ROS4.1.lme))


########## model VALIDATION 
time<-format(Sys.time(),"%d_%m_%Y")
Title <- paste("LMEM_ROS_model_assumptions")
FileName <- paste(Title,time,".jpg")
#jpeg(file=FileName, width = 2220, height = 1280, res = 200)
jpeg(file=FileName, width = 2500, height = 3000, res = 400)
par(mfrow = c(3,2))
R1 <- resid(ROS4.1.lme)
plot(R1)
hist(R1, xlab = "Residuals", main = "")
plot(ROSnoZero$Group, R1, xlab = "Group", ylab = "Residuals")
plot(ROSnoZero$Temperature, R1, xlab = "Temperature", ylab = "Residuals")
qqnorm(R1)
dev.off()


ROSnoZero$Temperature<-relevel(ROSnoZero$Temperature,"31")
ROSnoZero$Group<-relevel(ROSnoZero$Group,"WT")
ROSnoZero$Group.Temp = with(ROSnoZero, interaction(Group,Temperature))
#
ROS4.1.lme <- lme(ROSlog ~ Group.Temp, random = ~ 1|Strain, data = ROSnoZero,control=list(msMaxIter=100,opt= "optim",msVerbose=TRUE), method="REML")
summary(ROS4.1.lme)
#
Title <- paste("./STATS_LMEM_2022_ROS_noPseudo_RELEVEL_WT31")
FileName <- paste(Title,time,".txt")
capture.output(ROS4.1.lme,file=FileName)
```
